# Deconjugative α-Alkylation of Cyclohexenecarboxaldehydes: An Access to Diverse Terpenoids

**DOI:** 10.1021/acs.joc.1c00560

**Published:** 2021-06-15

**Authors:** Rachid Chahboun, José Manuel Botubol-Ares, María Jesús Durán-Peña, Fermín Jiménez, Ramón Alvarez-Manzaneda, Enrique Alvarez-Manzaneda

**Affiliations:** † Departamento de Química Orgánica, Facultad de Ciencias, Instituto de Biotecnología, Universidad de Granada, 18071 Granada, Spain; ‡ Departamento de Química Orgánica, Facultad de Ciencias, Campus Universitario Río San Pedro s/n, Torre Sur, 4a planta, University of Cádiz, Puerto Real, 11510 Cádiz, Spain; § Área de Química Orgánica, Departamento de Química y Física, Universidad de Almería, 04120 Almería, Spain

## Abstract

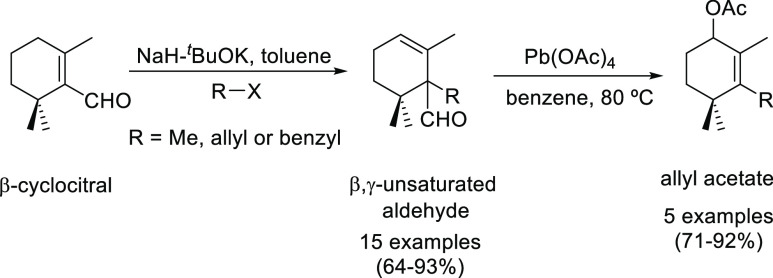

A general and efficient method for the deconjugative α-alkylation of α,β-unsaturated aldehydes promoted by a synergistic effect between ^*t*^BuOK and NaH, which considerably increases the reaction rate under mild conditions, is reported. The β,γ-unsaturated aldehyde, resulting from the α-alkylation, is transformed in high yield into the corresponding allyl acetate via a lead(IV) acetate-mediated oxidative fragmentation. This strategy could be used for the construction of the carbon skeleton of a wide variety of alkyl or arylterpenoids.

## Introduction

Terpenoids are a broad group of natural products, characterized by their structural diversity, that have found extensive use in medicine and other industries.^1−3^ However, their scarcity in their natural, marine, or terrestrial sources often forces the development of synthetic processes for their availability. Within the enormous variety of synthetic processes described to access this type of compounds, those that use simple terpenoids as a starting product to prepare more complex molecules deserve to be highlighted.^4^ This has the obvious advantage of reducing the number of synthetic sequence steps. In addition, if the terpene precursor is easily accessible, and therefore cheap, the process may have commercial interest.

**Scheme 1:**
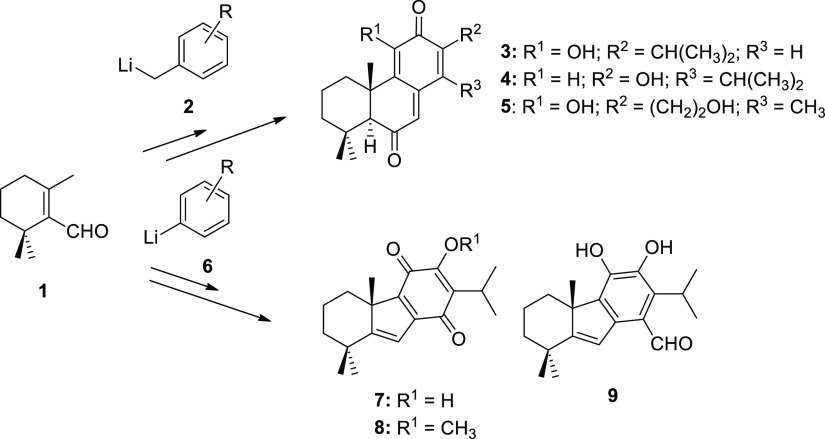
Synthesis of Terpenoids from β-Cyclocitral (**1**) and Aryllithium Derivatives

β-Cyclocitral (**1**) is a cyclic monoterpene, commercially available at a low price, which has been used as a precursor of different terpenoids (Scheme 1). In this way, the reaction of aldehyde **1** with the suitable benzyllithium derivative **2** afforded the corresponding allyl homobenzyl alcohol, the precursor of the abietane quinone taxodione (**3**)^5^ and the totarane quinone maytenoquinone (**4**),^6^ both with antitumor properties, or the antifungal tricyclic diterpenoid **5**.^7^ When β-cyclocitral (**1**) was treated with the suitable aryllithium derivative **6**, taiwaniaquinoids such as dichroanone (**7**),^8^ taiwaniaquinone H (**8**),^8^ and dichroanal B (**9**)^8b^ were obtained. These processes involve the condensation of a terpene synthon having electrophilic character with an aromatic derivative of nucleophile nature.

However, in certain cases, preparation of nucleophilic aromatic synthons became difficult because of either the substitution pattern or the presence of electrophilic groups (e.g., CN, COOR, COR, and so forth) in the aromatic ring, resulting in the consequent lengthening sequence. It is therefore of interest to investigate new approaches to avoid this inconvenience.

**Scheme 2:**
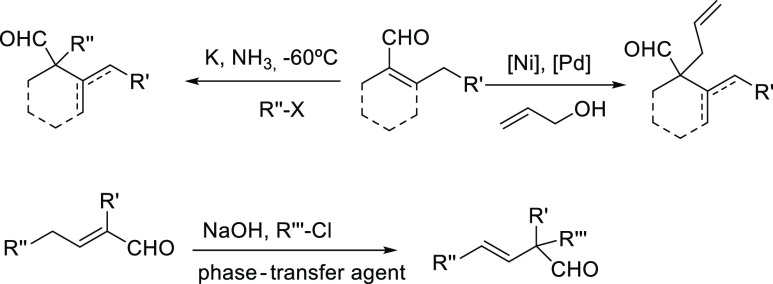
Previous Direct α-Alkylation of α,β-Unsaturated Aldehydes

The construction of the carbon skeleton of compounds such as **3–5** and **7–9** could be achieved in an alternative way by performing the α-alkylation of the α,β-unsaturated terpenic aldehyde **1** with a suitable benzyl halide to afford the corresponding arylterpenylaldehydes, followed by cyclization. Despite its synthetic potential, the deconjugative α-alkylation of aldehydes has been studied very little (Scheme 2). In a pioneer work, De Graaf et al. described the direct alkylation of 1-cyclohexene-1-carbaldehyde with different agents in liquid ammonia at −60 °C in the presence of potassium amide, affording a mixture of products.^9^ After that, a procedure to achieve the direct α-alkylation of acyclic aldehydes utilizing NaOH and a phase-transfer agent in an inert solvent has been patented.^10^ Additionally, the palladium- and nickel-catalyzed deconjugative α-allylation with allyl alcohols of aldehydes, including a few α,β-unsaturated aldehydes, has also been described (Scheme 2).^11^ Other indirect methods, such as the alkylation of the corresponding dimethylhydrazones, in the presence of lead lithium diisopropylamide (LDA), have been described.^12^ Recently, the copper-catalyzed deconjugative α-alkylation of cyclic α,β-unsaturated nitriles has been reported.^13^

**Scheme 3:**
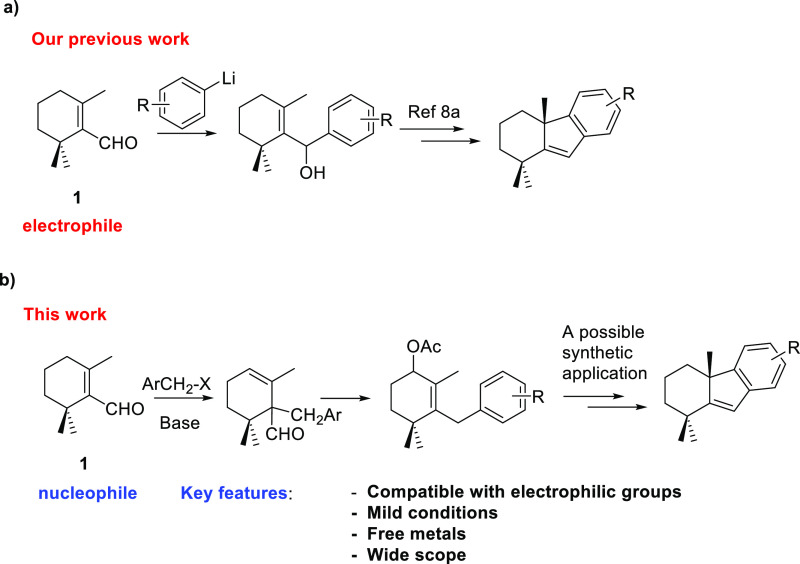
Synthetic Approaches to Terpenoids; (a) Conventional Methods for the Synthesis of Terpenoids from β-Cyclocitral (**1**) as an Electrophile; (b) Deconjugative α-Alkylation of Cyclohexene-1-carboxaldehydes for the Synthesis of Terpenoids

In this paper, we describe the deconjugative α-alkylation of cyclohexene-1-carboxaldehydes and its synthetic application as a platform to access challenging terpene frameworks. In comparison to other existing direct α-alkylation of α,β-unsaturated aldehydes (Scheme 2), this methodology occurs under milder metal-free reaction conditions with a wider scope and compatible with electrophilic groups (Scheme 3).

## Results and Discussion

Considering our working hypothesis, we initiated the study of the α-alkylation of β-cyclocitral (**1**) using allyl bromide (**10a**) as the electrophile. The reaction was performed in different solvents such as tetrahydrofuran (THF), acetonitrile, and toluene in the presence of ^*t*^BuOK as a base, and the desired α-alkylated product (±)-**11a**, formed selectively from a trisubstituted dienolate, was obtained in poor to moderate yields (Table 1, entries 1–3). A higher amount of ^*t*^BuOK did not improve the yield either (Table 1, entry 4). The addition of 18-crown-6-ether led to a mixture of (±)-**11a** and the corresponding O-alkylated derivative **12a** in THF, acetonitrile, and toluene (Table 1, entries 5–7). Then, the effect of other bases was also considered in the model reaction. LiHMDS increased the C-/O-alkylation ratio [(±)-**11a**/**12a** ratio] up to 5:1 (Table 1, entry 8). On the contrary, the deconjugative α-alkylation of **1** with LDA did not work (Table 1, entry 9). In a similar fashion, NaH led to unreacted starting material in toluene, whereas a 1:1 mixture of (±)-**11a**/**12a** was obtained in THF at 60 °C (Table 1, entries 10–11). Gratifyingly, C-alkylation was selective over O-alkylation by using NaH and ^*t*^BuOK in toluene, affording (±)-**11a** in 83% yield (Table 1, entry 12). This result pointed out that NaH could facilitate a fast deprotonation of ^*t*^BuOH formed from the enolization and shift the equilibrium toward its conjugate base. In order to rule out that the possible formation in situ of ^*t*^BuONa in the reaction mixture from ^*t*^BuOK and NaH could be responsible for the improvement of the reaction yield, a control experiment was carried out with just ^*t*^BuONa. A low conversion was observed for the reaction, which was increased after addition of NaH (Table 1, entries 13–14). The use of ^*t*^BuONa/KH gave rise to a similar yield for (±)-**11a** to that observed with the pair ^*t*^BuOK/NaH (Table 1, entry 15). These results support that ^*t*^BuOH generated in the reaction is quenched by a metal hydride. Finally, we also tested a decrease of the amount of ^*t*^BuOK to 0.5 equiv, giving rise to the lowering of the reaction conversion in comparison with using a stoichiometric amount of the base. However, a similar yield was obtained after heating at 60 °C for 1 h (Table 1, entries 16–17 vs entry 12).

**Table 1:**
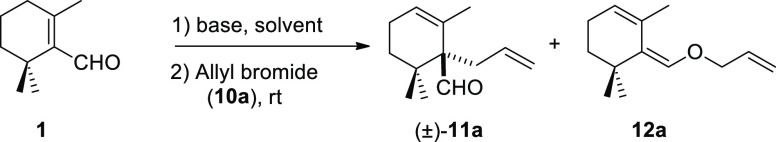
Optimization of Deconjugative α-Alkylation of **1** with Allyl Bromide (**10a**)^a^

entry	base	solvent	time (h)	yield **11a** (%)^b^	yield **12a** (%)^b^
1	^*t*^BuOK	THF	16	8	0
2	^*t*^BuOK	CH_3_CN	16	33	0
3	^*t*^BuOK	toluene	16	43	0
4^c^	^*t*^BuOK	toluene	16	48	0
5^d^	^*t*^BuOK	THF	6	20	56
6^d^	^*t*^BuOK	CH_3_CN	6	30	58
7^d^	^*t*^BuOK	toluene	6	69	26
8	LiHMDS	THF	4	75	15
9^e^	LDA	THF	4	0	0
10^f^	NaH	toluene	16	0	0
11^f^^,^^g^	NaH	THF	6	25	25
12^f^	NaH–^*t*^BuOK	toluene	1	83	0
13	^*t*^BuONa	toluene	15	10	0
14^h^	NaH–^*t*^BuONa	toluene	2	80	0
15^i^	KH–^*t*^BuONa	toluene	3	73	0
16^f^^,^^j^	NaH–^*t*^BuOK	toluene	13	30	0
17^f^^,^^j^^,^^k^	NaH–^*t*^BuOK	toluene	5	78	0

a The reaction was carried out with **1** (1.0 mmol), the base (1.1 mmol), and the solvent (20 mL). After 45 min, allyl bromide (1.5 mmol) was added.

b Isolated yields.

c 3 equiv of ^*t*^BuOK was used.

d 1 equiv of 18-crown-6-ether was used.

e The reaction was carried out at −78 °C and allowed to warm to room temperature.

f 2 equiv of NaH 60% in the oil mineral was used.

g The reaction was carried out at 60 °C.

h Isolated yield after addition of 2 equiv of NaH 60% in the oil mineral.

i 2 equiv of KH 30% in the oil mineral was used.

j 0.5 equiv of ^*t*^BuOK was used.

k Isolated yield after heating at 60 °C.

The experiments shown in Table 1 confirm that the synergistic effect between ^*t*^BuOK/^*t*^BuONa and metal hydrides plays a crucial role in the conversion and selectivity of the deconjugative α-alkylation, influencing the kinetic and increasing the reaction rate. A cooperative interaction between ^*t*^BuOK and strong bases has been previously reported for the synthesis of 1-indanones from β-alkynyl ketones.^14^

With the optimized reaction conditions in hand, the scope and limitations of the deconjugative α-alkylation of β-cyclocitral (**1**) were evaluated with a series of activated alkyl and benzyl halides. In most cases, it undergoes α-alkylation in high to moderate yields under smooth conditions and short reaction times (Table 2). Methyl iodide (**10b**) and benzyl bromide (**10e**) promoted the deconjugative α-alkylation reaction in higher reaction yields (Table 2, entries 1 and 4) in comparison to the yields obtained when allylic or propargylic halides **10c–d** were used (Table 2, entries 2–3). However, the effect of the electron-withdrawing or electron-releasing character of the benzyl group as well as the position of the substituents in the aromatic ring appears to affect the reaction course significantly. Strongly electron-releasing methoxy groups at meta- or para-positions on the aromatic ring and 1,3-benzodioxole groups afforded the corresponding deconjugated aldehydes (±)-**11f** and (±)-**11h–i** in moderate to good yields (Table 2, entries 5–8), whereas *ortho*-methoxy groups furnished the corresponding products (±)-**11j–l** in moderate yields (Table 2, entries 9–11). Furthermore, the use of benzyl bromides **10g** and **10k** gave slightly higher reaction yields than the corresponding benzyl chlorides **10f** and **10j** (Table 2, entry 5 vs entry 6 and entry 9 vs entry 10). Benzyl bromides bearing a bromo substituent at the ortho-position of the aromatic ring afforded the desired deconjugated aldehydes (±)-**11m–n** in good yields (Table 2, entries 12–13). Finally, the reaction was also compatible with other electrophilic groups, such as NO_2_ or CN, on the aromatic ring. Thus, compounds (±)-**11o–p** were obtained in the range of 77–80% yields (Table 2, entries 14–15).

**Table 2:**
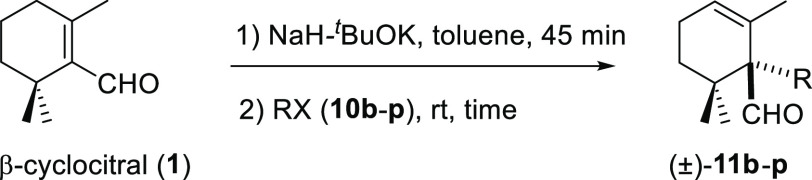

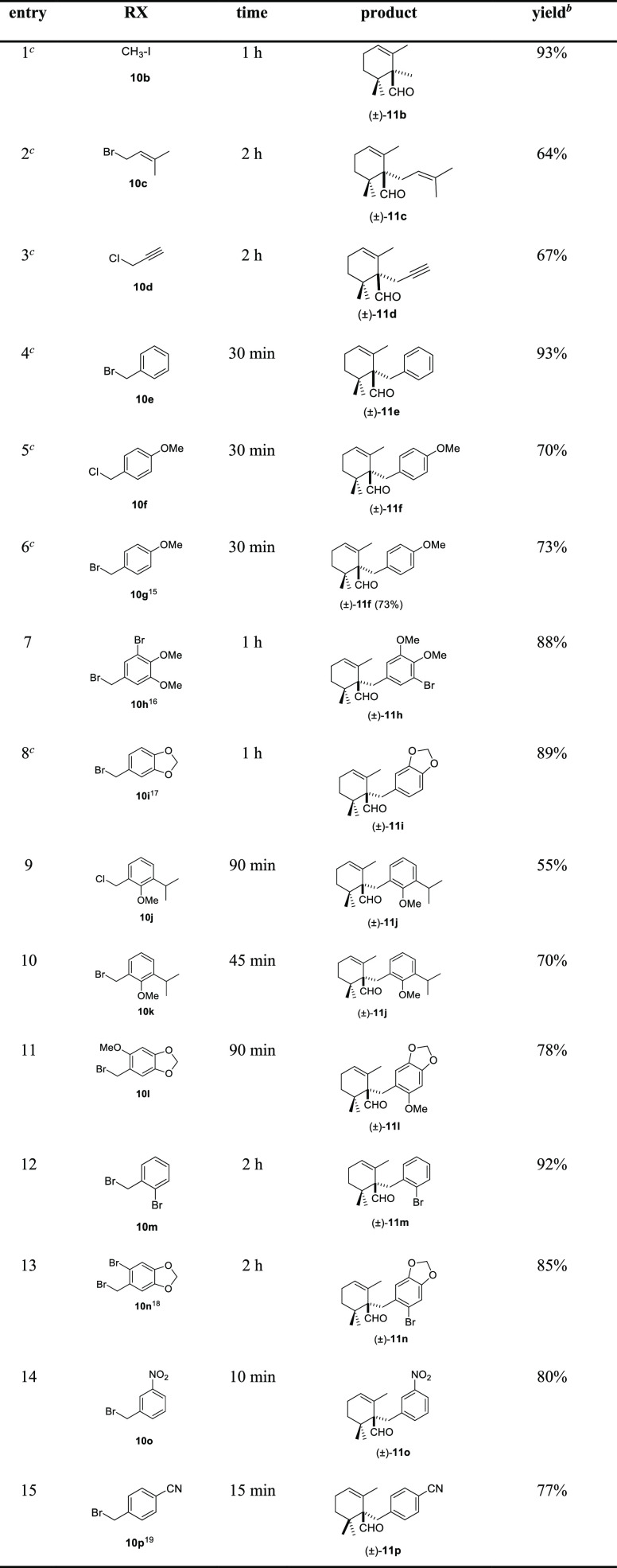
Reaction of β-Cyclocitral (**1**) with Activated Alkyl and Benzyl Halides^a^

a Unless specified, the reaction was carried out with **1** (1.0 mmol), ^*t*^BuOK (1.1 mmol), NaH (2 mmol), and toluene (20 mL). After 45 min, alkyl halide (1.5 mmol) was added.

b Isolated yields.

c Similar yields were obtained using 1 equiv of 18-crown-6 in the absence of NaH.

In order to demonstrate the general applicability of the deconjugative α-alkylation and to explore the further use of this reaction for getting access to other types of terpenoids, the alkylation of other α,β-unsaturated aldehydes with benzyl bromides has also been investigated (Table 3).

**Table 3:**
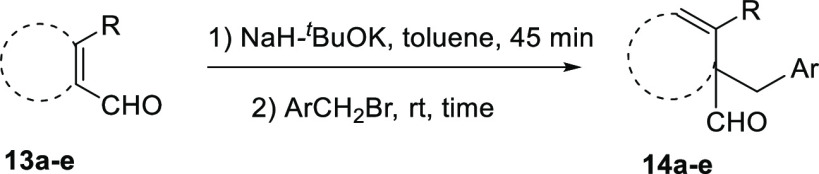

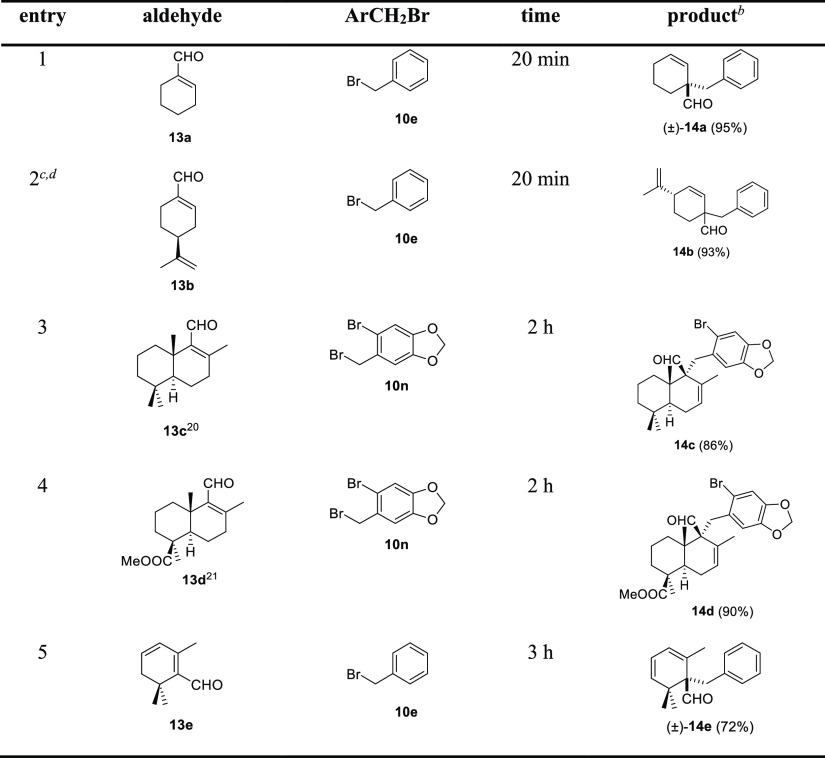
Reaction of α,β-Unsaturated Aldehydes with Benzyl Bromides^a^

a Unless specified, the reaction was carried out with 1.0 mmol of aldehyde, ^*t*^BuOK (1.1 mmol), NaH (2 mmol), and toluene (20 mL). After 45 min, alkyl halide (1.5 mmol) was added.

b Isolated yields.

c Similar yields were obtained using 1 equiv of 18-crown-6 in the absence of NaH.

d An approximate 1:1 epimeric mixture of **14b** was deduced from the ^13^C NMR spectrum.

Cyclohex-1-ene-1-carboxaldehydes **13a** and **13b**, which lack substituents on carbon β, underwent benzylation in high yield in a short reaction time (Table 3, entries 1 and 2). The bicyclic sesquiterpene aldehydes **13c** and **13d**, whose absolute configuration is well known, also underwent this reaction in high yield, affording benzyl derivatives **14c** and **14d**, with complete diastereoselectivity (Table 3, entries 3 and 4). The disposition of the aldehyde group in both products has been confirmed by NOE experiments. The behavior of Δ^8^-drimenals **13c** and **13d** indicates the possibility of using this type of sesquiterpene α,β-unsaturated aldehydes to synthesize a large group of terpenoids such as benzofluorene derivatives by reaction with the appropriate benzyl halide.^21^ Finally, the α,β,γ,δ-unsaturated aldehyde safranal (**13e**) gave the corresponding α-benzylated β,γ,δ,ε-unsaturated aldehyde **14e** in moderate yield (Table 3, entry 5).

The presence of the aldehyde group in these intermediates notably increases the synthetic potential of this new strategy. The formyl group can be removed and allow to introduce functionality in the final compounds. An interesting example of the latter would be the direct transformation of the β,γ-unsaturated aldehyde type **11** into the allyl acetates **15** via a lead(IV) acetate-mediated oxidative fragmentation. LTA has been previously used for the oxidative transformation of homoallyl alcohols to afford allyl acetate derivatives. The reaction proceeded with complete stereoselectivity; the acetyloxy group of the rearranged product and the hydroxymethyl group in the starting material are located at the same face of the molecule.^22^

To expand the scope of Preite’s reaction and illustrate the usefulness of this transformation, some of the synthesized β,γ-unsaturated aldehydes **11** were tested, and the results are shown in Table 4. The treatment of β,γ-unsaturated aldehydes (±)-**11a**, (±)-**11e**, (±)-**11i**, (±)-**11m**, and (±)-**11o** with Pb(OAc)_4_ in refluxing benzene afforded the corresponding allyl acetates (±)-**15a**, (±)-**15e**, (±)-**15i**, (±)-**15m**, and (±)-**15o**, respectively, in high yields and short reaction times (Table 4). With the aim of confirming the stereoselectivity previously described for the similar oxidative cleavage of homoallyl alcohols, we carried out the reaction with compound **14c**. Unfortunately, it failed and produced a complex mixture. However, our results are in agreement with the concerted mechanistic pathway proposed by Preite et al. where a syn stereoselectivity was observed, with the hydroxymethyl group of the starting material and the acetyloxy group of rearranged compounds located at the same side of the molecule.^22^

**Table 4:**
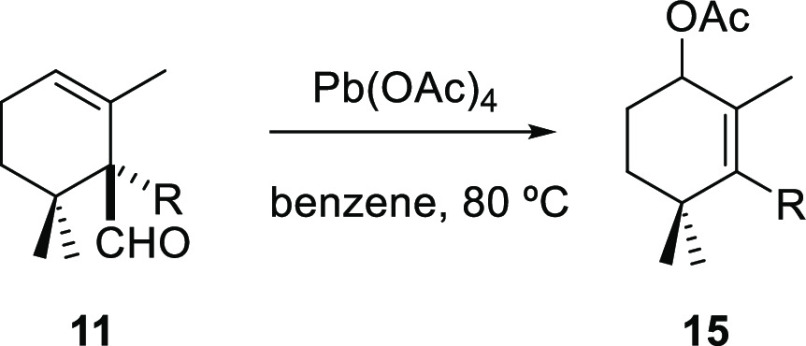

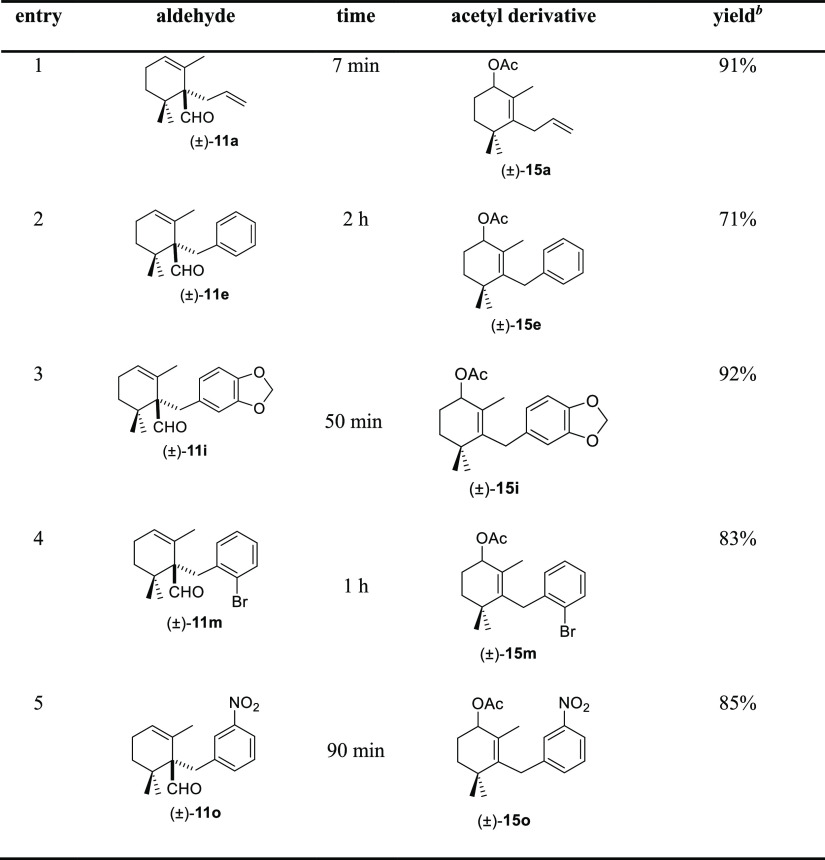
Transformation of β,γ-Unsaturated Aldehydes **11** into Allyl Acetates **15**^a^

a Unless specified, the reaction was carried out with β,γ-unsaturated aldehyde (1 mmol), Pb(OAc)_4_ (1.1 mmol), and benzene (7 mL) at 80 °C.

b Isolated yields.

**Scheme 4:**
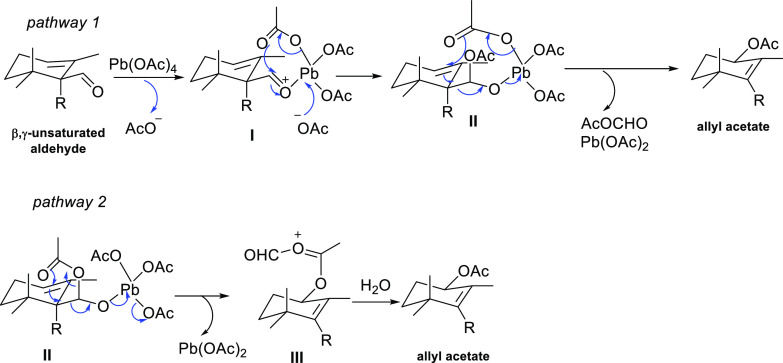
Tentative Mechanisms for the LTA-Mediated Transformation of β,γ-Unsaturated Aldehyde into Allyl Acetates

Based on our experimental results and the literature precsedents,^22,23^Scheme 4 shows two tentative mechanistic pathways for the direct oxidative fragmentation of β,γ-unsaturated aldehydes. In a first step, one of the acetate groups of Pb(OAc)_4_ would be exchanged by the oxygen atom of the β,γ-unsaturated aldehyde to give intermediate **I**. Then, the released acetate ion could attack again the more accessible Pb(IV), with the simultaneous intramolecular nucleophilic addition of another Pb(IV) acetate group to the complexed aldehyde to afford intermediate **II**. The attack of one of the acetate groups to the olefin through a nine-membered cyclic transition state would generate the allyl acetate with the simultaneous removal of Pb(OAc)_2_ and mixed anhydride (pathway 1). However, a seven-membered cyclic transition state cannot be ruled out to give rise to intermediate **III**, which would be hydrolyzed to generate the allyl acetate (pathway 2).

## Conclusions

In summary, a new alternative strategy for synthesizing a wide variety of terpenoid precursors is reported. This approach is based on the reaction of an electrophilic activated alkyl or benzyl halide with a nucleophilic terpenic α,β-unsaturated aldehyde. ^*t*^BuOK and NaH interact synergistically, enhancing notably the kinetics and the selectivity for the C-alkylated derivatives. The β,γ-unsaturated aldehydes resulting from this alkylation undergo a lead(IV) acetate-mediated oxidative fragmentation, affording in high yield the corresponding allyl acetates. The deconjugative α-alkylation of α,β-unsaturated aldehydes and deformylation reaction described in this paper could serve as a platform to access in an efficient and simple manner to a wide variety of terpenoid skeletons.

## Experimental Section

### General Procedures

Aldehydes **1**, **13a**, **13b**, and **13e** were obtained from commercial suppliers and used without further purification. Unless stated otherwise, reactions were performed in oven-dried glassware under an argon atmosphere using dry solvents. Solvents were dried as follows: THF, diethyl ether (Et_2_O), and toluene over Na-benzophenone; dichloromethane (DCM) over CaH_2_; and acetonitrile over molecular sieves 4 Å. An oil bath was used as the heating source for the reactions that require heating. Thin-layer chromatography (TLC) was performed using F254 precoated plates (0.25 mm) and visualized by UV fluorescence quenching and phosphomolybdic acid solution in ethanol staining. Flash chromatography was performed on silica gel (230–400 mesh). Chromatography separations were carried out using a conventional column on silica gel 60 (230–400 mesh) using hexanes–AcOEt (AcOEt–hexane) or diethyl ether–hexane (ether–hexane) mixtures of increasing polarity. ^1^H and ^13^C{^1^H} NMR spectra were recorded at 600, 500, and 400 MHz and at 150, 125, and 100 MHz, respectively. CDCl_3_ was treated with K_2_CO_3_. Chemical shifts (δ H) are quoted in parts per million (ppm) referenced to the appropriate residual solvent peak and tetramethylsilane. Data for ^1^H NMR spectra are reported as follows: chemical shift (δ ppm) [multiplicity, coupling constant (Hz), and integration], with the abbreviations s, br s, d, br d, t, dt, dq, sept, and m denoting the singlet, broad singlet, doublet, broad doublet, triplet, doublet triplet, doublet quartet, septet, and multiplet, respectively. *J* = coupling constant in hertz (Hz). Data for ^13^C{^1^H} NMR spectra are reported in terms of chemical shift relative to Me_4_Si (δ 0.0), and the signals were assigned utilizing DEPT experiments and on the basis of heteronuclear correlations. Infrared (IR) spectra were recorded as thin films or as solids on an FTIR spectrophotometer with samples between sodium chloride plates or as potassium bromide pellets and are reported in frequency of absorption (cm^–1^). Only selected absorbances (ν_max_) are reported. ([α]_D_) measurements were carried out in a polarimeter utilizing a 1 dm length cell and CHCl_3_ as a solvent. Concentration is expressed in mg/mL. High-resolution mass spectra were recorded on a spectrometer utilizing a Q-TOF analyzer and ESI^+^ ionization.

### General Procedure for the Preparation of Alkyl Bromides **10g–i**, **10n**, and **10p**

To a solution of the corresponding commercially available alcohol (1 mmol) in diethyl ether (10 mL) was added PBr_3_ (1 mmol) at 0 °C, and the mixture was stirred at this temperature, being monitorized by TLC. Then, it was quenched with water (3 mL) and diluted with Et_2_O (20 mL). The organic layer was washed with water (3 × 10 mL), dried with anhydrous Na_2_SO_4_, and filtered, and the solvent was evaporated under reduced pressure to give the corresponding alkyl bromides **10g–i**, **10n**, and **10p**.

#### 1-(Bromomethyl)-4-methoxybenzene (**10g**)

Yellow oil [yield 537 mg (89%) from 414.6 mg (3 mmol) of (4-methoxyphenyl)methanol]. The spectroscopic data were in agreement with those described in the literature.^15^

#### 1-Bromo-5-(bromomethyl)-2,3-dimethoxybenzene (**10h**)

White solid [yield 790 mg (85%) from 740.8 mg (3 mmol) of (3-bromo-4,5-dimethoxyphenyl)methanol]. The spectroscopic data were in agreement with those described in the literature.^16^

#### 5-(Bromomethyl)benzo[*d*][1,3]dioxole (**10i**)

White solid [yield 619 mg (96%) from 456.2 mg (3 mmol) of benzo[*d*][1,3]dioxol-5-ylmethanol]. The spectroscopic data were in agreement with those described in the literature.^17^

#### 5-Bromo-6-(bromomethyl)benzo[*d*][1,3]dioxole (**10n**)

White solid [yield 776 mg (88%) from 693.1 mg (3 mmol) of (6-bromobenzo[*d*][1,3]dioxol-5-yl)methanol]. The spectroscopic data were in agreement with those described in the literature.^18^

#### 4-(Bromomethyl)benzonitrile (**10p**)

White solid [yield 531 mg (82%) from 439.8 mg (3 mmol) of 4-(hydroxymethyl)benzonitrile]. The spectroscopic data were in agreement with those described in the literature.^19^

### Preparation of 1-(Chloromethyl)-3-isopropyl-2-methoxybenzene (**10j**)

A round-bottom flask charged with 2-isopropylphenol (10 g, 73 mmol) and *p*-formaldehyde (4.4 g, 88.8 mmol) in dry DCM (150 mL) under an argon atmosphere was cooled at −30 °C. Then, Et_2_AlCl (25% wt solution in toluene, 47.6 mL, 87.6 mmol) was added dropwise, and the reaction mixture was allowed to warm to room temperature. The reaction mixture was stirred for 3 h and was quenched with water (50 mL). DCM was evaporated under reduced pressure, and the aqueous layer was extracted with ethyl acetate (3 × 100 mL). The combined organic solution was dried over anhydrous sodium sulfate and filtered. The solvent was evaporated under reduced pressure to give a crude product which was purified by silica gel column chromatography (20% AcOEt/hexane), affording 2-(hydroxymethyl)-6-isopropylphenol. Colorless oil (10.97 g, 90%). ^1^H NMR (400 MHz, CD_3_COCD_3_): δ 8.46 (br s, O*H*), 7.14 (dd, *J* = 7.6, 1.7 Hz, 1H), 6.91 (m, 1H), 6.80 (t, *J* = 7.6 Hz, 1H), 5.23 (br s, O*H*), 4.87 (s, 2H), 3.40 (sept, *J* = 6.9 Hz, 1H), 1.26 (d, *J* = 6.9 Hz, 6H). ^13^C{^1^H} NMR (100 MHz, CD_3_COCD_3_): δ 154.5 (C), 135.9 (C), 125.9 (CH), 125.8 (C), 125.5 (CH), 120.0 (CH), 64.4 (CH_2_), 27.1 (CH), 22.9 (CH_3_). IR (film): 3053, 2965, 1456, 1421, 1264, 733, 704 cm^–1^. HRMS (ESI/TOF) *m*/*z*: [M + Na]^+^ calcd for C_10_H_14_O_2_Na, 189.0891; found, 189.0887.

K_2_CO_3_ (13.65 g, 99 mmol) was added at 0 °C to a stirred solution of 2-(hydroxymethyl)-6-isopropylphenol (10.97 g, 66 mmol) in acetone (120 mL). After 15 min, Me_2_SO_4_ (6.3 mL, 66 mmol) was added and refluxed overnight. Then, water was added (50 mL) and the solvent was evaporated under reduced pressure. The aqueous layer was extracted with two portions of diethyl ether (2 × 80 mL), dried over anhydrous sodium sulfate, and filtered. The solvent was evaporated under reduced pressure to give a crude product which was purified by silica gel column chromatography (40% Et_2_O/hexane), affording (3-isopropyl-2-methoxyphenyl)methanol. Colorless oil (8.18 g, 68.9%). ^1^H NMR (500 MHz, CD_3_COCD_3_): δ 7.32 (m, 1H), 7.22 (dd, *J* = 7.6, 1.7 Hz, 1H), 7.10 (t, *J* = 7.6 Hz, 1H), 4.71 (d, *J* = 5.7 Hz, 2H), 4.12 (t, *J* = 5.7 Hz, O*H*), 3.76 (s, 3H), 3.36 (sept, *J* = 7.0 Hz, 1H), 1.23 (d, *J* = 7.0 Hz, 6H). ^13^C{^1^H} NMR (125 MHz, CD_3_COCD_3_): δ 155.7 (C), 142.0 (C), 135.9 (C), 126.9 (CH), 126.1 (CH), 125.0 (CH), 62.2 (CH_2_), 59.9 (CH_3_), 26.7 (CH), 24.1 (CH_3_). IR (film): 3053, 2966, 1451, 1428, 1264, 1206, 1096, 733, 703 cm^–1^. HRMS (ESI/TOF) *m*/*z*: [M + Na]^+^ calcd for C_11_H_16_O_2_Na, 203.1048; found, 203.1048.

Thionyl chloride (1.94 mL, 27 mmol) was slowly added to a solution of (3-isopropyl-2-methoxyphenyl)methanol (3.2 g, 18 mmol) and pyridine (1 drop) in dry CH_2_Cl_2_ (75 mL) at 0 °C. The reaction was allowed to warm to room temperature for 5 h and quenched with water (20 mL). The aqueous phase was extracted with DCM (3 × 50 mL), and the combined organic solutions were washed with brine (80 mL), dried over anhydrous sodium sulfate, and filtered. Evaporation of the solvent under reduced pressure yielded 1-(chloromethyl)-3-isopropyl-2-methoxybenzene (**10j**), which was used without chromatography purification. Colorless oil (2.3 g, 65.0%). ^1^H NMR (400 MHz, CDCl_3_): δ 7.33 (m, 2H), 7.19 (t, *J* = 7.6 Hz, 1H), 4.75 (s, 2H), 3.93 (s, 3H), 3.43 (sept, *J* = 6.9 Hz, 1H), 1.32 (d, *J* = 6.9 Hz, 6H). ^13^C{^1^H} NMR (125 MHz, CDCl_3_): δ 155.5 (C), 142.2 (C), 130.7 (C), 128.4 (CH), 127.4 (CH), 124.7 (CH), 62.5 (CH_3_), 41.3 (CH_2_), 26.2 (CH), 23.8 (CH_3_). IR (film): 2972, 1467, 1429, 1264, 1208, 1094, 1049, 1006, 799, 734, 703 cm^–1^. HRMS (ESI/TOF) *m*/*z*: [M–Cl]^+^ calcd for C_11_H_15_O, 163.1123; found, 163.1127.

### Preparation of 1-(Bromomethyl)-3-isopropyl-2-methoxybenzene (**10k**)

The general procedure for the preparation of alkyl bromides was followed using (3-isopropyl-2-methoxyphenyl)methanol (3.8 g, 21.1 mmol) to afford 1-(bromomethyl)-3-isopropyl-2-methoxybenzene (**10k**), which was further used without chromatography purification. Yellow oil (4.3 g, 83.7%). ^1^H NMR (400 MHz, CD_3_COCD_3_): δ 7.29 (m, 2H), 7.10 (t, *J* = 7.7 Hz, 1H), 4.66 (s, 2H), 3.86 (s, 3H), 3.34 (sept, *J* = 6.7 Hz, 1H), 1.22 (d, *J* = 6.7 Hz, 6H). ^13^C{^1^H} NMR (125 MHz, CD_3_COCD_3_): δ 156.6 (C), 143.0 (C), 132.1 (C), 129.9 (CH), 128.3 (CH), 125.5 (CH), 62.5 (CH_3_), 29.4 (CH_2_), 26.9 (CH), 24.1 (CH_3_). IR (film): 2961, 2861, 2830, 1463, 1428, 1383, 1256, 1222, 1203, 1168, 1004, 795, 763 cm^–1^. HRMS (ESI/TOF) *m*/*z*: [M + H–HBr]^+^ calcd for C_11_H_15_O, 163.1123; found, 163.1126.

### Preparation of 5-(Bromomethyl)-6-methoxybenzo[*d*][1,3]dioxole (**10l**)

To sesamol (5 g, 36 mmol) in water (110 mL) at 0 °C were added formaldehyde (37% wt in water, 5.5 mL, 72 mmol) and calcium oxide (1.02 g, 18 mmol). After 1 h, saturated aqueous ammonium chloride was added and the aqueous layer was extracted with ether (3 × 150 mL), dried over anhydrous Na_2_SO_4_, concentrated, and purified by column chromatography on silica gel (30% AcOEt/hexane) to give 6-(hydroxymethyl)benzo[*d*][1,3]dioxol-5-ol. Red solid (5.3 g, 82%), mp 183–185 °C. ^1^H NMR (500 MHz, CD_3_COCD_3_): δ 6.74 (s, 1H), 6.39 (s, 1H), 5.86 (s, 2H), 4.62 (s, 2H). ^13^C{^1^H} NMR (125 MHz, CD_3_COCD_3_): δ 151.2 (C), 148.2 (C), 141.6 (C), 120.5 (C), 108.5 (CH), 102.0 (CH), 98.9 (CH_2_), 61.9 (CH_2_). IR (film): 3500 (br s), 2922, 2853, 1503, 1484, 1190, 1157, 1039, 937, 852, 821 cm^–1^. HRMS (ESI/TOF) *m*/*z*: [M + H–H_2_O]^+^ calcd for C_8_H_7_O_3_, 151.0395; found, 151.0399.

6-(Hydroxymethyl)benzo[*d*][1,3]dioxol-5-ol (5.7 g, 34 mmol) was dissolved in acetone (60 mL), and K_2_CO_3_ (7.02 g, 50.9 mmol) was added and stirred at 0 °C for 15 min. Then, (CH_3_)_2_SO_4_ (3.2 mL, 34 mmol) was added and refluxed overnight. The solvent was evaporated, and water (40 mL) was added and extracted with diethyl ether (3 × 50 mL). The organic layer was dried over anhydrous Na_2_SO_4_, concentrated, and purified by flash chromatography on silica gel (30% Et_2_O/hexane) to give (6-methoxybenzo[*d*][1,3]dioxol-5-yl)methanol (4.3 g, 64.5%), which was used immediately. Spectroscopic data were consistent with those described in the literature.^24^

Finally, the general procedure for the preparation of alkyl bromides was followed to give 5-(bromomethyl)-6-methoxybenzo[*d*][1,3]dioxole (**10l**). Amorphous yellow solid (4.1 g, 76.8%). ^1^H NMR (500 MHz, CD_3_COCD_3_): δ 6.90 (s, 1H), 6.69 (s, 1H), 5.96 (s, 2H), 4.60 (s, 2H), 3.85 (s, 3H). ^13^C{^1^H} NMR (125 MHz, CD_3_COCD_3_): δ 154.8 (C), 150.4 (C), 142.2 (C), 119.3 (C), 111.1 (CH), 102.8 (CH), 96.1 (CH_2_), 57.3 (CH_2_), 31.2 (CH_2_). IR (film): 3018, 1504, 1466, 1215, 1040, 745 cm^–1^. HRMS (ESI/TOF) *m*/*z*: [M + H–HBr]^+^ calcd for C_9_H_9_O_3_, 165.0552; found, 165.0547.

### General Procedure for the Deconjugative α-Alkylation of α,β-Unsaturated Aldehydes with Alkyl Halides

To a solution of unsaturated aldehydes **1** or **13a–e** (1.0 mmol) in anhydrous toluene (15 mL) were added successively sodium hydride (2 mmol, 60% dispersion in mineral oil) and potassium *tert*-butoxide (1.1 mmol), and the mixture was stirred for 45 min at room temperature. Then, a solution of the corresponding alkyl halide (1.5 mmol) in toluene (5 mL) was added; the mixture was stirred under an inert atmosphere for the specified time, and the course of the reaction was monitored by TLC. When the starting material was consumed, water (10 mL) was carefully added and the aqueous layer was extracted with two portions of ethyl acetate (2 × 20 mL). The combined organic solution was dried over anhydrous Na_2_SO_4_ and filtered, and the solvent was evaporated under reduced pressure to give a crude, which was purified by silica gel column chromatography. Elution with petroleum ether/ethyl acetate mixtures yielded compounds (±)-**11a–f**, (±)-**11h–j**, (±)-**11l–p**, **12a**, and (±)-**14a–e** in the yields indicated in Tables 1–3.
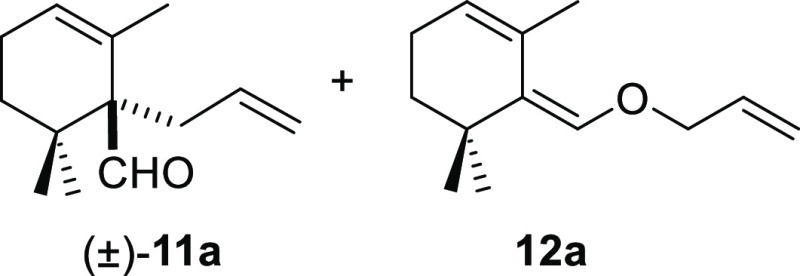


#### (±)-1-Allyl-2,6,6-trimethylcyclohex-2-ene-1-carbaldehyde ((±)-**11a**) and (*Z*)-6-((Allyloxy)methylene)-1,5,5-trimethylcyclohex-1-ene (**12a**)

To a solution of β-cyclocitral (**1**) (200 mg, 1.31 mmol) in toluene (25 mL), NaH (105 mg, 2.62 mmol), ^*t*^BuOK (162 mg, 1.45 mmol), and allyl bromide (**10a**) (236 mg, 1.96 mmol) were added and stirred for 1 h. Following the same workup used in the general procedure and after column chromatography, using 2% EtOAc/hexane, compound **11a** was obtained as a colorless oil (209 mg, 83%). ((±)-**11a**): ^1^H NMR (500 MHz, CDCl_3_): δ 9.64 (s, 1H), 5.85 (br s, 1H), 5.80 (m, 1H), 5.09 (d, *J* = 17.2 Hz, 1H), 4.98 (d, *J* = 9.9 Hz, 1H), 2.54 (m, 2H), 2.11 (br s, 2H), 1.61 (s, 3H), 1.56 (dt, *J* = 13.5, 6.7 Hz, 1H), 1.46 (dt, *J* = 13.5, 6.7 Hz, 1H), 1.03 (s, 3H), 0.96 (s, 3H). ^13^C{^1^H} NMR (125 MHz, CDCl_3_): δ 204.6 (CH), 136.9 (CH), 130.1 (C), 127.9 (CH), 115.8 (CH_2_), 59.2 (C), 35.7 (C), 33.8 (CH_2_), 33.3 (CH_2_), 25.3 (CH_3_), 25.1 (CH_3_), 22.7 (CH_2_), 20.7 (CH_3_). IR (film): 2916, 1717, 1674, 1447, 1378, 1225, 1051, 1025, 809 cm^–1^. HRMS (ESI/TOF) *m*/*z*: [M + H]^+^ calcd for C_13_H_21_O, 193.1592; found, 193.1595. (**12a**) (see Table 1, entries 4–7 and 10): ^1^H NMR (400 MHz, CDCl_3_): δ 6.07 (s, 1H), 5.93 (ddt, *J* = 17.2, 10.5, 5.2 Hz, 1H), 5.44 (m, 1H), 5.32 (dq, *J* = 17.2, 1.7 Hz, 1H), 5.20 (dq, *J* = 10.5, 1.7 Hz, 1H), 4.27 (dt, *J* = 5.2, 1.7 Hz, 2H), 2.05 (m, 2H), 1.72 (q, *J* = 1.7 Hz, 3H), 1.41 (t, *J* = 6.2 Hz, 2H), 1.21 (s, 6H). ^13^C{^1^H} NMR (100 MHz, CDCl_3_): δ 142.7 (CH), 134.1 (C), 130.6 (C), 124.0 (CH), 123.6 (CH), 116.9 (CH_2_), 73.2 (CH_2_), 39.2 (CH_2_), 33.2 (C), 27.3 (2× CH_3_), 22.8 (CH_2_), 20.6 (CH_3_). IR (film): 2927, 1676, 1455, 1127, 929 cm^–1^. HRMS (ESI/TOF) *m*/*z*: [M + H]^+^ calcd for C_13_H_21_O, 193.1592; found,193.1594.
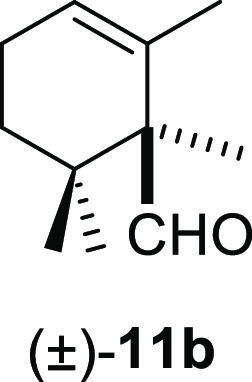


#### (±)-1,2,6,6-Tetramethylcyclohex-2-ene-1-carbaldehyde ((±)-**11b**)

To a solution of β-cyclocitral (**1**) (145 mg, 0.95 mmol) in toluene (15 mL), NaH (76 mg, 1.9 mmol), ^*t*^BuOK (123 mg, 1.1 mmol), and iodomethane (220 mg, 1.55 mmol) were added and stirred for 1 h. Following the same workup used in the general procedure and after column chromatography, using 5% EtOAc/hexane, compound **11b** was obtained as a colorless oil (147 mg, 93%). ^1^H NMR (500 MHz, CDCl_3_): δ 9.60 (s, 1H), 5.74 (br s, 1H), 2.10 (m, 2H), 1.81 (m, 1H), 1.51 (br s, 3H), 1.38 (dt, *J* = 12.8, 3.3 Hz, 1H), 1.07 (s, 3H), 0.91 (s, 3H), 0.85 (s, 3H). ^13^C{^1^H} NMR (125 MHz, CDCl_3_): δ 203.6 (CH), 131.1 (C), 127.0 (CH), 56.5 (C), 34.4 (C), 33.1 (CH_2_), 24.9 (CH_3_), 24.2 (CH_3_), 22.7 (CH_2_), 19.9 (CH_3_), 12.6 (CH_3_). IR (film): 2963, 2874, 1703, 1634, 1366, 1309, 1233, 1180, 1122, 1079, 1032 cm^–1^. HRMS (ESI/TOF) *m*/*z*: [M + H]^+^ calcd for C_11_H_19_O, 167.1436; found,167.1443.
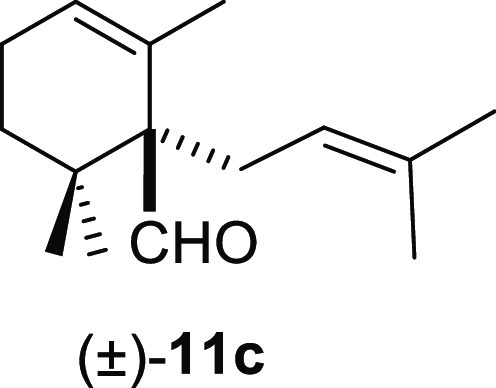


#### (±)-2,6,6-Trimethyl-1-(3-methylbut-2-en-yl)cyclohex-2-ene-1-carbaldehyde ((±)-**11c**)

To a solution of β-cyclocitral (**1**) (178 mg, 1.17 mmol) in toluene (20 mL), NaH (94 mg, 2.34 mmol), ^*t*^BuOK (154 mg, 1.3 mmol), and **10c** (261 mg, 1.75 mmol) were added and stirred for 2 h. Following the same workup used in the general procedure and after column chromatography, using 2% EtOAc/hexane, compound **11c** was obtained as a colorless oil (239 mg, 64%). ^1^H NMR (500 MHz, CDCl_3_): δ 9.61 (s, 1H), 5.82 (br s, 1H), 5.06 (m, 1H), 2.42 (m, 2H), 2.08 (m, 2H), 1.65 (d, *J* = 1.5 Hz, 3H), 1.62 (d, *J* = 1.5 Hz, 3H), 1.56 (q, *J* = 1.8 Hz, 3H), 1.48 (t, *J* = 6.8 Hz, 2H), 0.98 (s, 3H), 0.88 (s, 3H). ^13^C{^1^H} NMR (125 MHz, CDCl_3_): δ 205.0 (CH), 130.9 (C), 130.6 (C), 127.9 (CH), 122.2 (CH), 59.3 (C), 35.5 (C), 33.8 (CH_2_), 26.9 (CH_2_), 25.9 (CH_3_), 25.4 (CH_3_), 25.3 (CH_3_), 22.7 (CH_2_), 20.7 (CH_3_), 18.0 (CH_3_). IR (film): 2923, 1715, 1676, 1453, 1381, 1365, 1138, 1035, 754 cm^–1^. HRMS (ESI/TOF) *m*/*z*: [M + H]^+^ calcd for C_15_H_25_O, 221.1905; found, 221.1909.
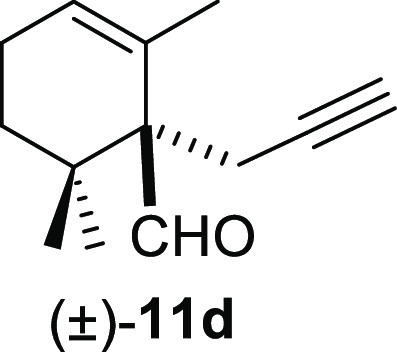


#### (±)-2,6,6-Trimethyl-1-(prop-2-yn-1-yl)cyclohex-2-ene-1-carbaldehyde ((±)-**11d**)

To a solution of β-cyclocitral (**1**) (230 mg, 1.51 mmol) in toluene (25 mL), NaH (121 mg, 3.02 mmol), ^*t*^BuOK (186 mg, 1.66 mmol), and propargyl chloride **10d** (169 mg, 2.27 mmol) were added and stirred for 2 h. Following the same workup used in the general procedure and after column chromatography, using 5% EtOAc/hexane, compound **11d** was obtained as a colorless oil (192 mg, 67%). ^1^H NMR (500 MHz, CDCl_3_): δ 9.57 (s, 1H), 5.90 (br s, 1H), 2.75 (dd, *J* = 17.8, 2.7 Hz, 1H), 2.48 (dd, *J* = 17.8, 2.7 Hz, 1H), 2.12 (m, 2H), 1.95 (t, *J* = 2.7 Hz, 1H), 1.64 (s, 3H), 1.76–1.52 (m, 2H), 1.06 (s, 3H), 1.00 (s, 3H). ^13^C{^1^H} NMR (125 MHz, CDCl_3_): δ 201.8 (CH), 129.0 (CH), 125.4 (C), 83.4 (C), 70.6 (CH), 63.7 (C), 59.2 (CH_2_), 35.4 (C), 33.8 (CH_2_), 25.4 (CH_3_), 22.6 (CH_2_), 20.1 (CH_3_), 17.4 (CH_3_). IR (film): 3324, 2919, 2126, 1715, 1673, 1448, 1374, 1224, 1053, 1032 cm^–1^. HRMS (ESI/TOF) *m*/*z*: [M + Na]^+^ calcd for C_13_H_18_ONa, 213.1255; found, 213.1249.
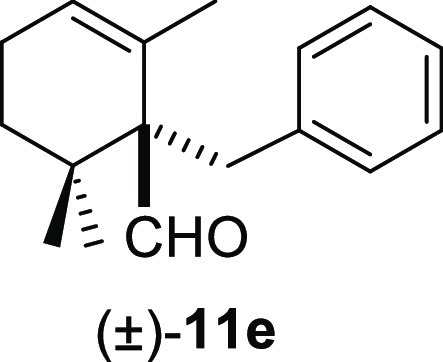


#### (±)-1-Benzyl-2,6,6-trimethylcyclohex-2-ene-1-carbaldehyde ((±)-**11e**)

To a solution of β-cyclocitral (**1**) (210 mg, 1.38 mmol) in toluene (25 mL), NaH (110 mg, 2.76 mmol), ^*t*^BuOK (170 mg, 1.52 mmol), and benzyl bromide (**10e**) (354 mg, 2.07 mmol) were added and stirred for 30 min. Following the same workup used in the general procedure and after column chromatography, using 3% EtOAc/hexane, compound **11e** was obtained as a colorless oil (310 mg, 93%). ^1^H NMR (400 MHz, CDCl_3_): δ 9.79 (s, 1H), 7.25–7.20 (m, 5H), 5.80 (br s, 1H), 3.47 (d, *J* = 13.4 Hz, 1H), 2.75 (d, *J* = 13.4 Hz, 1H), 2.25–2.06 (m, 2H), 1.65 (ddd, *J* = 13.8, 10.5, 7.0 Hz, 1H), 1.24 (s, 3H), 1.21 (m, 1H), 1.09 (s, 3H), 1.04 (s, 3H). ^13^C{^1^H} NMR (100 MHz, CDCl_3_): δ 206.4 (CH), 139.5 (C), 131.2 (C), 130.7 (2× CH), 127.9 (2× CH), 126.6 (CH), 126.0 (CH), 61.0 (C), 36.7 (C), 36.2 (CH_2_), 32.7 (CH_2_), 25.3 (CH_3_), 24.0 (CH_3_), 22.9 (CH_2_), 21.9 (CH_3_). IR (film): 2930, 2860, 1727, 1529, 1470, 1369, 1347, 1241, 1017, 997, 962, 802, 755, 725, 672 cm^–1^. HRMS (ESI/TOF) *m*/*z*: [M–H_2_ + H]^+^ calcd for C_17_H_21_O, 241.1592; found, 241.1597.
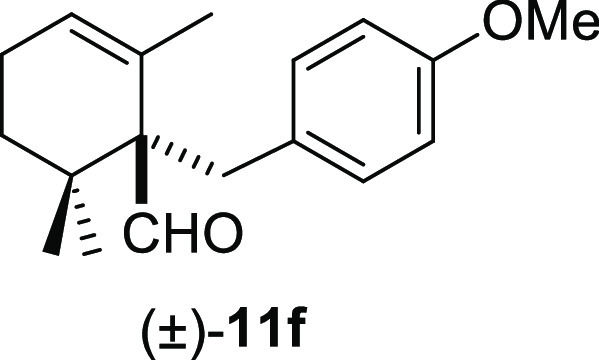


#### (±)-1-(4-Methoxybenzyl)-2,6,6-trimethylcyclohex-2-ene-1-carbaldehyde ((±)-**11f**)

To a solution of β-cyclocitral (**1**) (250 mg, 1.64 mmol) in toluene (25 mL), NaH (131 mg, 3.28 mmol), ^*t*^BuOK (362 mg, 1.80 mmol), and **10g** (385 mg, 1.91 mmol) were added and stirred for 30 min. Following the same workup used in the general procedure and after column chromatography, using 5% EtOAc/hexane, compound **11f** was obtained as a colorless syrup (326 mg, 73%). ^1^H NMR (500 MHz, CDCl_3_): δ 9.71 (s, 1H), 7.07 (d, *J* = 8.4 Hz, 2H), 6.73 (d, *J* = 8.4 Hz, 2H), 5.73 (br s, 1H), 3.75 (s, 3H), 3.31 (d, *J* = 13.1 Hz, 1H), 2.64 (d, *J* = 13.1 Hz, 1H), 2.06 (m, 2H), 1.56 (m, 1H), 1.20 (s, 3H), 1.12 (dd, *J* = 1.8, 6.6 Hz, 1H), 1.01 (s, 3H), 0.95 (s, 3H). ^13^C{^1^H} NMR (125 MHz, CDCl_3_): δ 206.4 (C), 157.9 (C), 131.5 (2× CH), 131.4 (C), 131.3 (C), 126.4 (CH), 113.3 (2× CH), 60.9 (C), 55.1 (CH_3_), 36.6 (C), 35.3 (CH_2_), 32.7 (CH_2_), 25.2 (CH_2_), 24.0 (CH_3_), 22.9 (CH_3_), 21.9 (CH_3_). IR (film): 2932, 1715, 1610, 1510, 1462, 1245, 1177, 1034, 821 cm^–1^. HRMS (ESI/TOF) *m*/*z*: [M + H]^+^ calcd for C_18_H_25_O_2_, 273.1855; found, 273.1846.
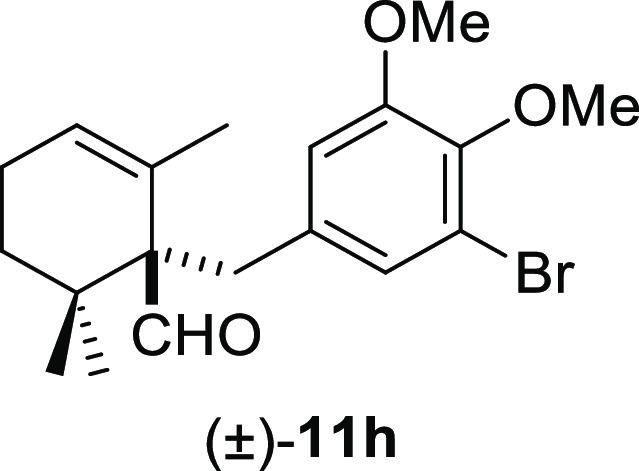


#### (±)-1-(3-Bromo-4,5-dimethoxybenzyl)-2,6,6-trimethylcyclohex-2-enecarbaldehyde ((±)-**11h**)

To a solution of β-cyclocitral (**1**) (177 mg, 1.16 mmol) in toluene (15 mL), NaH (93 mg, 2.32 mmol), ^*t*^BuOK (143 mg, 1.28 mmol), and **10h** (540 mg, 1.74 mmol) were added and stirred for 1 h. Following the same workup used in the general procedure and after column chromatography, using 7% EtOAc/hexane, compound **11h** was obtained as a colorless syrup (389 mg, 88%). ^1^H NMR (500 MHz, CDCl_3_): δ 9.67 (s, 1H), 6.92 (d, *J* = 1.9 Hz, 1H), 6.72 (d, *J* = 1.9 Hz, 1H), 5.76 (br s, 1H), 3.78 (s, 6H), 3.29 (d, *J* = 13.4 Hz, 1H), 2.53 (d, *J* = 13.4 Hz, 1H), 2.12 (m, 2H), 1.57 (ddd, *J* = 13.8, 10.4, 7.0 Hz, 1H), 1.20 (s, 3H), 1.19–1.10 (m, 1H), 1.02 (s, 3H), 0.92 (s, 3H). ^13^C{^1^H} NMR (125 MHz, CDCl_3_): δ 205.9 (CH), 153.0 (C), 144.8 (C), 136.7 (C), 131.0 (C), 126.6 (2× CH), 116.8 (C), 114.3 (CH), 61.0 (CH_3_), 60.6 (CH_3_), 56.0 (C), 36.7 (C), 35.7 (CH_2_), 32.6 (CH_2_), 25.2 (CH_3_), 23.8 (CH_3_), 22.8 (CH_2_), 22.0 (CH_3_). IR (film): 2947, 1715, 1595, 1565, 1489, 1463, 1429, 1414, 1313, 1277, 1214, 1184, 1142, 1047, 1001, 879 cm^–1^. HRMS (ESI/TOF) *m*/*z*: [M + H]^+^ calcd for C_19_H_26_O_3_Br, 381.1065; found, 381.1056.
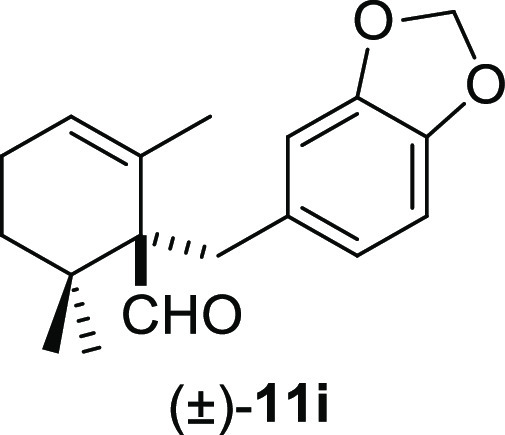


#### (±)-1-(Benzo[*d*][1,3]dioxol-5-ylmethyl)-2,6,6-trimethylcyclohex-2-ene-1-carbaldehyde ((±)-**11i**)

To a solution of β-cyclocitral (**1**) (300 mg, 1.97 mmol) in toluene (25 mL), NaH (158 mg, 3.94 mmol), ^*t*^BuOK (244 mg, 2.18 mmol), and **10i** (634 mg, 2.95 mmol) were added and stirred for 1 h. Following the same workup used in the general procedure and after column chromatography, using 5% EtOAc/hexane, compound **11i** was obtained as a white solid (501 mg, 89%). ^1^H NMR (500 MHz, CDCl_3_): δ 9.73 (s, 1H), 6.70 (br s, 1H), 6.67 (d, *J* = 8.0 Hz, 1H), 6.65 (d, *J* = 8.0 Hz, 1H), 5.89 (s, 2H), 5.76 (br s, 1H), 3.34 (d, *J* = 13.6 Hz, 1H), 2.60 (d, *J* = 13.6 Hz, 1H), 2.02–2.24 (m, 2H), 1.64 (m, 1H), 1.25 (s, 3H), 1.17 (m, 1H), 1.06 (s, 3H), 0.96 (s, 3H). ^13^C{^1^H} NMR (100 MHz, CDCl_3_): δ 206.3 (CH), 147.2 (C), 145.8 (C), 133.1 (C), 131.3 (C), 126.5 (CH), 123.6 (CH), 111.0 (CH), 107.8 (CH), 100.7 (CH_2_), 61.0 (C), 36.7 (C), 36.0 (CH_2_), 32.7 (CH_2_), 25.2 (CH_3_), 23.9 (CH_3_), 22.9 (CH_2_), 22.06 (CH_3_). IR (film): 2947, 1718, 1530, 1350, 1261, 1082, 1028, 806, 723, 687 cm^–1^. HRMS (ESI/TOF) *m*/*z*: [M + H]^+^ calcd for C_18_H_23_O_3_, 287.1647; found, 287.1651.
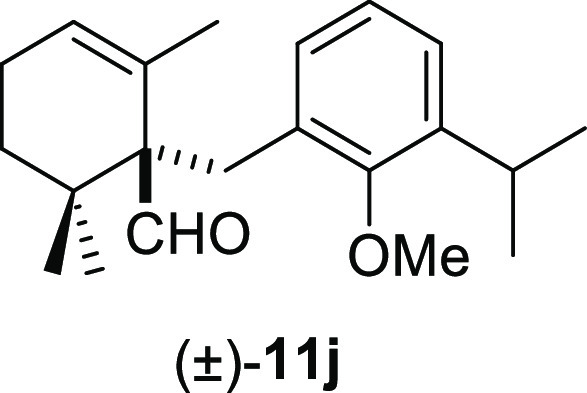


#### (±)-1-(3-Isopropyl-2-methoxybenzyl)-2,6,6-trimethylcyclohex-2-ene-1-carbaldehyde ((±)-**11j**)

To a solution of β-cyclocitral (**1**) (112 mg, 0.74 mmol) in toluene (12 mL), NaH (59 mg, 1.48 mmol), ^*t*^BuOK (244 mg, 0.81 mmol), and **10k** (270 mg, 1.11 mmol) were added and stirred for 45 min. Following the same workup used in the general procedure and after column chromatography, using 5% EtOAc/hexane, compound **11j** was obtained as a white solid (162 mg, 70%). ^1^H NMR (500 MHz, CDCl_3_): δ 9.75 (s, 1H), 7.07 (m, 1H), 7.02 (dd, *J* = 7.6, 1.7 Hz, 1H), 6.92 (t, *J* = 7.6 Hz, 1H), 5.63 (br s, 1H), 3.69 (s, 3H), 3.30 (d, *J* = 13.6 Hz, 1H), 3.26 (m, 1H), 2.94 (d, *J* = 13.6 Hz, 1H), 2.06 (m, 2H), 1.66 (m, 1H), 1.23 (m, 1H), 1.19 (d, *J* = 6.8 Hz, 3H), 1.16 (d, *J* = 6.8 Hz, 3H), 1.14 (br s, 3H), 0.99 (s, 3H), 0.98 (s, 3H). ^13^C{^1^H} NMR (125 MHz, CDCl_3_): δ 206.5 (CH), 156.3 (C), 141.8 (C), 132.4 (C), 131.3 (C), 129.7 (CH), 126.7 (CH), 124.7 (CH), 123.8 (CH), 61.5 (CH_3_), 60.8 (C), 36.8 (C), 32.9 (CH_2_), 30.2 (CH_2_), 26.5 (CH), 25.3 (CH_3_), 24.1 (CH_3_), 23.9 (CH_3_), 23.6 (CH_3_), 22.9 (CH_3_), 21.6 (CH_3_). IR (film): 2960, 2864, 1719, 1689, 1458, 1427, 1384, 1254, 1201, 1165, 1060, 1010, 796, 765, 568 cm^–1^. HRMS (ESI/TOF) *m*/*z*: [M + H]^+^ calcd for C_21_H_31_O_2_, 315.2324; found, 315.2326.
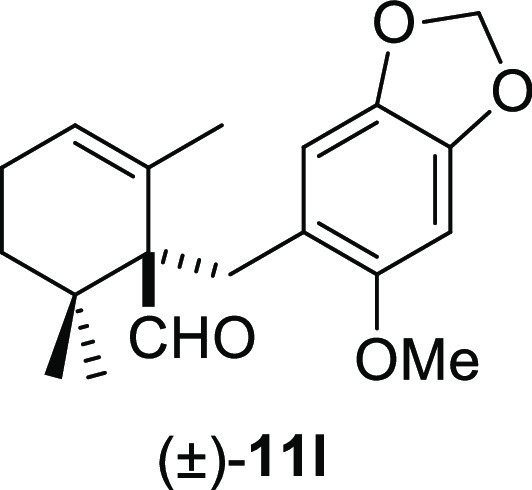


#### (±)-1-((6-Methoxybenzo[*d*][1,3]dioxol-5-yl)methyl)-2,6,6-trimethylcyclohex-2-ene-1-carbaldehyde ((±)-**11l**)

To a solution of β-cyclocitral (**1**) (147 mg, 0.97 mmol) in toluene (15 mL), NaH (78 mg, 1.94 mmol), ^*t*^BuOK (120 mg, 1.07 mmol), and **10l** (355 mg, 1.45 mmol) were added and stirred for 90 min. Following the same workup used in the general procedure and after column chromatography, using 5% EtOAc/hexane, compound **11l** was obtained as a colorless syrup (239 mg, 78%). ^1^H NMR (500 MHz, CDCl_3_): δ 9.72 (s, 1H), 6.67 (s, 1H), 6.40 (s, 1H), 5.83 (s, 2H), 5.59 (s, 1H), 3.64 (s, 3H), 3.22 (d, *J* = 13.5 Hz, 1H), 2.87 (d, *J* = 13.5 Hz, 1H), 2.05 (m, 2H), 1.82 (m, 1H), 1.15 (s, 3H), 1.14–1.10 (m, 1H), 0.97 (s, 6H). ^13^C{^1^H} NMR (125 MHz, CDCl_3_): δ 206.6 (CH), 152.8 (C), 146.3 (C), 140.6 (C), 131.5 (C), 125.8 (CH), 120.0 (C), 111.6 (CH), 100.8 (CH), 94.3 (CH_2_), 60.5 (C), 56.0 (CH_3_), 36.7 (C), 32.7 (CH_2_), 30.6 (CH_2_), 25.3 (CH_3_), 23.7 (CH_3_), 22.9 (CH_2_), 21.9 (CH_3_). IR (film): 2949, 2855, 1715, 1503, 1483, 1464, 1190, 1156, 1037, 1006, 935, 861, 824, 759 cm^–1^. HRMS (ESI/TOF) *m*/*z*: [M + H]^+^ calcd for C_19_H_25_O_4_, 317.1753; found, 317.1758.
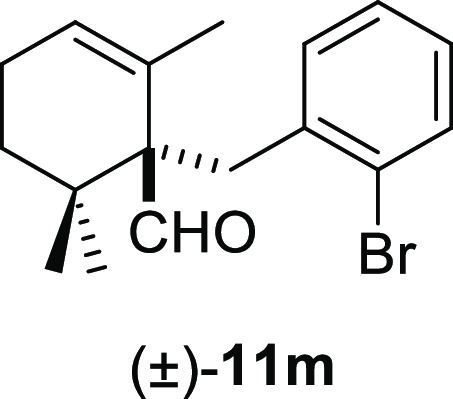


#### (±)-1-(2-Bromobenzyl)-2,6,6-trimethylcyclohex-2-ene-1-carbaldehyde ((±)-**11m**)

To a solution of β-cyclocitral (**1**) (310 mg, 2.04 mmol) in toluene (25 mL), NaH (163 mg, 4.08 mmol), ^*t*^BuOK (251 mg, 2.24 mmol), and **10m** (765 mg, 3.06 mmol) were added and stirred for 2 h. Following the same workup used in the general procedure and after column chromatography, using 3% EtOAc/hexane, compound **11m** was obtained as a colorless oil (602 mg, 92%). ^1^H NMR (400 MHz, CDCl_3_): δ 9.70 (s, 1H), 7.45 (dd, *J* = 7.5, 1.4 Hz, 1H), 7.35 (dd, *J* = 7.7, 1.8 Hz, 1H), 7.12 (td, *J* = 7.5, 1.4 Hz, 1H), 6.98 (td, *J* = 7.7, 1.8 Hz, 1H), 5.67 (br s, 1H), 3.59 (d, *J* = 13.3 Hz, 1H), 3.06 (d, *J* = 13.3 Hz, 1H), 2.21–2.11 (m, 2H), 2.10–1.99 (m, 1H), 1.25–1.17 (m, 1H), 1.05 (s, 3H), 0.98 (s, 3H), 0.94 (s, 3H). ^13^C{^1^H} NMR (100 MHz, CDCl_3_): δ 206.6 (CH), 139.3 (C), 132.9 (CH), 132.8 (CH), 129.9 (C), 127.74 (CH), 127.70 (CH), 127.0 (CH), 126.1 (C), 60.9 (C), 37.4 (C), 35.7 (CH_2_), 32.6 (CH_2_), 25.3 (CH_3_), 23.3 (CH_3_), 23.2 (CH_2_), 21.9 (CH_3_). IR (film): 2950, 2875, 2834, 1716, 1470, 1438, 1387, 1367, 1025, 873, 765, 747, 659, 569 cm^–1^. HRMS (ESI/TOF) *m*/*z*: [M + Na]^+^ calcd for C_17_H_22_OBrNa, 321.0854; found, 321.0862.
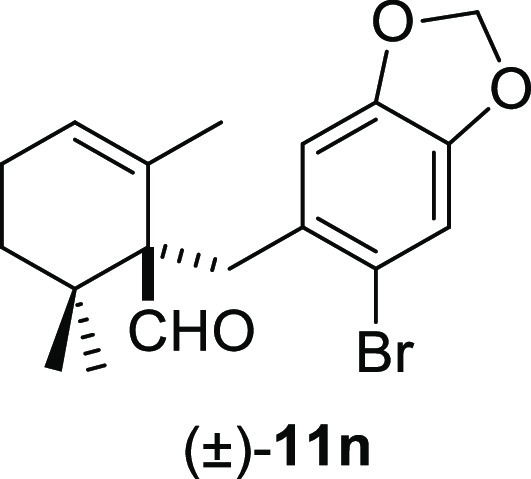


#### (±)-1-((6-Bromobenzo[*d*][1,3]dioxol-5-yl)methyl)-2,6,6-trimethylcyclohex-2-ene-1-carbaldehyde ((±)-**11n**)

To a solution of β-cyclocitral (**1**) (110 mg, 0.72 mmol) in toluene (12 mL), NaH (58 mg, 1.44 mmol), ^*t*^BuOK (89 mg, 0.79 mmol), and **10n** (317 mg, 1.08 mmol) were added and stirred for 2 h. Following the same workup used in the general procedure and after column chromatography, using 5% EtOAc/hexane, compound **11n** was obtained as a white solid (223 mg, 85%). ^1^H NMR (400 MHz, CDCl_3_): δ 9.68 (s, 1H), 6.90 (s, 1H), 6.86 (s, 1H), 5.89 (s, 2H), 5.68 (br s, 1H), 3.45 (d, *J* = 13.4 Hz, 1H), 2.96 (d, *J* = 13.4 Hz, 1H), 2.14 (m, 2H), 2.10–1.96 (m, 1H), 1.28–1.15 (m, 1H), 1.10 (s, 3H), 1.04 (s, 3H), 0.91 (s, 3H). ^13^C{^1^H} NMR (100 MHz, CDCl_3_): δ 206.7 (CH), 147.0 (C), 146.7 (C), 132.1 (C), 129.9 (C), 127.6 (CH), 116.0 (C), 112.6 (CH), 112.0 (CH), 101.5 (CH_2_), 60.8 (C), 37.3 (C), 35.7 (CH_2_), 32.6 (CH_2_), 25.3 (CH_3_), 23.3 (CH_3_), 23.1 (CH_2_), 22.2 (CH_3_). IR (film): 2921, 1715, 1502, 1474, 1407, 1388, 1367, 1267, 1227, 1167, 1113, 1037, 936, 873, 831, 756, 570 cm^–1^. HRMS (ESI/TOF) *m*/*z*: [M + H]^+^ calcd for C_18_H_22_O_3_Br, 365.0752; found, 365.0750.
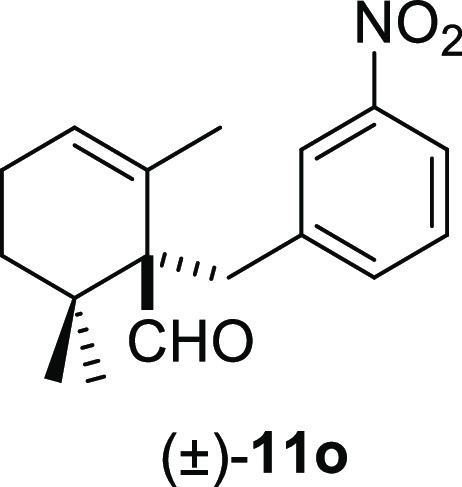


#### (±)-2,6,6-Trimethyl-1-(3-nitrobenzyl)cyclohex-2-ene-1-carbaldehyde ((±)-**11o**)

To a solution of β-cyclocitral (**1**) (192 mg, 1.26 mmol) in toluene (15 mL), NaH (101 mg, 2.52 mmol), ^*t*^BuOK (156 mg, 1.39 mmol), and **10o** (408 mg, 1.89 mmol) were added and stirred for 10 min. Following the same workup used in the general procedure and after column chromatography, using 8% EtOAc/hexane, compound **11o** was obtained as a yellow syrup (289 mg, 80%). ^1^H NMR (600 MHz, CDCl_3_): δ 9.69 (s, 1H), 8.02 (m, 2H), 7.55 (d, *J* = 7.8 Hz, 1H), 7.36 (t, *J* = 7.8 Hz, 1H), 5.81 (s, 1H), 3.49 (d, *J* = 13.3 Hz, 1H), 2.73 (d, *J* = 13.3 Hz, 1H), 2.23–2.06 (m, 2H), 1.57 (ddd, *J* = 14.0, 10.6, 7.0 Hz, 1H), 1.20 (ddd, *J* = 14.0, 7.0, 2.3 Hz, 1H), 1.13 (s, 3H), 1.07 (s, 3H), 0.96 (s, 3H). ^13^C{^1^H} NMR (150 MHz, CDCl_3_): δ 205.6 (CH), 148.0 (C), 141.7 (C), 137.0 (CH), 130.0 (C), 128.7 (CH), 127.5 (CH), 125.4 (CH), 121.2 (CH), 61.1 (C), 36.8 (C), 35.8 (CH_2_), 32.6 (CH_2_), 25.3 (CH_3_), 23.9 (CH_3_), 22.8 (CH_2_), 22.0 (CH_3_). IR (film): 2926, 1716, 1527, 1348, 1261, 1222, 1061, 1029, 804, 756, 723, 687, 672 cm^–1^. HRMS (ESI/TOF) *m*/*z*: [M + H]^+^ calcd for C_17_H_22_NO_3_, 288.1600; found, 288.1608.
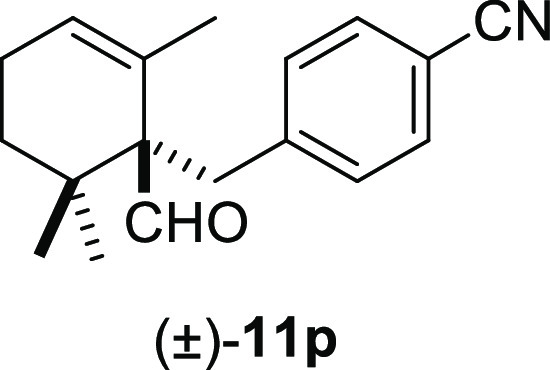


#### (±)-4-((1-Formyl-2,6,6-trimethylcyclohex-2-en-1-yl)methyl)benzonitrile ((±)-**11p**)

To a solution of β-cyclocitral (**1**) (166 mg, 1.09 mmol) in toluene (15 mL), NaH (87 mg, 2.18 mmol), ^*t*^BuOK (135 mg, 1.2 mmol), and **10p** (320 mg, 1.63 mmol) were added and stirred for 15 min. Following the same workup used in the general procedure and after column chromatography, using 5% EtOAc/hexane, compound **11p** was obtained as a yellow syrup (224 mg, 77%). ^1^H NMR (500 MHz, CDCl_3_): δ 9.67 (s, 1H), 7.46 (d, *J* = 8.4 Hz, 2H), 7.29 (d, *J* = 8.4 Hz, 2H), 5.76 (s, 1H), 3.43 (d, *J* = 13.2 Hz, 1H), 2.66 (d, *J* = 13.2 Hz, 1H), 2.20–2.03 (m, 2H), 1.64–1.51 (m, 1H), 1.19 (ddd, *J* = 13.9, 6.8, 2.2 Hz, 1H), 1.09 (s, 3H), 1.04 (s, 3H), 0.92 (s, 3H). ^13^C{^1^H} NMR (125 MHz, CDCl_3_): δ 205.4 (CH), 145.5 (C), 131.6 (2× CH), 131.5 (2× CH), 130.2 (C), 127.3 (CH), 119.0 (C), 109.9 (C), 61.3 (C), 36.8 (C), 36.4 (CH_2_), 32.6 (CH_2_), 25.2 (CH_3_), 23.8 (CH_3_), 22.8 (CH_2_), 22.0 (CH_3_). IR (film): 2962, 2228, 1719, 1606, 1365, 1174, 1018, 816, 754, 816, 754 cm^–1^. HRMS (ESI/TOF) *m*/*z*: [M-CH_2_+H]^+^ calcd for C_17_H_20_NO, 254.1545; found, 254.1543.
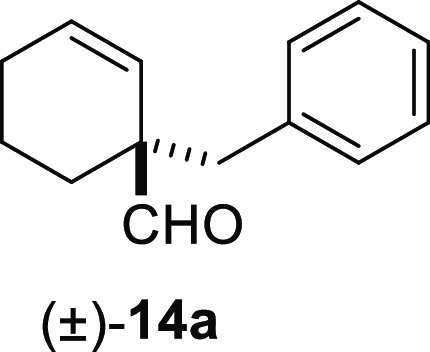


#### (±)-1-Benzylcyclohex-2-enecarbaldehyde ((±)-**14a**)

To a solution of **13a** (130 mg, 1.18 mmol) in toluene (15 mL), NaH (95 mg, 2.36 mmol), ^*t*^BuOK (145 mg, 1.3 mmol), and benzyl bromide (**10e**) (303 mg, 1.77 mmol) were added and stirred for 20 min. Following the same workup used in the general procedure and after column chromatography, using 5% EtOAc/hexane, compound **14a** was obtained as a colorless oil (224 mg, 95%). ^1^H NMR (500 MHz, CDCl_3_): δ 9.55 (s, 1H), 7.30–7.10 (m, 5H), 6.01 (dt, *J* = 10.0, 3.8 Hz, 1H), 5.58 (d, *J* = 10.0 Hz, 1H), 2.90 (s, 2H), 2.05–1.88 (m, 3H), 1.64–1.52 (m, 3H). ^13^C{^1^H} NMR (125 MHz, CDCl_3_): δ 203.3 (CH), 136.5 (C), 132.0 (CH), 130.3 (2× CH), 128.1 (2× CH), 126.5 (CH), 126.3 (CH), 52.0 (C), 42.6 (CH_2_), 27.9 (CH_2_), 24.8 (CH_2_), 18.8 (CH_2_). IR (film): 3027, 2934, 2867, 2835, 1722, 1495, 1453, 1069, 762, 729, 701, 679 cm^–1^. HRMS (ESI/TOF) *m*/*z*: [M + H]^+^ calcd for C_14_H_17_O, 201.1279; found, 201.1282.
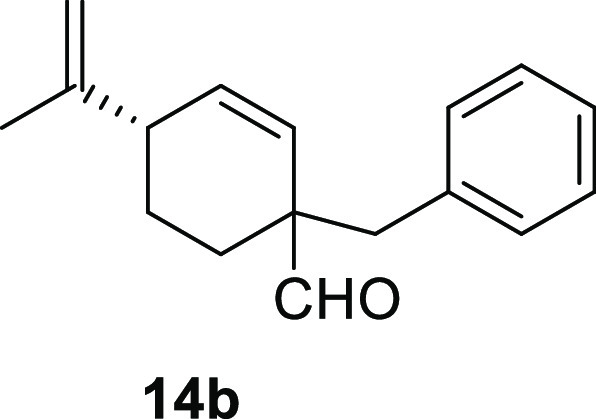


#### (1*R*,4*S*) and (1*S*,4*S*)-1-Benzyl-4-(prop-1-en-2-yl)cyclohex-2-enecarbaldehyde (**14b**)

To a solution of **13b** (150 mg, 1 mmol) in toluene (15 mL), NaH (80 mg, 2 mmol), ^*t*^BuOK (123 mg, 1.1 mmol), and benzyl bromide (**10e**) (256 mg, 1.5 mmol) were added and stirred for 20 min. Following the same workup used in the general procedure and after column chromatography, using 5% EtOAc/hexane, compound **14b** was obtained as a colorless oil (223 mg, 93%, approx. 1:1 diasteroisomeric ratio). ^1^H NMR (400 MHz, CDCl_3_): δ 9.56 (s, 2H), 7.39–7.10 (m, 10H), 5.91–5.86 (m, 2H), 5.68 (br s, 1H), 5.66 (br s, 1H), 4.77 (br s, 1H), 4.73 (br s, 1H), 4.71 (br s, 1H), 4.44 (br s, 1H), 2.91–2.87 (m, 4H), 2.68 (m, 2H), 2.01–1.98 (m, 2H), 1.81–1.61 (m, 4H), 1.69 (s, 6H), 1.55–1.42 (m, 2H). Major isomer: ^13^C{^1^H} NMR (125 MHz, CDCl_3_): δ 202.7 (CH), 147.8 (C), 136.3 (C), 135.1 (CH), 130.3 (2× CH), 128.1 (2× CH), 127.0 (CH), 126.6 (CH), 110.9 (CH_2_), 52.1 (C), 42.9 (CH), 42.6 (CH_2_), 27.1 (CH_2_), 24.7 (CH_2_), 20.6 (CH_3_). Minor isomer: ^13^C{^1^H} NMR (125 MHz, CDCl_3_): δ 203.0 (CH), 147.1 (C), 136.3 (C), 134.3 (CH), 130.4 (2× CH), 128.1 (2× CH), 126.9 (CH), 126.6 (CH), 111.6 (CH_2_), 52.3 (C), 42.1 (CH_2_), 41.8 (CH), 24.7 (CH_2_), 23.3 (CH_2_), 21.4 (CH_3_). IR (film): 2936, 1720, 1644, 1496, 1453, 1374, 892, 831, 762, 735, 700 cm^–1^. HRMS (ESI/TOF) *m*/*z*: [M + H]^+^ calcd for C_17_H_21_O, 241.1592; found, 241.1601.
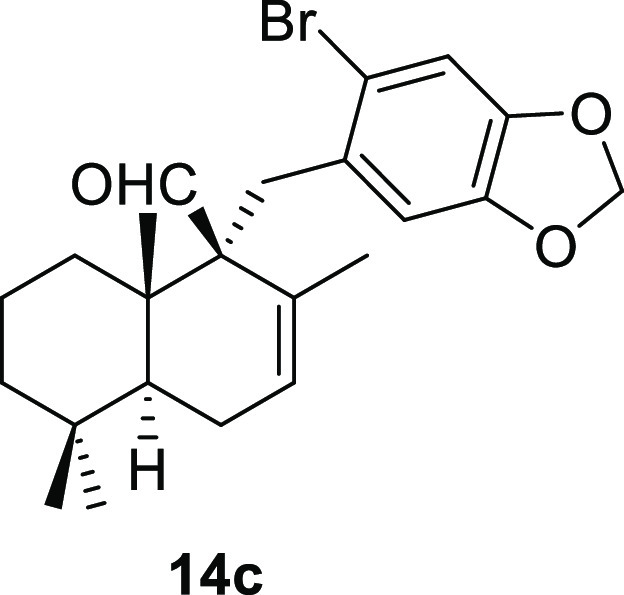


#### (1*S*,4*aS*,8*aS*)-1-((6-Bromobenzo[*d*][1,3]dioxol-5-yl)methyl)-2,5,5,8*a*-tetramethyl-1,4,4*a*,5,6,7,8,8*a*-octahydronaphthalene-1-carbaldehyde (**14c**)

To a solution of **13c** (76 mg, 0.34 mmol) in toluene (10 mL), NaH (30 mg, 0.75 mmol), ^*t*^BuOK (43 mg, 0.38 mmol), and **10n** (153 mg, 0.52 mmol) were added and stirred for 2 h. Following the same workup used in the general procedure and after column chromatography, using 5% EtOAc/hexane, compound **14c** was obtained as a colorless syrup (128 mg, 86%). [α]_D_^20^ −3.0 (*c* 0.6, CHCl_3_). ^1^H NMR (400 MHz, CDCl_3_): δ 9.77 (s, 1H), 6.90 (s, 1H), 6.75 (s, 1H), 5.89 (s, 2H), 5.70 (br s, 1H), 3.52 (d, *J* = 13.3 Hz, 1H), 3.01 (d, *J* = 13.3 Hz, 1H), 2.19–2.12 (m, 1H), 2.01–1.91 (m, 1H), 1.59–1.42 (m, 5H), 1.26–1.16 (m, 2H), 1.10 (s, 3H), 1.07 (s, 3H), 0.96 (s, 3H), 0.93 (s, 3H). ^13^C{^1^H} NMR (100 MHz, CDCl_3_): δ (ppm) 207.9 (CH), 147.0 (C), 146.7 (C), 132.2 (C), 129.5 (C), 128.4 (CH), 116.1 (C), 112.6 (CH), 112.1 (CH), 101.5 (CH_2_), 63.0 (C), 42.3 (C), 42.0 (CH_2_), 41.8 (CH), 36.0 (CH_2_), 33.9 (CH_3_), 33.4 (CH_2_), 33.3 (C), 24.7 (CH_2_), 22.3 (CH_3_), 22.0 (CH_3_), 18.2 (CH_2_), 17.5 (CH_3_). IR (film): 2949, 1716, 1672, 1503, 1478, 1367, 1228, 1114, 1039, 936, 882, 841, 655, 567 cm^–1^. HRMS (ESI/TOF) *m*/*z*: [M + H]^+^ calcd for C_23_H_30_O_3_Br, 433.1378; found, 433.1365.
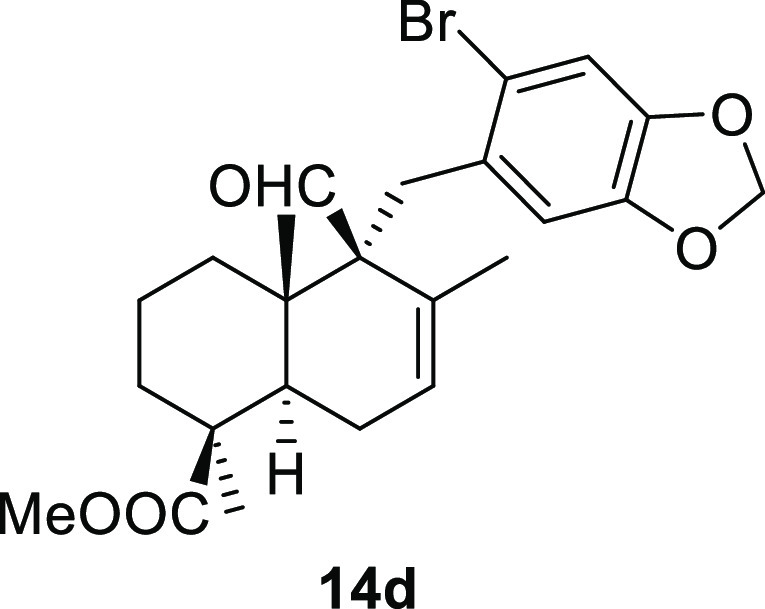


#### (1*S*,4*aS*,5*S*,8*aR*)-Methyl 5-((6-Bromobenzo[*d*][1,3]dioxol-5-yl)methyl)-5-formyl-1,4*a*,6-trimethyl-1,2,3,4,4*a*,5,8,8*a*-octahydronaphthalene-1-carboxylate (**14d**)

To a solution of **13d** (83 mg, 0.31 mmol) in toluene (10 mL), NaH (26 mg, 0.65 mmol), ^*t*^BuOK (39 mg, 0.35 mmol), and **10n** (138 mg, 0.47 mmol) were added and stirred for 2 h. Following the same workup used in the general procedure and after column chromatography, using 5% EtOAc/hexane, compound **14d** was obtained as a colorless syrup (133 mg, 90%). [α]_D_^20^ −26.0 (*c* 0.3, CHCl_3_). ^1^H NMR (400 MHz, CDCl_3_): δ 9.74 (s, 1H), 6.89 (s, 1H), 6.76 (s, 1H), 5.88 (s, 2H), 5.68 (s, 1H), 3.64 (s, 3H), 3.49 (d, *J* = 13.3 Hz, 1H), 2.94 (d, *J* = 13.3 Hz, 1H), 2.71–2.56 (m, 1H), 2.41–2.29 (m, 1H), 2.22–2.06 (m, 2H), 1.90–1.41 (m, 4H), 1.23 (s, 3H), 1.05 (s, 3H), 0.95 (s, 3H), 0.87–0.78 (m, 1H). ^13^C{^1^H} NMR (100 MHz, CDCl_3_): δ 207.2 (CH), 177.7 (C), 147.0 (C), 146.7 (C), 131.8 (C), 128.3 (C), 128.3 (CH), 116.1 (C), 112.6 (CH), 112.1 (CH), 101.5 (CH_2_), 62.3 (C), 51.4 (CH_3_), 44.3 (CH), 44.1 (C), 41.8 (C), 38.0 (CH_2_), 35.9 (CH_2_), 33.0 (CH_2_), 29.1 (CH_3_), 25.3 (CH_2_), 21.8 (CH_3_), 19.0 (CH_2_), 16.4 (CH_3_). IR (film): 2949, 1715, 1503, 1477, 1407, 1380, 1226, 1166, 1143, 1113, 1038, 984, 934, 910, 874, 841, 771, 730, 652, 566 cm^–1^. HRMS (ESI/TOF) *m*/*z*: [M + H]^+^ calcd for C_24_H_30_O_5_Br, 477.1277; found, 477.1276.
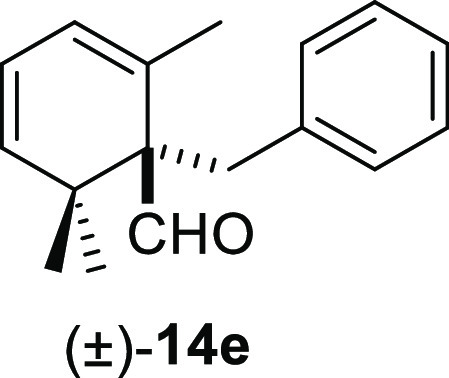


#### (±)-1-Benzyl-2,6,6-trimethylcyclohexa-2,4-diene-1-carbaldehyde ((±)-**14e**)

To a solution of **13e** (163 mg, 1.08 mmol) in toluene (15 mL), NaH (84 mg, 2.1 mmol), ^*t*^BuOK (134 mg, 1.19 mmol), and benzyl bromide (**10e**) (277 mg, 1.62 mmol) were added and stirred for 3 h. Following the same workup used in the general procedure and after column chromatography, using 5% EtOAc/hexane, compound **14e** was obtained as a colorless syrup (188 mg, 72%). ^1^H NMR (500 MHz, CDCl_3_): δ 9.98 (s, 1H), 7.18–7.10 (m, 5H), 5.90 (m, 2H), 5.35 (m, 1H), 3.27 (d, *J* = 12.8 Hz, 1H), 2.87 (d, *J* = 12.8 Hz, 1H), 1.14 (s, 3H), 1.13 (s, 3H), 1.10 (s, 3H). ^13^C{^1^H} NMR (125 MHz, CDCl_3_): δ 206.8 (C), 138.8 (C), 135.8 (CH), 135.1 (C), 130.9 (CH), 127.6 (CH), 125.9 (CH), 121.9 (CH), 121.6 (CH), 61.8 (C), 40.1 (C), 30.8 (CH_2_), 22.5 (CH_3_), 22.4 (CH_3_), 22.0 (CH_3_). IR (film): 3028, 2961, 1718, 1494, 1453, 1362, 1076, 726, 700 cm^–1^. HRMS (ESI/TOF) *m*/*z*: [M–CH_3_]^+^ calcd for C_16_H_19_O, 227.1436; found, 227.1443.

### Preparation of (4*aS*,8*aS*)-2,5,5,8*a*-Tetramethyl-3,4,4*a*,5,6,7,8,8*a*-octahydronaphthalene-1-carbaldehyde (**13c**)

Compound **13c** was prepared following the procedure described in the literature. The spectroscopic data were in agreement with those described in the literature.^20^

### Preparation of (1*S*,4*aS*,8*aR*)-Methyl 5-Formyl-1,4*a*,6-trimethyl-1,2,3,4,4*a*,7,8,8*a*-octahydronaphthalene-1-carboxylate (**13d**)

Compound **13d** was prepared following the procedure described in the literature. The spectroscopic data were in agreement with those described in the literature.^21^

### General Procedure for Deformylation of β,γ-Unsaturated Aldehydes (±)-**11a**, (±)-**11e**, (±)-**11i**, (±)-**11m**, and (±)-**11o** with Pb(OAc)_4_

To a solution of the corresponding β,γ-unsaturated aldehydes (±)-**11a**, (±)-**11e**, (±)-**11i**, (±)-**11m**, and (±)-**11o** (1 mmol) in dry benzene (7 mL) was added Pb(OAc)_4_ (1.1 mmol), and the solution was heated at 80 °C for 10 min^–2^ h. Then, the reaction mixture was quenched with 5% Na_2_SO_3_ (5 mL), and the aqueous layer was extracted with two portions of ethyl acetate (2 × 15 mL). The combined organic solution was washed with brine (10 mL), dried over anhydrous sodium sulfate, and filtered. The solvent was evaporated under reduced pressure to give a crude product which was purified by silica gel column chromatography (petroleum ether/ethyl acetate), affording the corresponding allyl acetates (±)-**15a**, (±)-**15e**, (±)-**15i**, (±)-**15m**, and (±)-**15o** in the yields shown in Table 4.
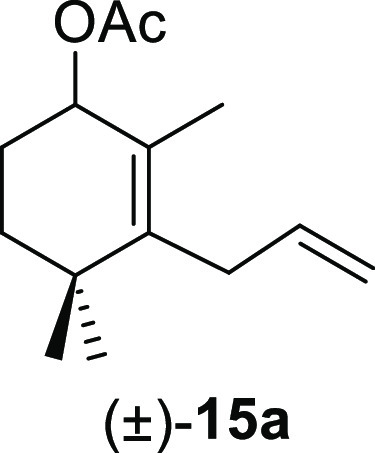


#### (±)-3-Allyl-2,4,4-trimethylcyclohex-2-en-1-yl Acetate ((±)-**15a**)

To a solution of **11a** (194 mg, 1.01 mmol) in benzene (7 mL) was added Pb(OAc)_4_ (492 mg, 1.11 mmol), and the mixture was heated at 80 °C for 7 min. Following the same workup used in the general procedure, **15a** (204 mg, 91%) was obtained as a colorless syrup after column chromatography using 3% EtOAc/hexane. ^1^H NMR (500 MHz, CDCl_3_): δ 5.68 (m, 1H), 5.09 (t, *J* = 4.6 Hz, 1H), 4.95 (m, 1H), 4.81 (m, 1H), 2.76 (d, *J* = 5.9 Hz, 2H), 1.99 (s, 3H), 1.84 (m, 1H), 1.64 (m, 1H), 1.54 (m, 1H), 1.51 (s, 3H), 1.31 (ddd, *J* = 13.3, 7.0, 3.2 Hz, 1H), 0.97 (s, 3H), 0.91 (s, 3H). ^13^C{^1^H} NMR (100 MHz, CDCl_3_): δ 171.2 (C), 141.7 (C), 136.5 (CH), 126.8 (C), 114.9 (CH_2_), 72.7 (CH), 35.3 (C), 34.8 (CH_2_), 32.8 (CH_2_), 28.2 (CH_3_), 26.8 (CH_3_), 25.4 (CH_2_), 21.4 (CH_3_), 16.6 (CH_3_). IR (film): 1719, 1634, 1469, 1370, 1244, 1174, 1145, 1017, 994, 961, 910, 865 cm^–1^. HRMS (ESI/TOF) *m*/*z*: [M–OAc]^+^ calcd for C_12_H_19_, 163.1487; found, 163.1483.
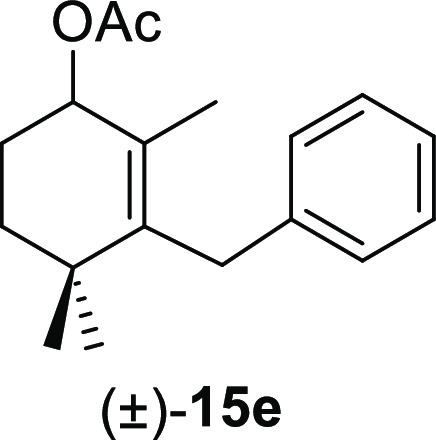


#### (±)-3-Benzyl-2,4,4-trimethylcyclohex-2-en-1-yl Acetate ((±)-**15e**)

To a solution of **11e** (143 mg, 0.59 mmol) in benzene (4 mL) was added Pb(OAc)_4_ (288 mg, 0.65 mmol), and the mixture was heated at 80 °C for 2 h. Following the same workup used in the general procedure, **15e** (114 mg, 71%) was obtained as a colorless syrup after column chromatography using 5% EtOAc/hexane. ^1^H NMR (400 MHz, CDCl_3_): δ 7.29–7.23 (m, 2H), 7.19–7.10 (m, 3H), 5.27 (d, *J* = 4.8 Hz, 1H), 3.50 (s, 2H), 2.09 (s, 3H), 1.96 (dddd, *J* = 14.3, 11.4, 4.8, 3.2 Hz, 1H), 1.75 (dddd, *J* = 14.3, 7.2, 4.8, 3.2 Hz, 1H), 1.66 (ddd, *J* = 13.8, 11.4, 3.2 Hz, 1H), 1.54 (s, 3H), 1.40 (ddd, *J* = 13.8, 7.2, 3.2 Hz, 1H), 0.96 (s, 3H), 0.93 (s, 3H). ^13^C{^1^H} NMR (100 MHz, CDCl_3_): δ 171.2 (C), 141.8 (C), 140.2 (C), 128.2 (2× CH), 128.0 (C), 127.8 (2× CH), 125.5 (CH), 72.6 (CH), 35.4 (C), 35.0 (CH_2_), 34.1 (CH_2_), 28.5 (CH_3_), 27.2 (CH_3_), 25.5 (CH_2_), 21.5 (CH_3_), 17.3 (CH_3_). IR (film): 2957, 2935, 2860, 1732, 1494, 1452, 1370, 1243, 1018, 961, 715 cm^–1^. HRMS (ESI/TOF) *m*/*z*: [M + Na]^+^ calcd for C_18_H_24_O_2_Na, 295.1674; found, 295.1679.
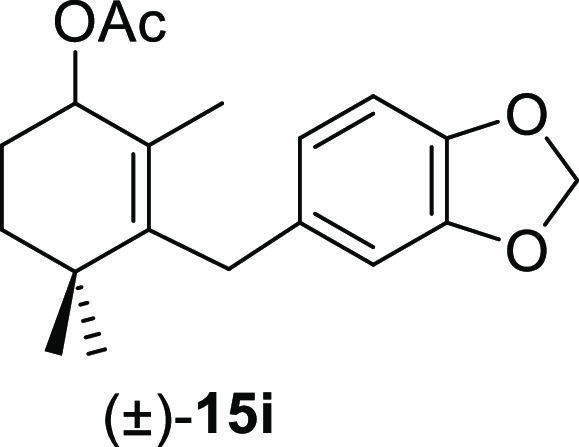


#### (±)-3-(Benzo[*d*][1,3]dioxol-5-ylmethyl)-2,4,4-trimethylcyclohex-2-en-1-yl Acetate ((±)-**15i**)

To a solution of **11i** (204 mg, 0.71 mmol) in benzene (5 mL) was added Pb(OAc)_4_ (348 mg, 0.78 mmol), and the mixture was heated at 80 °C for 50 min. Following the same workup used in the general procedure, **15i** (206 mg, 92%) was obtained as an amorphous solid after column chromatography using 3% EtOAc/hexane. ^1^H NMR (400 MHz, CDCl_3_): δ 6.71 (d, *J* = 8.0 Hz, 1H), 6.63 (br s, 1H), 6.59 (br d, *J* = 8.0 Hz, 1H), 5.91 (s, 2H), 5.25 (t, *J* = 4.7 Hz, 1H), 3.43 (d, *J* = 16.3 Hz, 1H), 3.39 (d, *J* = 16.3 Hz, 1H), 2.09 (s, 3H), 1.94 (m, 1H), 1.76 (m, 1H), 1.65 (ddd, *J* = 12.3, 12.3, 3.1, 1H), 1.55 (s, 3H), 1.41 (ddd, *J* = 13.1, 7.1 3.2 Hz, 1H), 0.95 (s, 3H), 0.93 (s, 3H). ^13^C{^1^H} NMR (100 MHz, CDCl_3_): δ 171.2 (C), 147.6 (C), 145.4 (C), 142.0 (CH), 134.1 (C), 128.1 (C), 120.6 (CH), 108.3 (CH), 108.1 (CH), 100.7 (CH_2_), 72.5 (C), 35.4 (C), 34.9 (CH_2_), 33.7 (CH_2_), 28.5 (CH_3_), 27.2 (CH_3_), 25.5 (CH_2_), 21.4 (CH_3_), 17.3 (CH_3_). IR (film): 1723, 1605, 1495, 1483, 1434, 1365, 1226, 1344, 1227, 1175, 1146, 1121, 1091, 1037, 1017, 923, 875, 793 cm^–1^. HRMS (ESI/TOF) *m*/*z*: [M + H]^+^ calcd for C_19_H_25_O_4_, 317.1753; found, 317.1751.
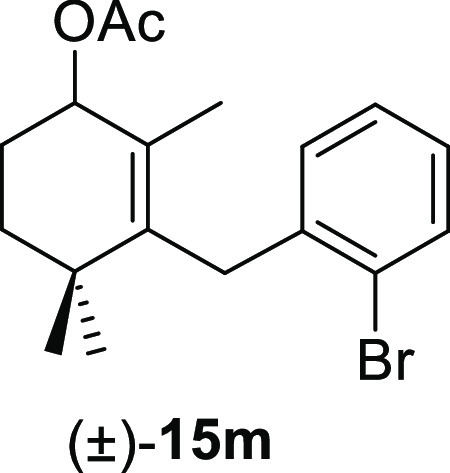


#### (±)-3-(2-Bromobenzyl)-2,4,4-trimethylcyclohex-2-en-1-yl Acetate ((±)-**15m**)

To a solution of **11m** (53 mg, 0.16 mmol) in benzene (1 mL) was added Pb(OAc)_4_ (80 mg, 0.18 mmol), and the mixture was heated at 80 °C for 1 h. Following the same workup used in the general procedure, **15m** (48 mg, 83%) was obtained as a colorless syrup after column chromatography using 5% EtOAc/hexane. ^1^H NMR (500 MHz, CDCl_3_): δ 7.54 (dd, *J* = 7.8, 1.3 Hz, 1H), 7.22 (td, *J* = 7.5, 1.3 Hz, 1H), 7.08 (d, *J* = 7.8 Hz, 1H), 7.04 (t, *J* = 7.5 Hz, 1H), 5.28 (m, 1H), 3.48 (d, *J* = 17.5 Hz, 1H), 3.44 (d, *J* = 17.5 Hz, 1H), 2.09 (s, 3H), 1.98 (dddd, *J* = 14.2, 11.3, 5.0, 3.2 Hz, 1H), 1.80 (dddd, *J* = 14.2, 7.5, 4.6, 3.2 Hz, 1H), 1.69 (ddd, *J* = 13.4, 11.3, 3.2 Hz, 1H), 1.48–1.44 (m, 1H), 1.46 (s, 3H), 0.95 (s, 6H). ^13^C{^1^H} NMR (125 MHz, CDCl_3_): δ 171.1 (C), 141.1 (C), 138.7 (C), 132.5 (CH), 129.1 (C), 128.7 (CH), 127.3 (CH), 127.1 (CH), 125.1 (C), 72.4 (CH), 35.3 (C), 34.8 (CH_2_), 34.7 (CH_2_), 28.2 (CH_3_), 27.0 (CH_3_), 25.4 (CH_2_), 21.4 (CH_3_), 17.1 (CH_3_). IR (film): 1723, 1438, 1387, 1231, 1155, 1095, 1076, 1025, 877, 770 cm^–1^. HRMS (ESI/TOF) *m*/*z*: [M–OAc]^+^ calcd for C_16_H_20_Br, 291.0748; found, 291.0754.
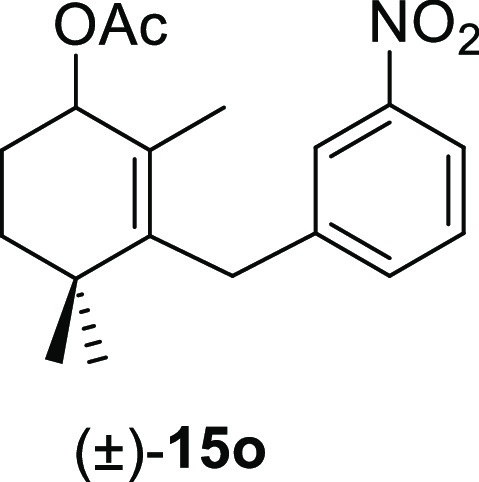


#### (±)-2,4,4-Trimethyl-3-(3-nitrobenzyl)cyclohex-2-en-1-yl Acetate ((±)-**15o**)

To a solution of **11o** (67 mg, 0.23 mmol) in benzene (2 mL) was added Pb(OAc)_4_ (115 mg, 0.26 mmol), and the mixture was heated at 80 °C for 90 min. Following the same workup used in the general procedure, **15o** (62 mg, 85%) was obtained as a colorless syrup after column chromatography using 3% EtOAc/hexane. ^1^H NMR (400 MHz, CDCl_3_): δ 8.00 (m, 2H), 7.44 (m, 2H), 5.27 (t, *J* = 4.7 Hz, 1H), 3.60 (d, *J* = 17.2 Hz, 1H), 3.57 (d, *J* = 17.2 Hz, 1H), 2.10 (s, 3H), 1.96 (m, 1H), 1.79 (m, 1H), 1.69 (ddd, *J* = 14.4, 11.6, 3.1 Hz, 1H), 1.52 (s, 3H), 1.43 (m, 1H), 0.94 (s, 3H), 0.93 (s, 3H). ^13^C{^1^H} NMR (100 MHz, CDCl_3_): δ 171.1 (C), 148.5 (C), 142.4 (C), 140.6 (C), 134.0 (CH), 129.4 (C), 129.1 (CH), 122.8 (CH), 120.9 (CH), 72.1 (CH), 35.4 (C), 34.7 (CH_2_), 33.6 (CH_2_), 28.4 (CH_3_), 27.1 (CH_3_), 25.3 (CH_2_), 21.4 (CH_3_), 17.4 (CH_3_). IR (film): 1725, 1469, 1696, 1346, 1231, 1169, 1144, 1095, 1076, 1017, 996, 961, 865, 802 cm^–1^. HRMS (ESI/TOF) *m*/*z*: [M–OAc]^+^ calcd for C_16_H_20_NO_2_, 258.1494; found, 258.1488.

## Supporting Information Available

Copies of ^1^H and ^13^C{^1^H} NMR spectra for all new compounds (PDF)

## References

[ref1] For an example of the use of Taxol, see: Kanda Y.; Ishihara Y.; Wilde N. C.; Baran P. S. Two-phase total synthesis of taxanes: Tactics and strategies. J. Org. Chem. 2020, 85, 10293–10320. 10.1021/acs.joc.0c01287. 32663002

[ref2] For an example of the use of Artemisin, see: D’Alessandro S.; Scaccabarozzi D.; Signorini L.; Perego F.; Ilboudo D. P.; Ferrante P.; Delbue S. The use of antimalarial drugs against viral infection. Microorganisms 2020, 8, 85 10.3390/microorganisms8010085. PMC7022795 31936284

[ref3] For an example of the use of Cantharidin, see: Naz F.; Wu Y.; Zhang N.; Yang Z.; Yu C. Anticancer attributes of Cantharidin: Involved molecular mechanisms and pathways. Molecules 2020, 25, 3279 10.3390/molecules25143279. PMC7397086 32707651

[ref4] aFor a recent review concerning the total synthesis of complex terpene natural products, utilizing terpene building blocks, see: Brill Z. G.; Condakes M. L.; Ting C. P.; Maimone T. J. Navigating the chiral pool in the total synthesis of complex terpene natural products. Chem. Rev. 2017, 117, 11753–11795. 10.1021/acs.chemrev.6b00834. 28293944 PMC5638449

[ref5] Matsumoto T.; Usui S.; Morimoto T. A convenient synthesis of (±)-taxodione, (±)-ferruginol, and (±)-sugiol. Bull. Chem. Soc. Jpn. 1977, 50, 1575–1579. 10.1246/bcsj.50.1575.

[ref6] Matsumoto T.; Ohmura T.; Usui S. The revised structure of dispermol and total synthesis of maytenoquinone, dispermol, and dispermone. Bull. Chem. Soc. Jpn. 1979, 52, 1957–1963. 10.1246/bcsj.52.1957.

[ref7] Huang J.; Foyle D.; Lin X.; Yang J. Total synthesis and biological evaluation of an antifungal tricyclic o-hydroxy-p-quinone methide diterpenoid. J. Org. Chem. 2013, 78, 9166–9173. 10.1021/jo4013964. 23957833 PMC3843042

[ref8] aAlvarez-Manzaneda E.; Chahboun R.; Cabrera E.; Alvarez E.; Alvarez-Manzaneda R.; Meneses R.; Es-Samti H.; Fernández A. A very efficient route toward the 4a-methyltetrahydrofluorene skeleton: Short synthesis of (±)-dichroanone and (±)-taiwaniaquinone H. J. Org. Chem. 2009, 74, 3384–3388. 10.1021/jo900153y. 19348490

[ref9] De Graaf S. A. G.; Oosterhoff P. E. R.; van der Gen A. Direct alkylation of α,β-unsaturated aldehydes. Tetrahedron Lett. 1974, 15, 1653–1656. 10.1016/s0040-4039(01)82544-2.

[ref10] Hall J. B.; Wiegers W. J. Process for the alkylation of α, β-unsaturated aldehydes. U.S. Patent 4,010,207 A, 1977.

[ref11] aKimura M.; Horino Y.; Mukai R.; Tanaka S.; Tamaru Y. Strikingly simple direct α-allylation of aldehydes with allylic alcohols: Remarkable advance in the Tsuji-Trost reaction. J. Am. Chem. Soc. 2001, 123, 10401–10402. 10.1021/ja011656a. 11603997

[ref12] Yamashita M.; Matsumiya K.; Nakano K.-i. Organic synthesis via dialkylhydrazones. Part 9. α-Alkylation of α,β -unsaturated aldehyde dimethylhydrazones accompanied with the double bond migration to β,γ. Bull. Chem. Soc. Jpn. 1993, 66, 1759–1763. 10.1246/bcsj.66.1759.

[ref13] Yang X.; Nath D.; Morse J.; Ogle C.; Yurtoglu E.; Altundas R.; Fleming F. Cyclic alkenenitriles: Copper-catalyzed deconjugative α -alkylation. J. Org. Chem. 2016, 81, 4098–4102. 10.1021/acs.joc.6b00367. 27171565

[ref14] Shi H.-N.; Huang M.-H.; Hao W.-J.; Tu X.-C.; Tu S.-J.; Jiang B. Synthesis of diastereoenriched 1-indanones via double-base cooperatively promoted 1,4-oxo-migration/cyclization of β-alkynyl ketones. J. Org. Chem. 2019, 84, 16027–16035. 10.1021/acs.joc.9b02525. 31769289

[ref15] Khartulyari A. S.; Kapur M.; Maier M. E. Concise strategy to the core structure of the macrolide Queenslandon. Org. Lett. 2006, 8, 5833–5836. 10.1021/ol062479r. 17134284

[ref16] Akbaba Y.; Türker Balaydın H.; Göksu S.; Şahin E.; Menzek A. Total synthesis of the Biologically active, naturally occurring 3,4-dibromo-5-[2-bromo-3,4-dihydroxy-6-(methoxymethyl)benzyl]benzene-1,2-diol and regioselective O-demethylation of aryl methyl ethers. Helv. Chim. Acta 2010, 93, 1127–1135. 10.1002/hlca.200900300.

[ref17] Drew S. L.; Lawrence A. L.; Sherburn M. S. Total synthesis of Kingianins A, D, and F. Angew. Chem., Int. Ed. 2013, 52, 4221–4224. 10.1002/anie.201210084. 23468400

[ref18] Spring D. R.; Krishnan S.; Blackwell H. E.; Schreiber S. L. Diversity-oriented synthesis of biaryl-containing medium rings using a one bead/one stock solution platform. J. Am. Chem. Soc. 2002, 124, 1354–1363. 10.1021/ja017248o. 11841305

[ref19] Cantillo D.; de Frutos O.; Rincon J. A.; Mateos C.; Kappe C. O. A scalable procedure for light-induced benzylic brominations in continuous flow. J. Org. Chem. 2014, 79, 223–229. 10.1021/jo402409k. 24261546

[ref20] aBarrero A. F.; Alvarez-Manzaneda E. J.; Chahboun R. Synthesis of wiedendiol-A and wiedendiol-B from labdane diterpenes. Tetrahedron 1998, 54, 5635–5650. 10.1016/s0040-4020(98)00235-x.

[ref21] For the synthesis of the benzofluorene derivative Dasyscyphin E using Δ^7^-drimenal as electrophile see: Jiménez F.; Fernández A.; Boulifa E.; Mansour A. I.; Alvarez-Manzaneda R.; Chahboun R.; Alvarez-Manzaneda E. Diastereoselective intramolecular Heck reaction assisted by an acetate group: Synthesis of the decahydrobenzofluorene derivative Dasyscyphin E. J. Org. Chem. 2017, 82, 9550–9559. 10.1021/acs.joc.7b01551. 28809119

[ref22] Preite M. D.; Cuellar M. A. A new reaction: lead(IV) acetate-mediated oxidative fragmentation of homoallylic alcohols. Chem. Commun. 2004, 1970–1971. 10.1039/b405986g. 15340626

[ref23] aBertrand M. P.; Surzur J. M.; Boyer M.; Mihailović M. L. Mechanisms of oxidation of ethylenic alcohols by lead tetraacetate. ESR evidence for the influence of experimental conditions on the homolytic or heterolytic course of the reaction. Tetrahedron 1979, 35, 1365–1372. 10.1016/0040-4020(79)85030-9.

[ref24] Aslam S. N.; Stevenson P. C.; Phythian S. J.; Veitch N. C.; Hall D. R. Synthesis of cicerfuran, an antifungal benzofuran, and some related analogs. Tetrahedron 2006, 62, 4214–4226. 10.1016/j.tet.2006.02.015.

