# Supplementary material for: Deconjugative α-Alkylation of Cyclohexenecarboxaldehydes: An Access to Diverse Terpenoids
Source: J Org Chem. 2021 Jun 15;86(13):8742–54. doi: 10.1021/acs.joc.1c00560 (PMC8901105; doi:10.1021/acs.joc.1c00560)
Supplement: Supplementary file 1 — jo1c00560_si_001.pdf [file jo1c00560_si_001.pdf]

Deconjugative α-Alkylation of Cyclohexenecarboxaldehydes: An Access to Diverse Terpenoids

Rachid Chahboun[†]*, José Manuel Botubol-Ares,[¥] María Jesús DuránPeña, [¥]Fermín Jiménez, [†]Ramón Alvarez-Manzaneda[‡] and Enrique Alvarez-Manzaneda[†]

[†]Departamento de Química Orgánica, Facultad de Ciencias, Instituto de Biotecnología, Universidad de Granada, 18071 Granada, Spain

[¥]Departamento de Química Orgánica, Facultad de Ciencias, Campus Universitario Río San Pedro s/n, Torre Sur, 4ª planta, Universityof Cádiz, 11510, Puerto Real, Cádiz, Spain

[‡]Área de Química Orgánica, Departamento de Química y Física, Universidad de Almería, 04120 Almería, Spain

Corresponding Author

* Tel: (+34) 958 244022. E-mail: rachid@ugr.es

Table of Contents:

^{1}H and ^{13}C NMR spectra of compounds S2-S57

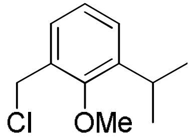

^{1}H NMR (400 MHz, $CDCl_3$) for **10j**

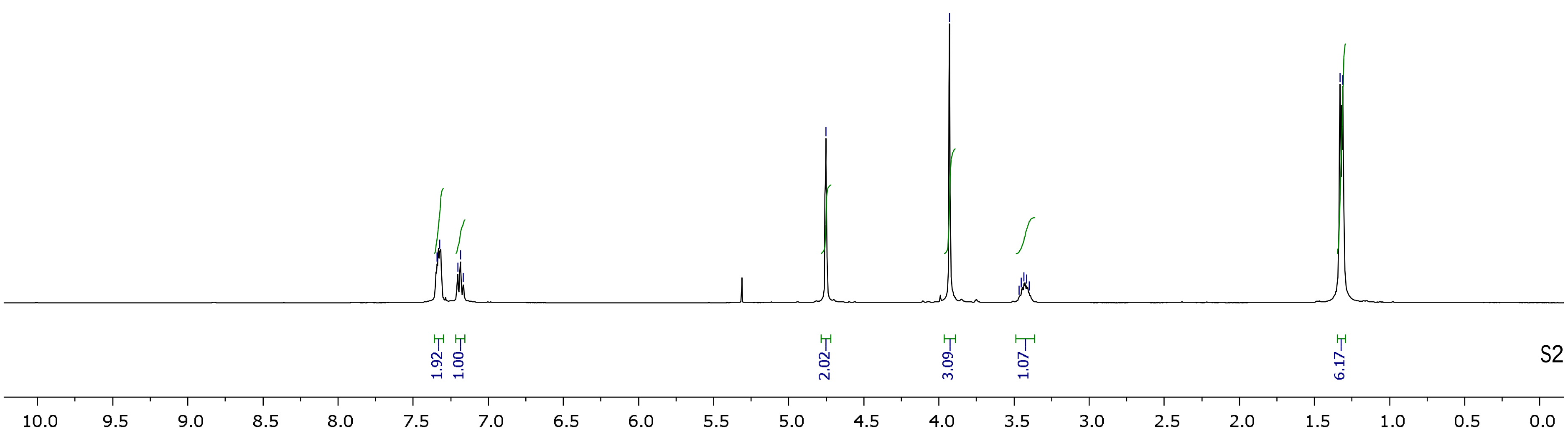

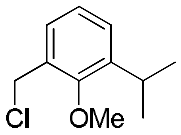

$^{13}C\{^{1}H\}$ NMR (125 MHz, $CDCl_3$) for **10j**

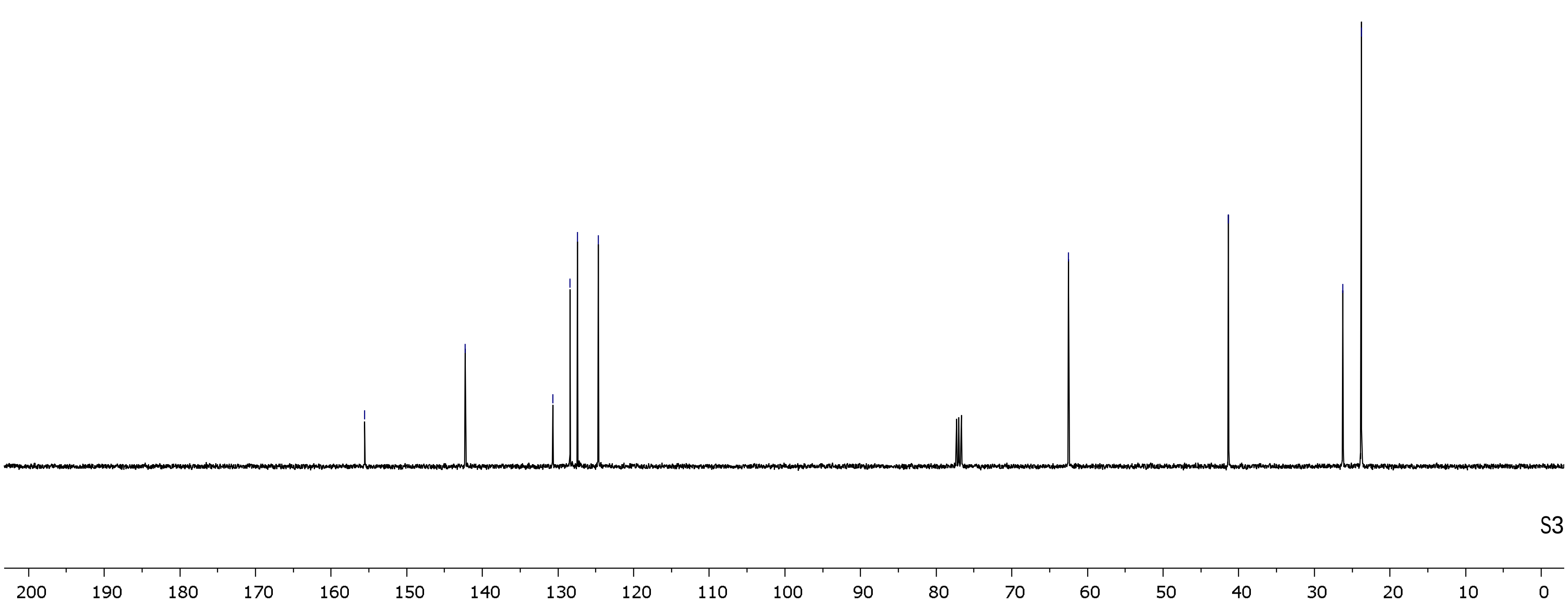

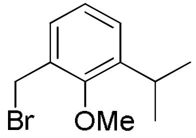

^{1}H NMR (400 MHz, CD_3COCD_3) for **10k**

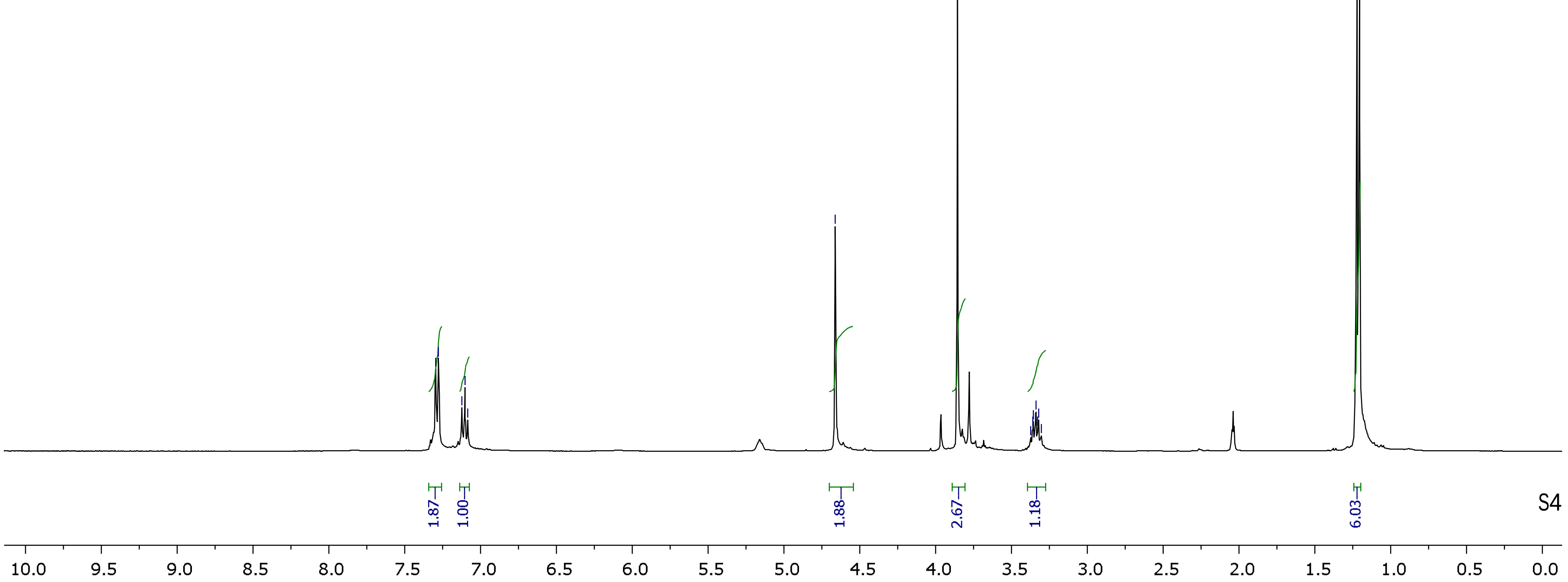

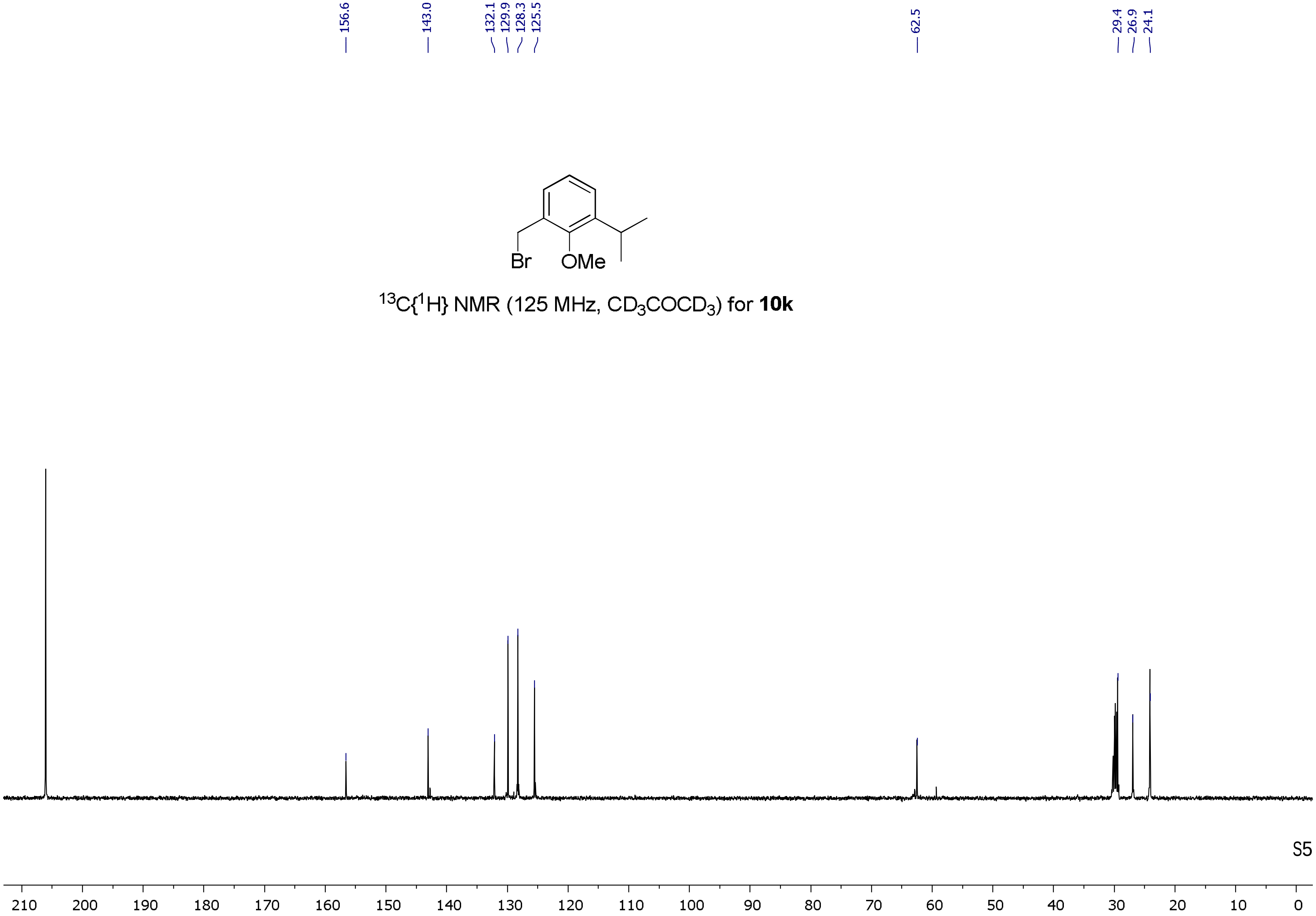

$^{13}C\{^{1}H\}$ NMR (125 MHz, CD_3COCD_3) for **10k**

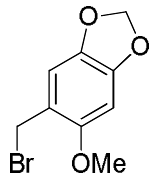

^{1}H NMR (500 MHz, CD_3COCD_3) for **10l**

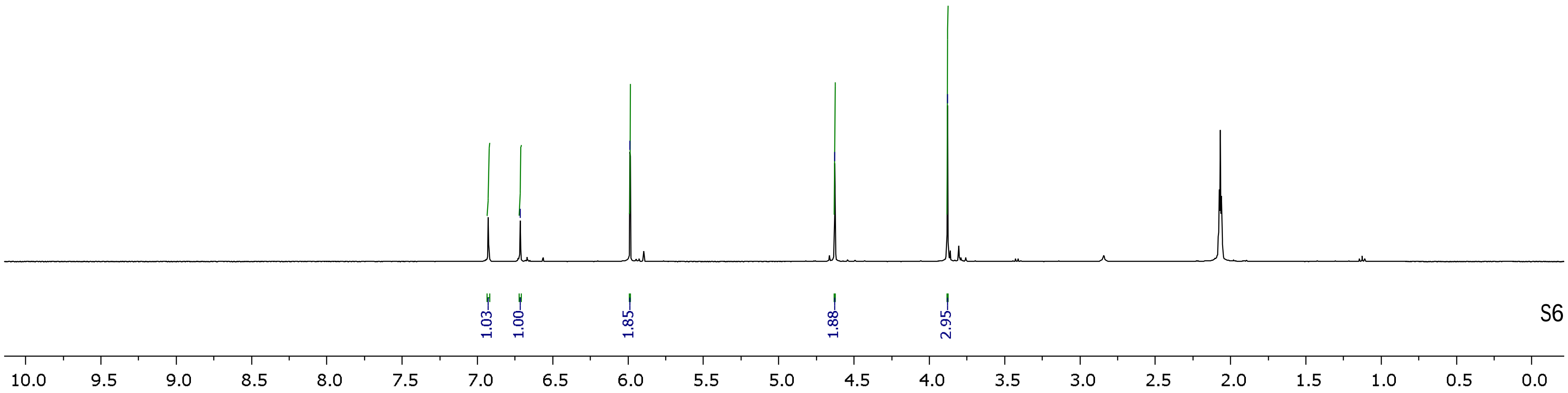

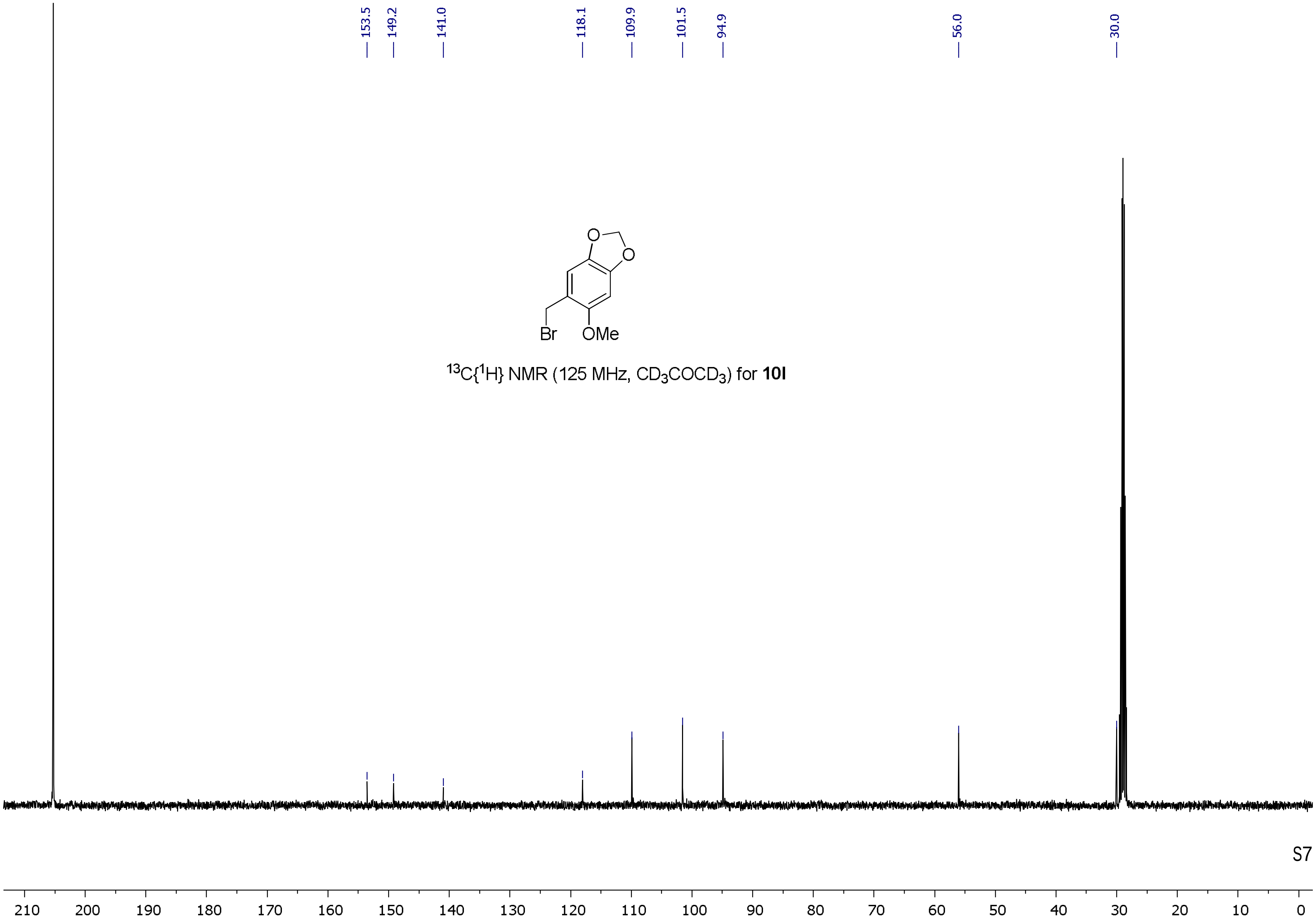

$^{13}C\{^1H\}$ NMR (125 MHz, CD_3COCD_3) for **10l**

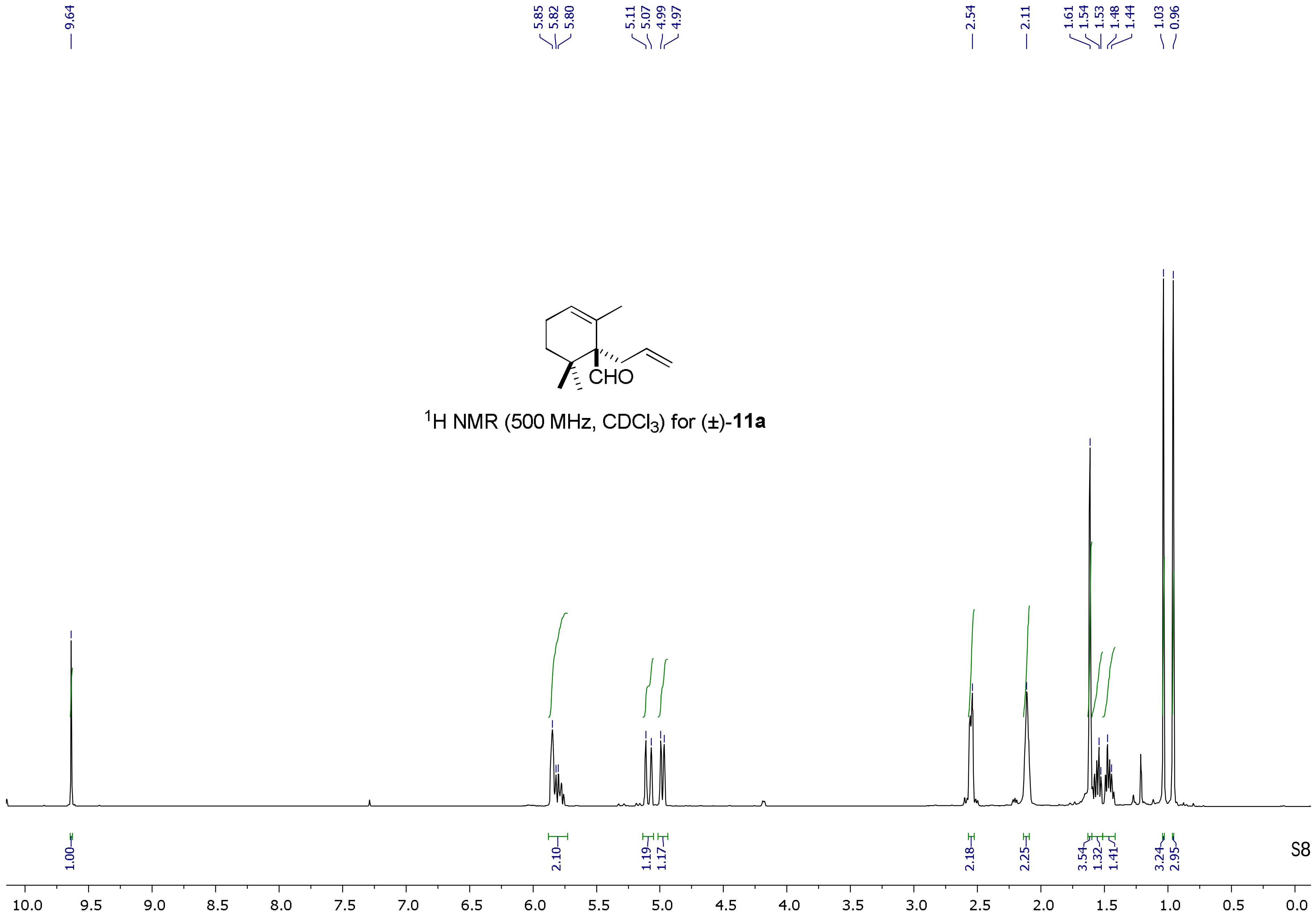
9.64
5.85
5.82
5.80
5.11
5.07
4.99
4.97
2.54
2.11
1.61
1.54
1.53
1.48
1.44
1.03
0.96
CHO
1H NMR (500 MHz, CDCl3) for (±)-11a
1.00
2.10
1.19
1.17
2.18
2.25
3.54
1.32
1.41
3.24
2.95
10.0
9.5
9.0
8.5
8.0
7.5
7.0
6.5
6.0
5.5
5.0
4.5
4.0
3.5
3.0
2.5
2.0
1.5
1.0
0.5
0.0

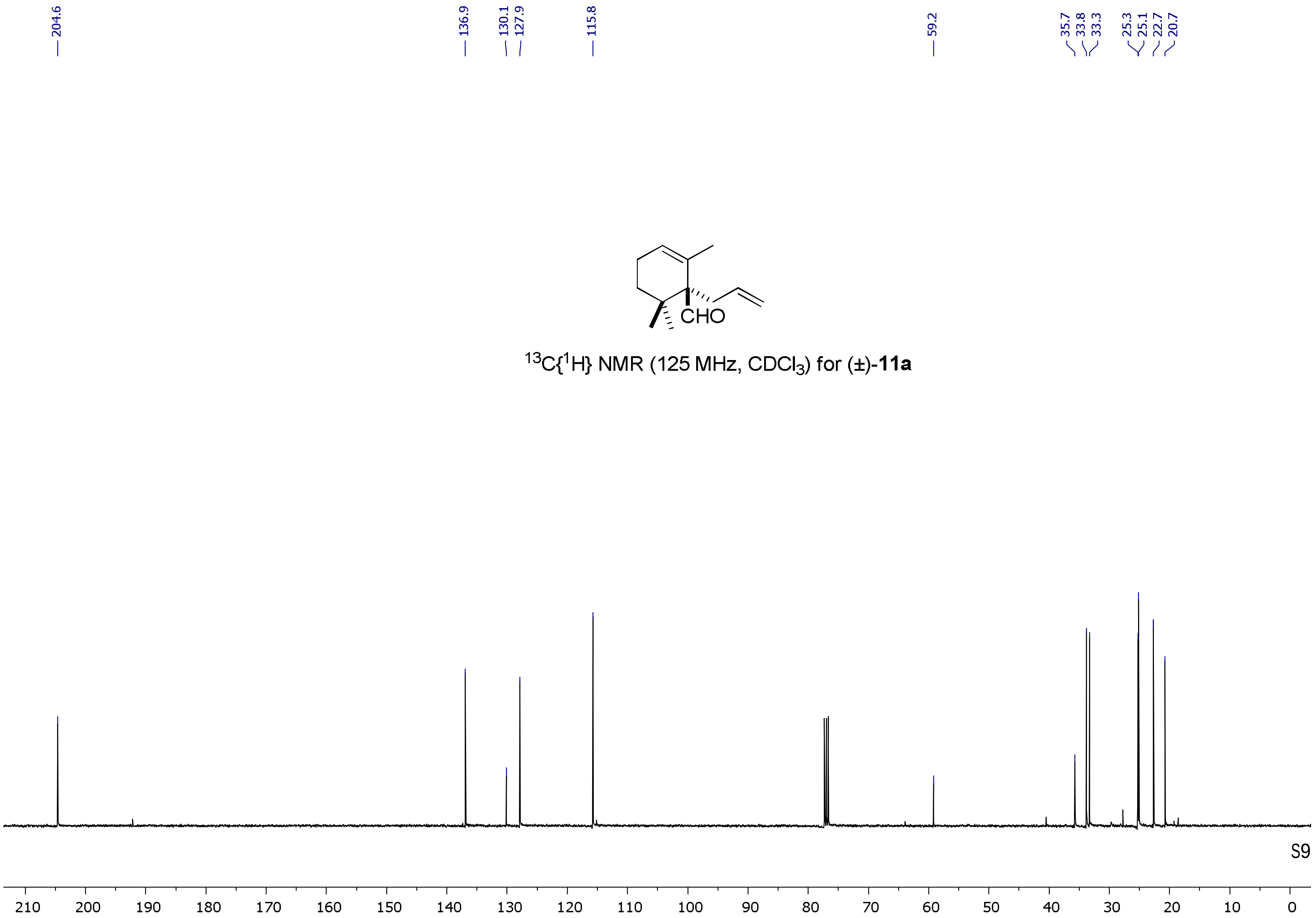

$^{13}C\{^{1}H\}$ NMR (125 MHz, $CDCl_3$) for (±)-**11a**

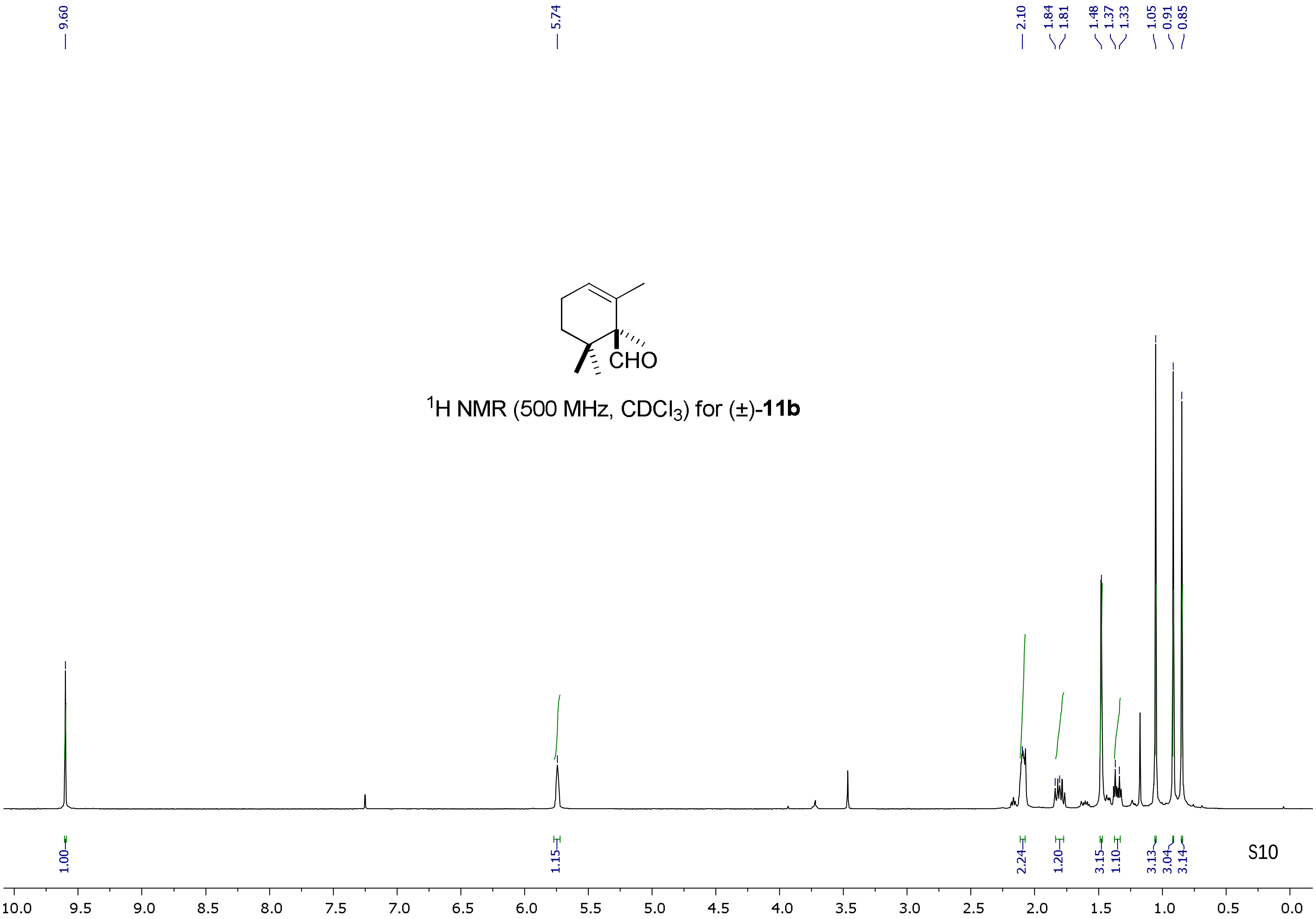

^{1}H NMR (500 MHz, $CDCl_3$) for (±)-**11b**

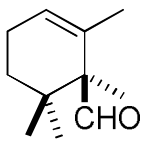

$^{13}C\{^{1}H\}$ NMR (125 MHz, $CDCl_3$) for (±)-**11b**

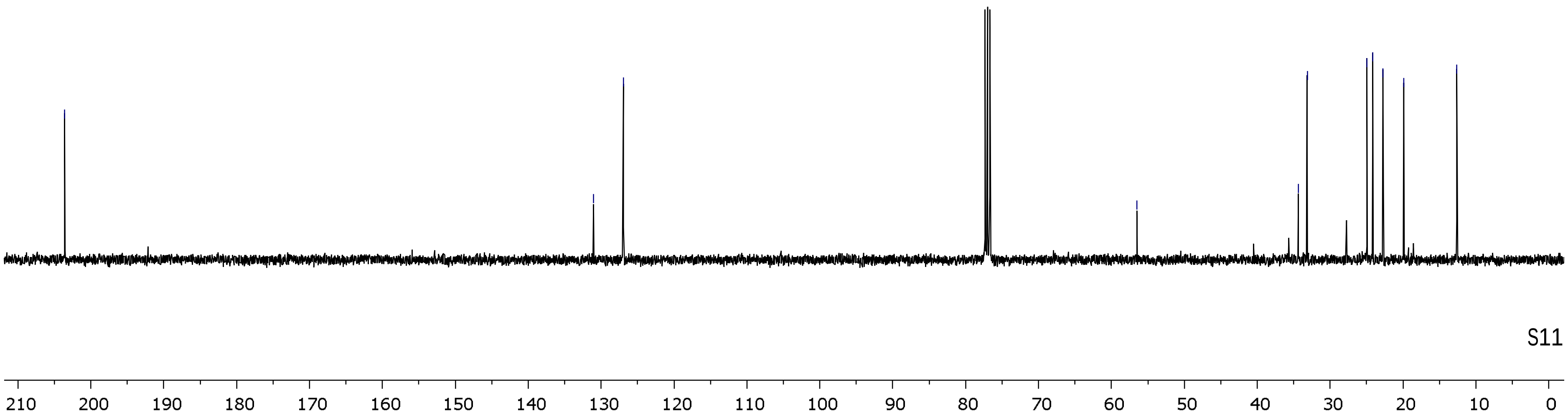

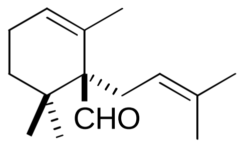

^{1}H NMR (500 MHz, $CDCl_3$) for (±)-**11c**

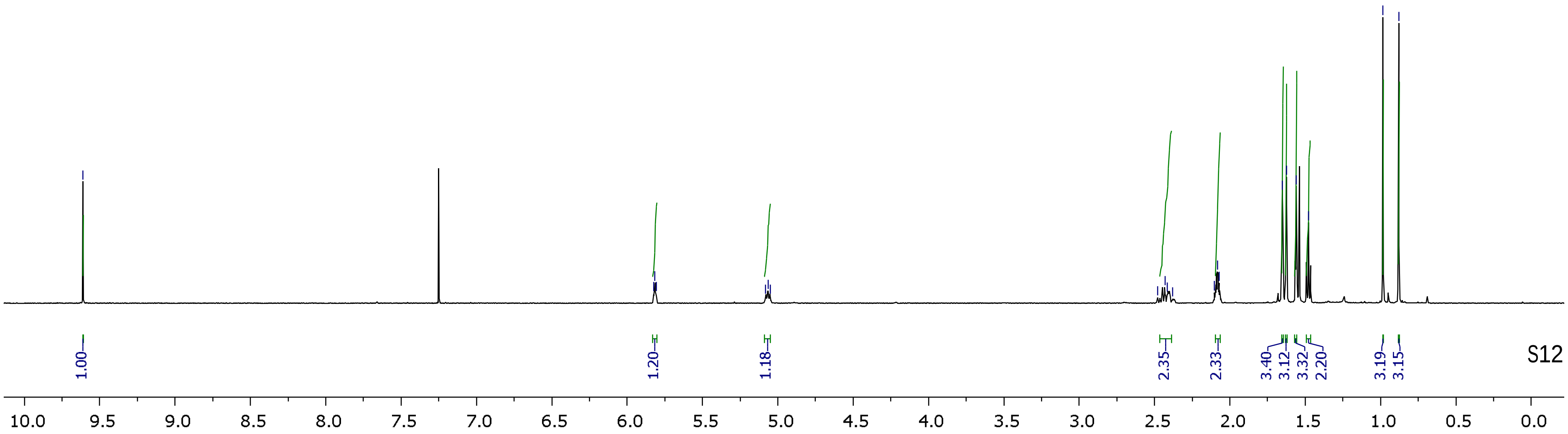

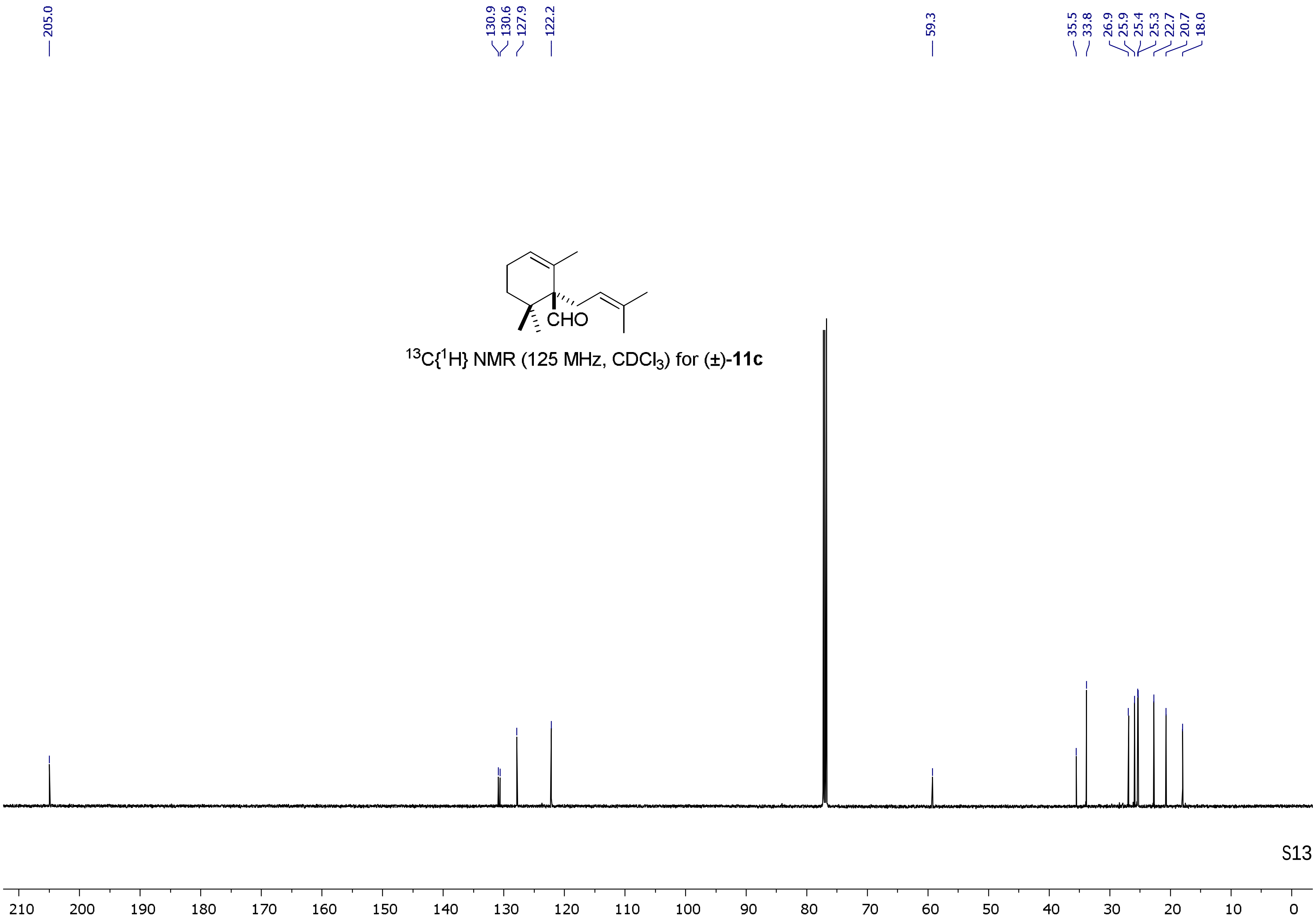

$^{13}C\{^1H\}$ NMR (125 MHz, $CDCl_3$) for (±)-**11c**

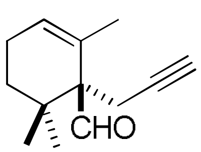

^{1}H NMR (500 MHz, $CDCl_3$) for (±)-**11d**

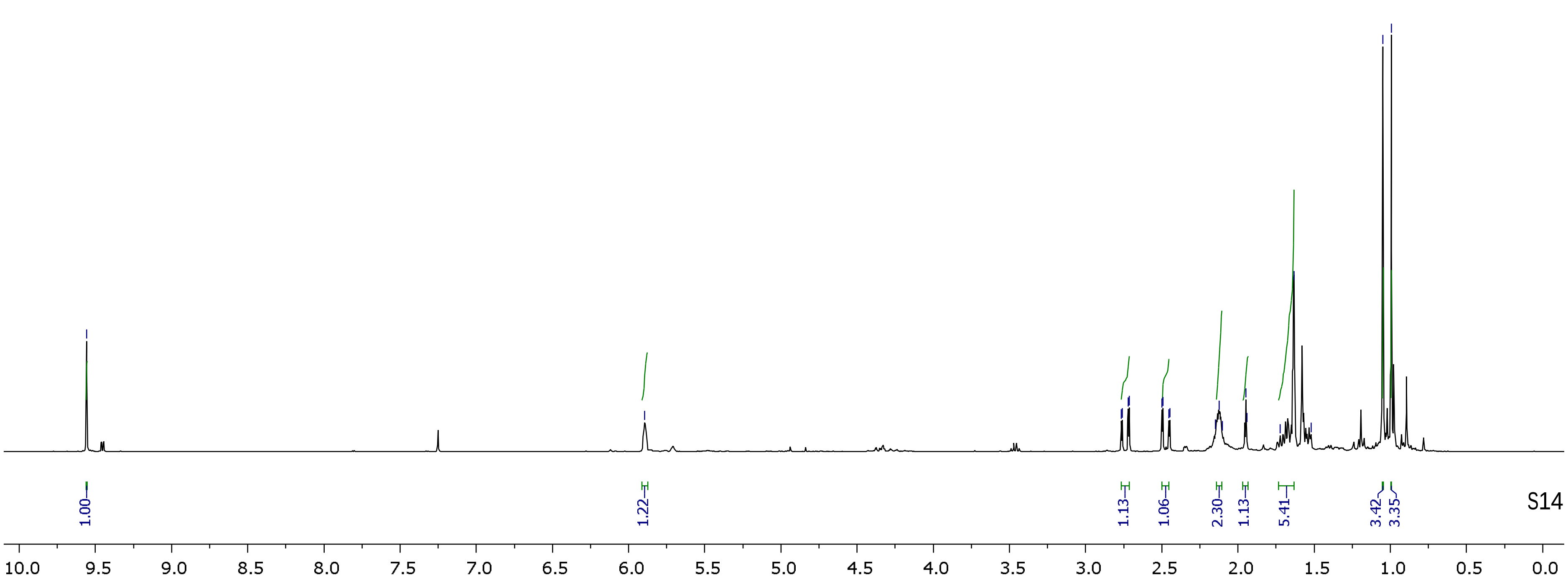

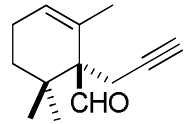

$^{13}C\{^{1}H\}$ NMR (125 MHz, $CDCl_3$) for (±)-**11d**

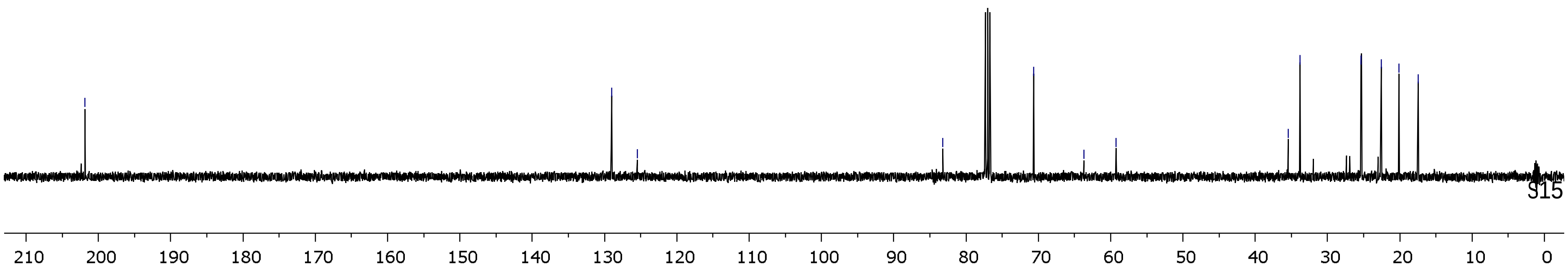

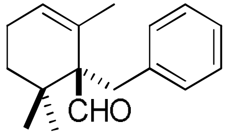

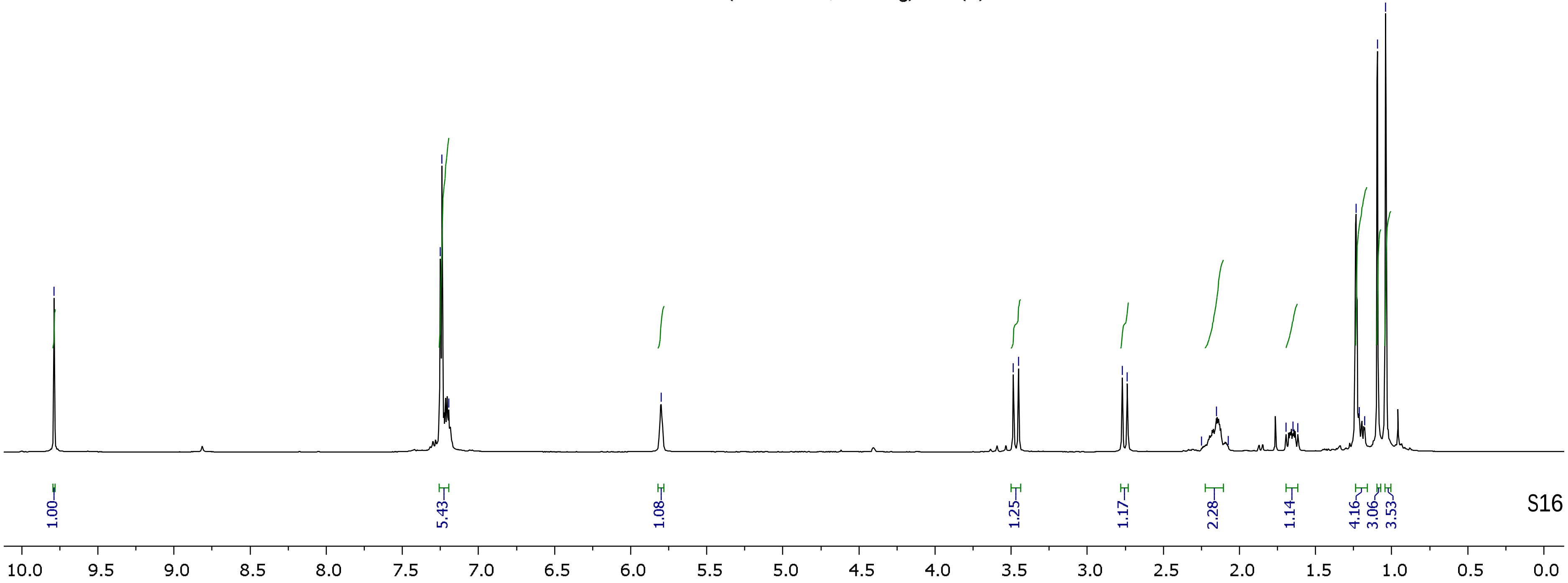

^{1}H NMR (400 MHz, $CDCl_3$) for (±)-**11e**

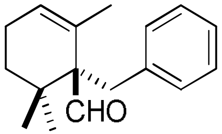

$^{13}C\{^{1}H\}$ NMR (100 MHz, $CDCl_3$) for (±)-**11e**

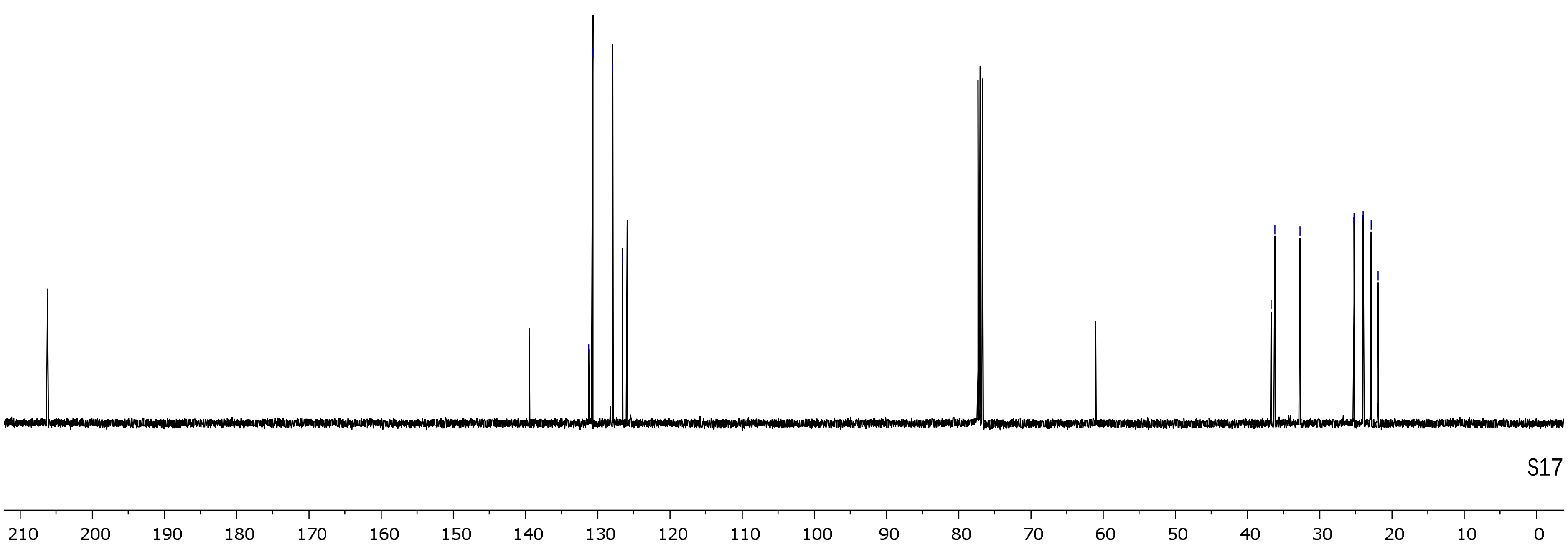

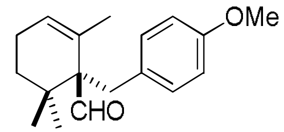

^1H NMR (500 MHz, $CDCl_3$) for (±)-**11f**

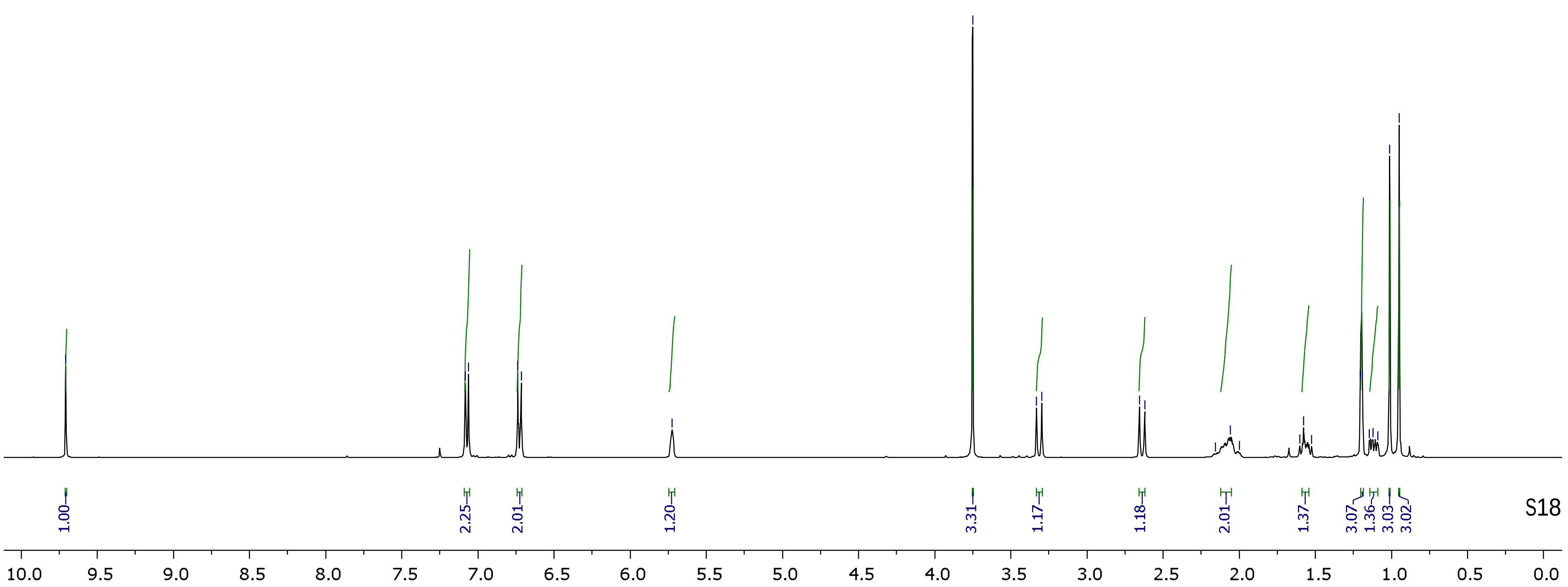

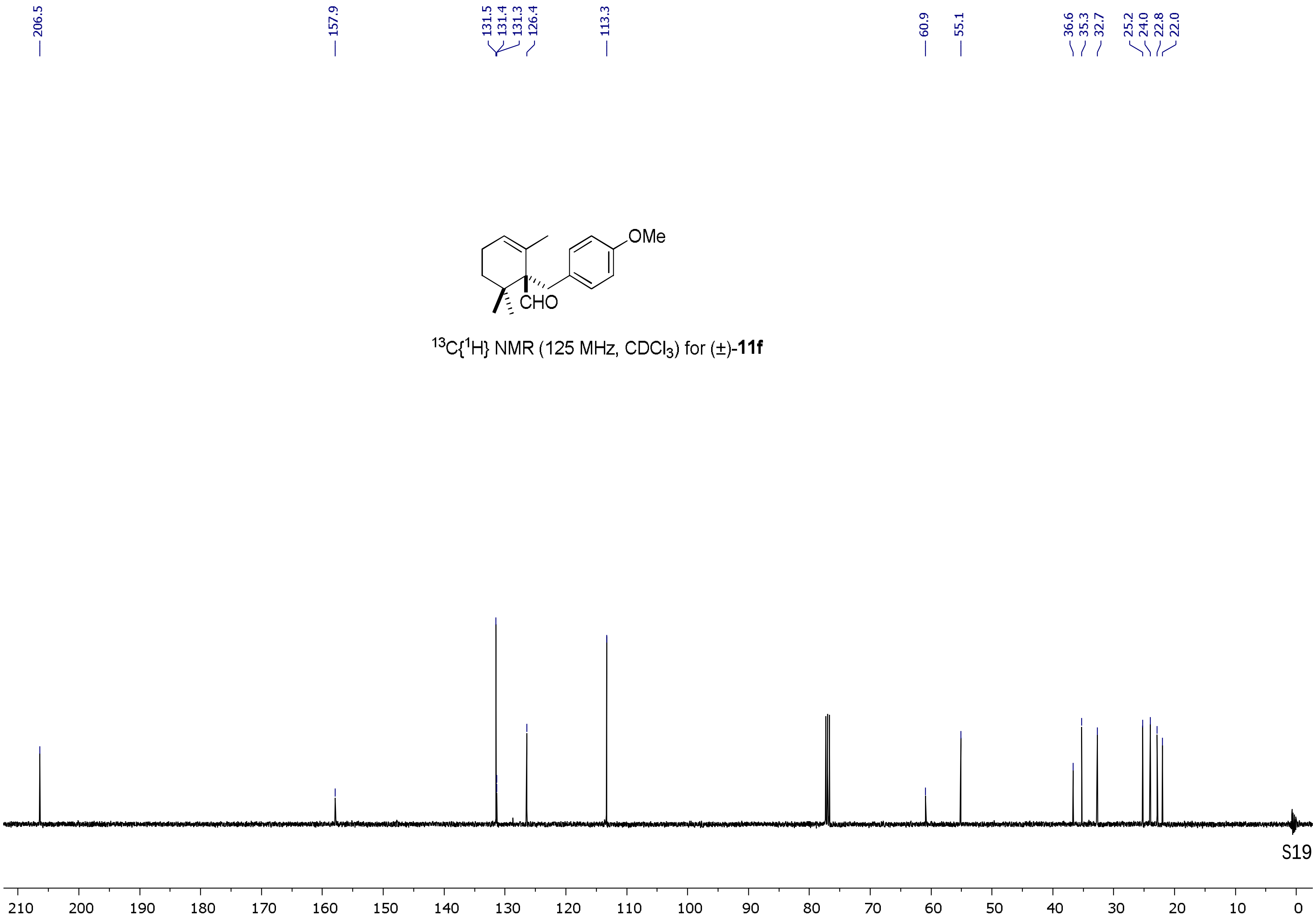

$^{13}C\{^1H\}$ NMR (125 MHz, $CDCl_3$) for (±)-**11f**

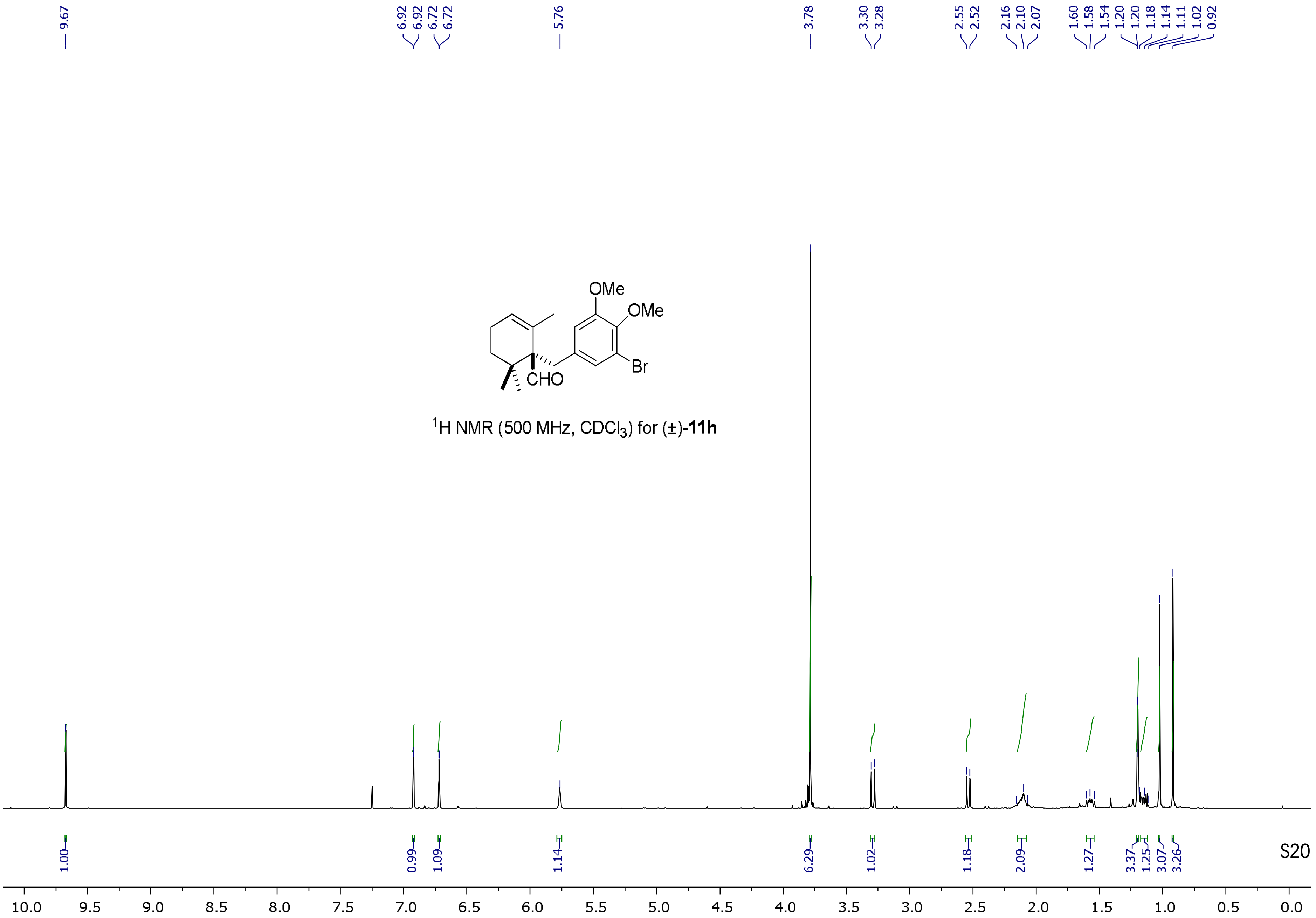

^{1}H NMR (500 MHz, $CDCl_3$) for (±)-**11h**

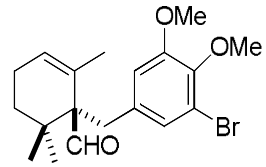

$^{13}C\{^{1}H\}$ NMR (125 MHz, $CDCl_3$) for (±)-**11h**

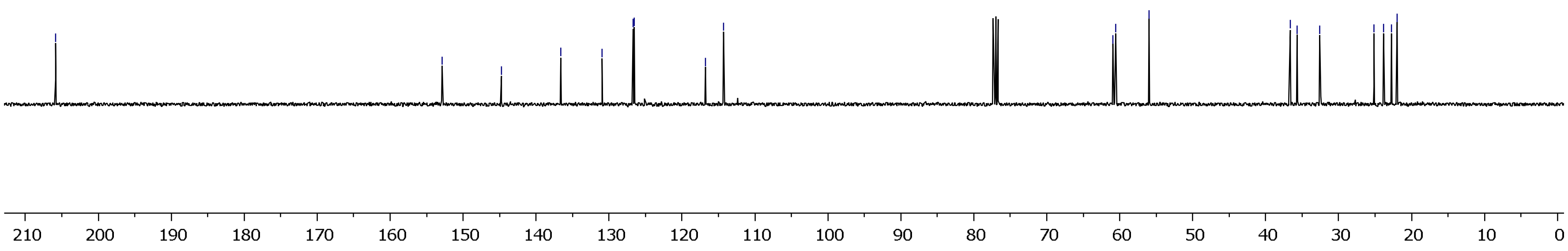

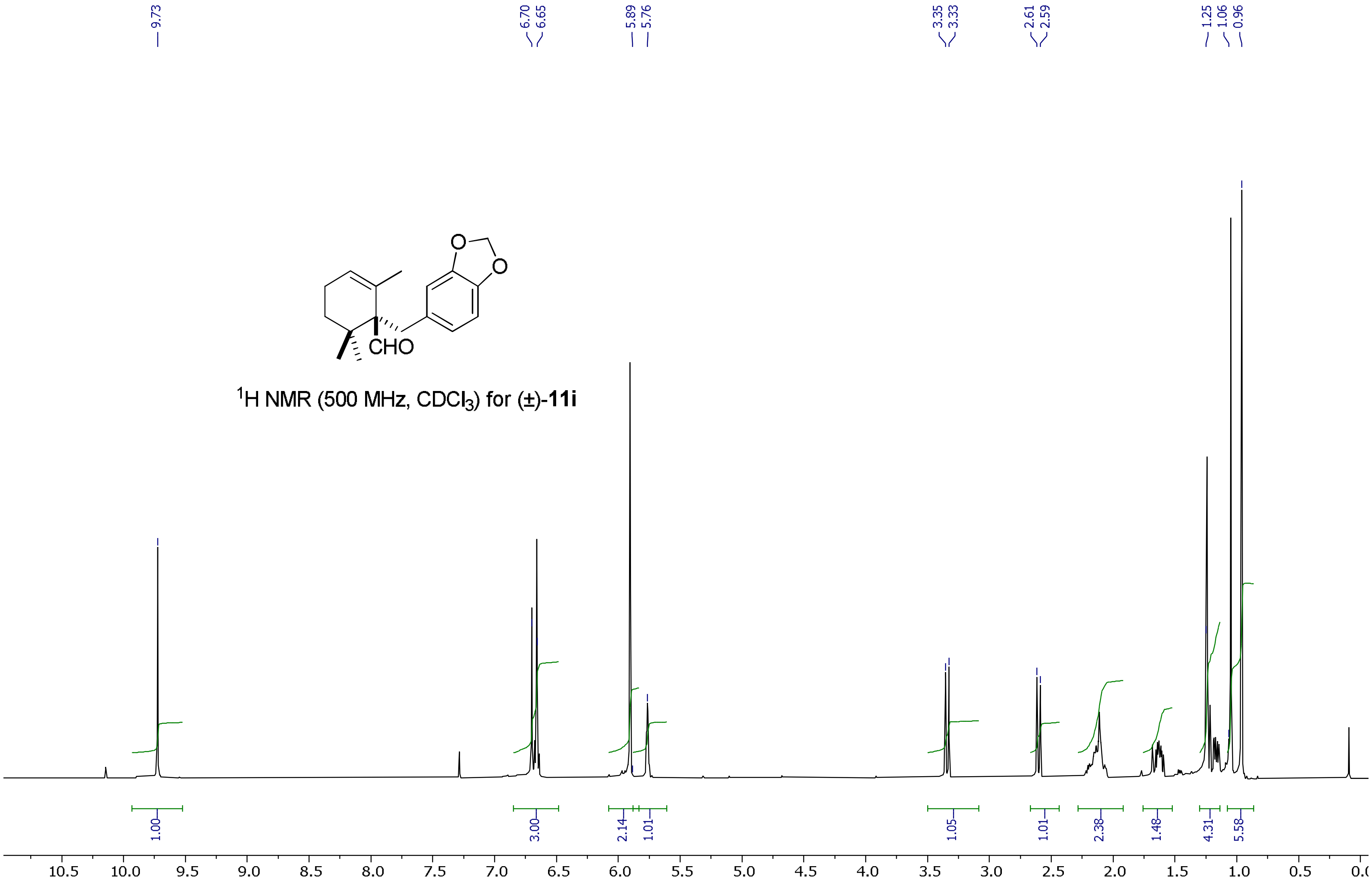

^{1}H NMR (500 MHz, $CDCl_3$) for (±)-**11i**

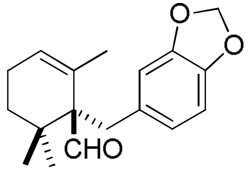

$^{13}C\{^{1}H\}$ NMR (100 MHz, $CDCl_3$) for (±)-**11i**

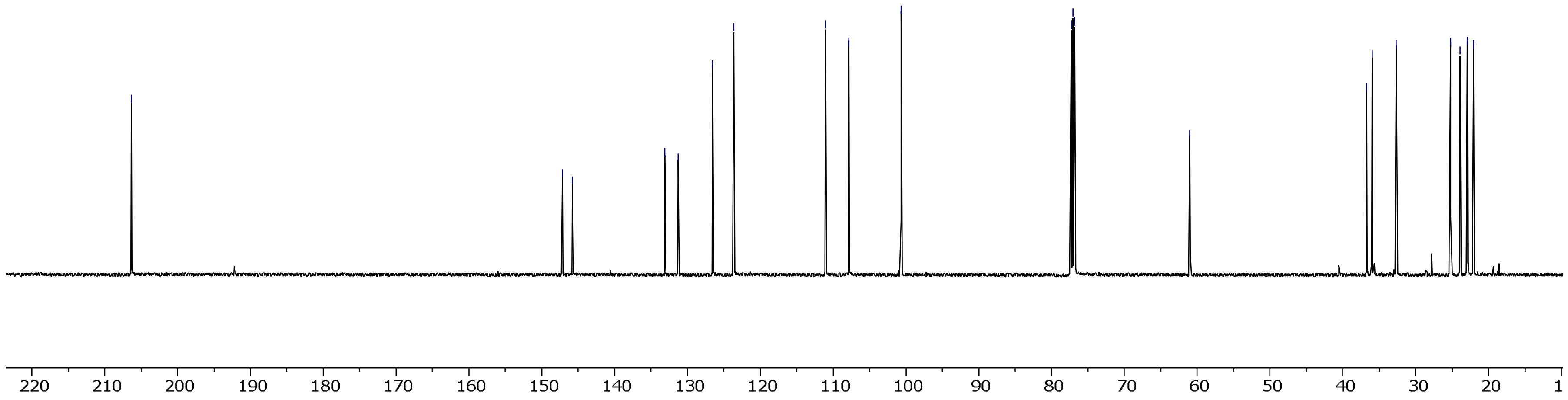

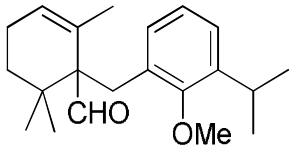

^{1}H NMR (500 MHz, $CDCl_3$) for (±)-**11j**

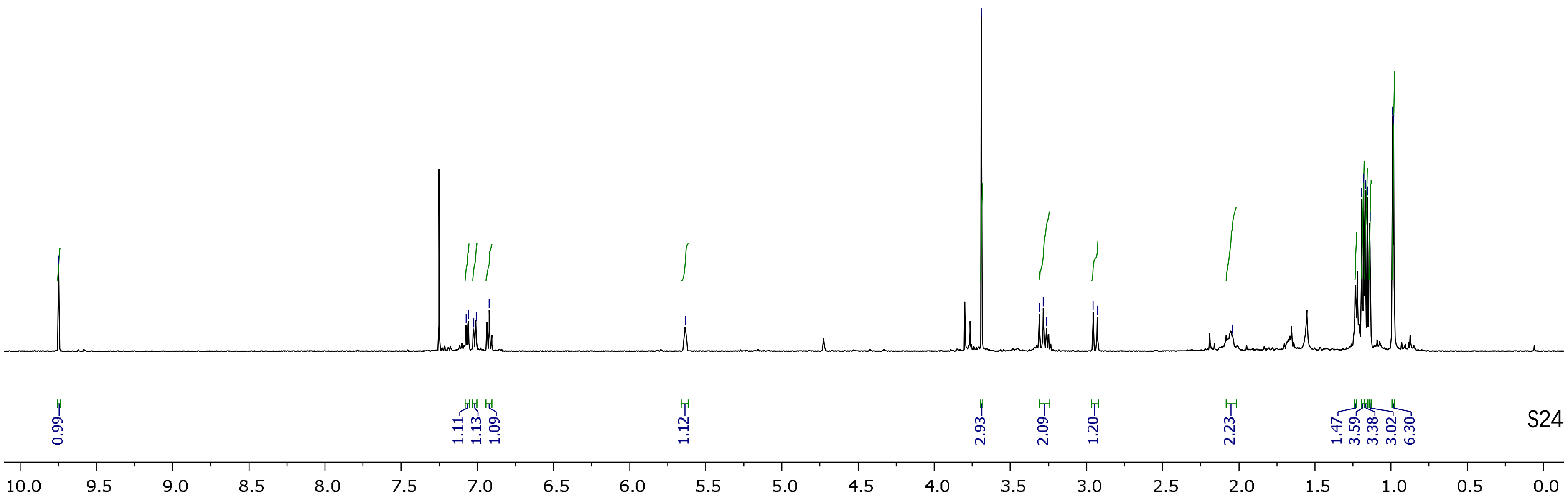

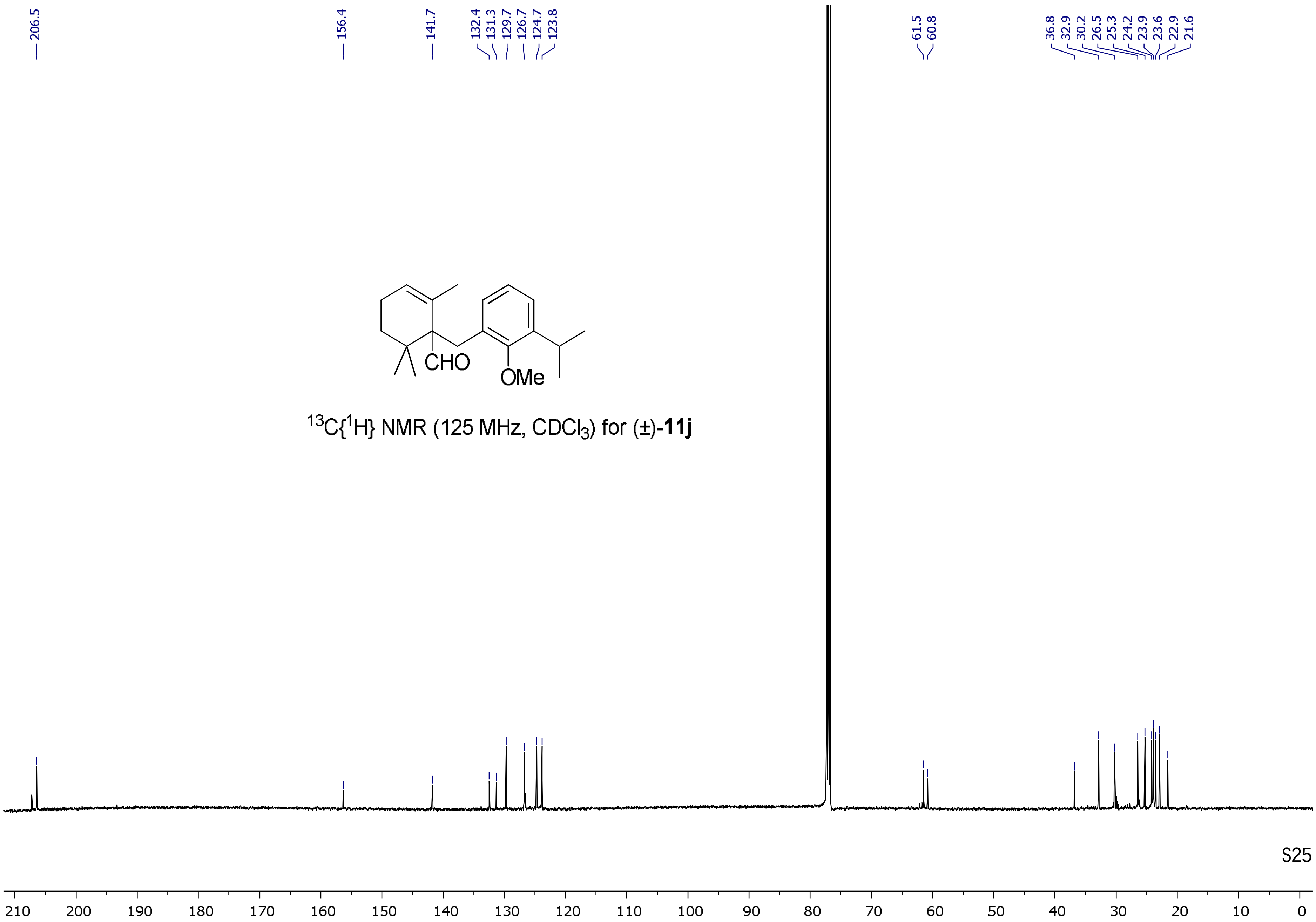

206.5
156.4
141.7
132.4
131.3
129.7
126.7
124.7
123.8
61.5
60.8
36.8
32.9
30.2
26.5
25.3
24.2
23.9
23.6
22.9
21.6
CHO
OMe
$^{13}C\{^1H\}$ NMR (125 MHz, $CDCl_3$) for (±)-**11j**
210 200 190 180 170 160 150 140 130 120 110 100 90 80 70 60 50 40 30 20 10 0

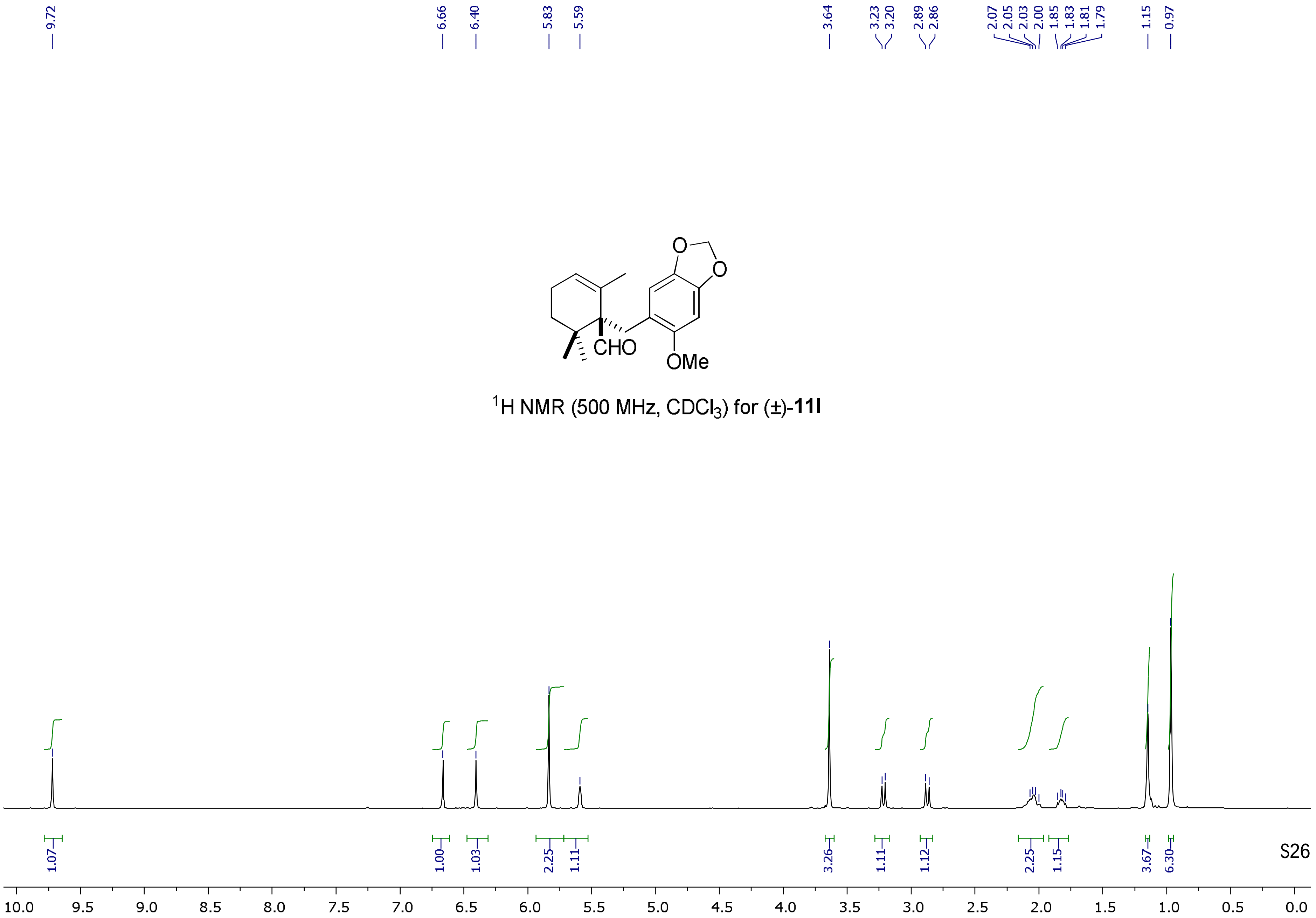

^{1}H NMR (500 MHz, $CDCl_3$) for (±)-**11l**

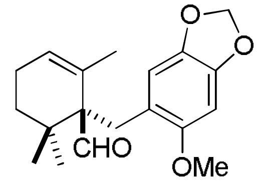

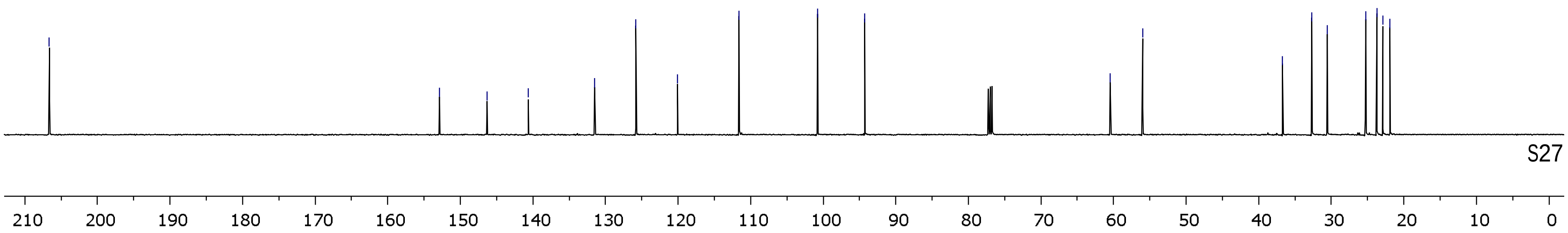

$^{13}C\{^{1}H\}$ NMR (125 MHz, $CDCl_3$) for (±)-**11l**

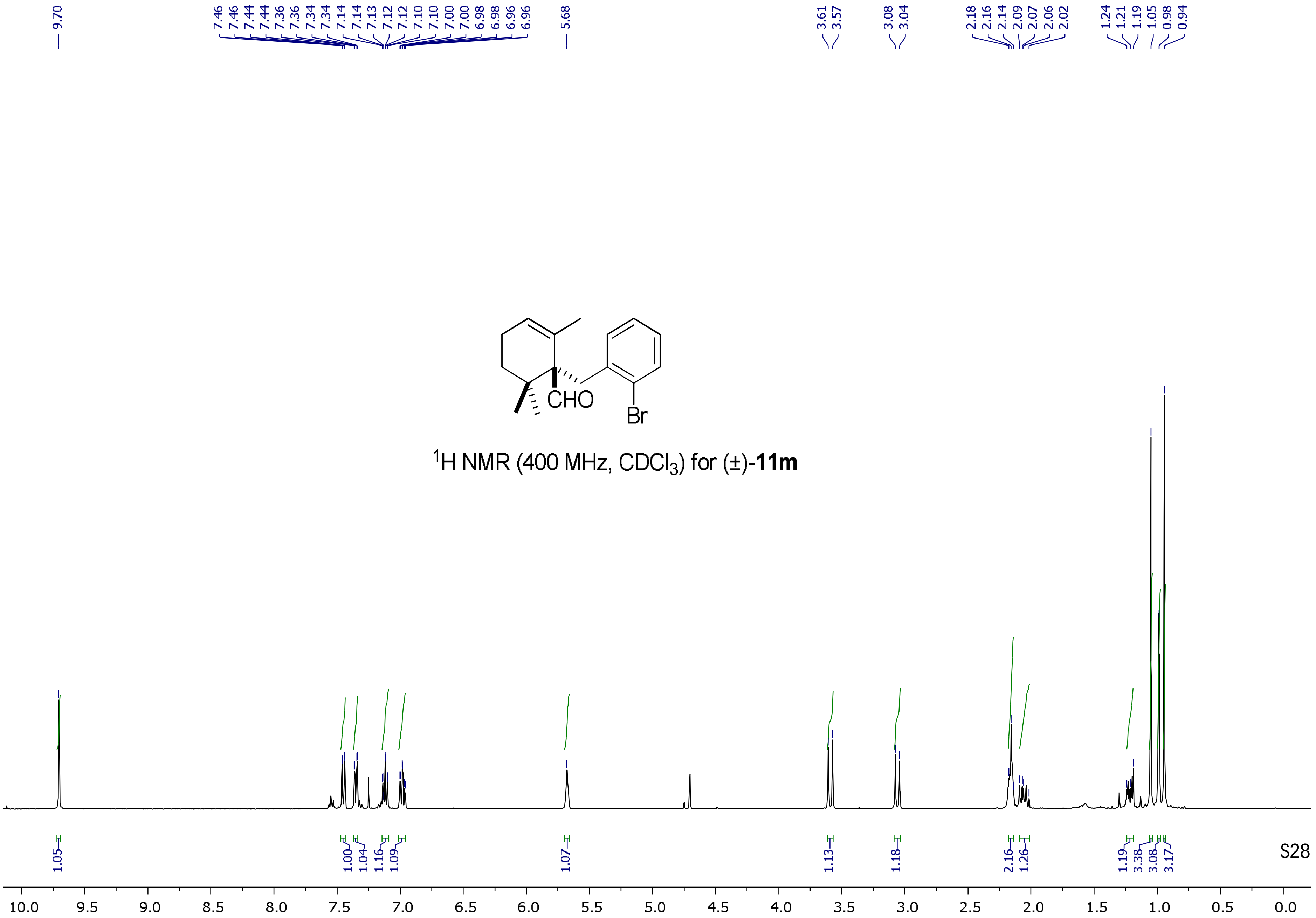

^{1}H NMR (400 MHz, $CDCl_3$) for (±)-**11m**

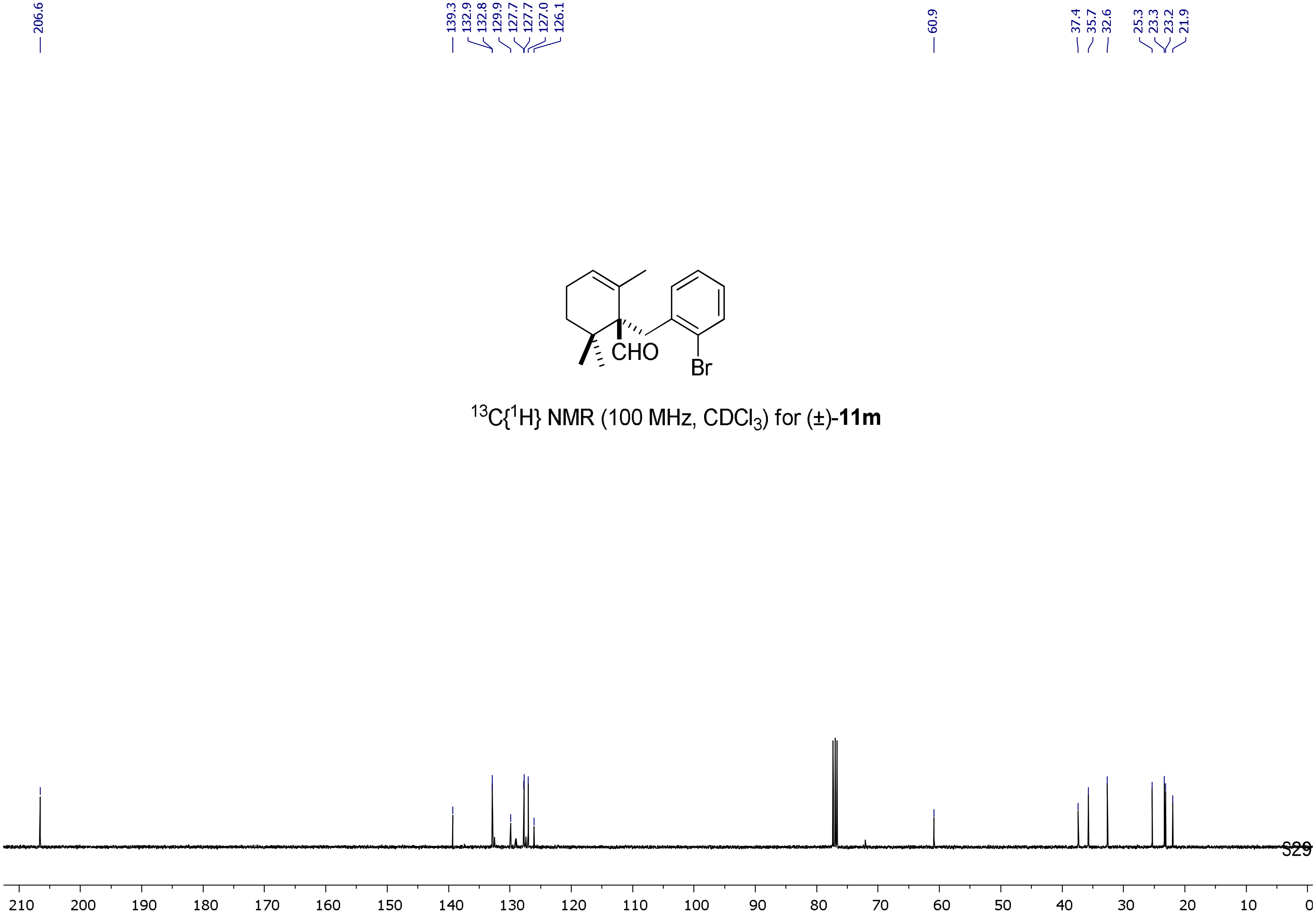

$^{13}C\{^{1}H\}$ NMR (100 MHz, $CDCl_3$) for (±)-**11m**

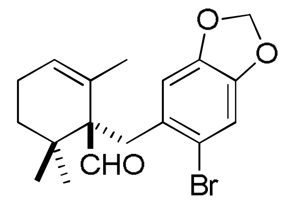

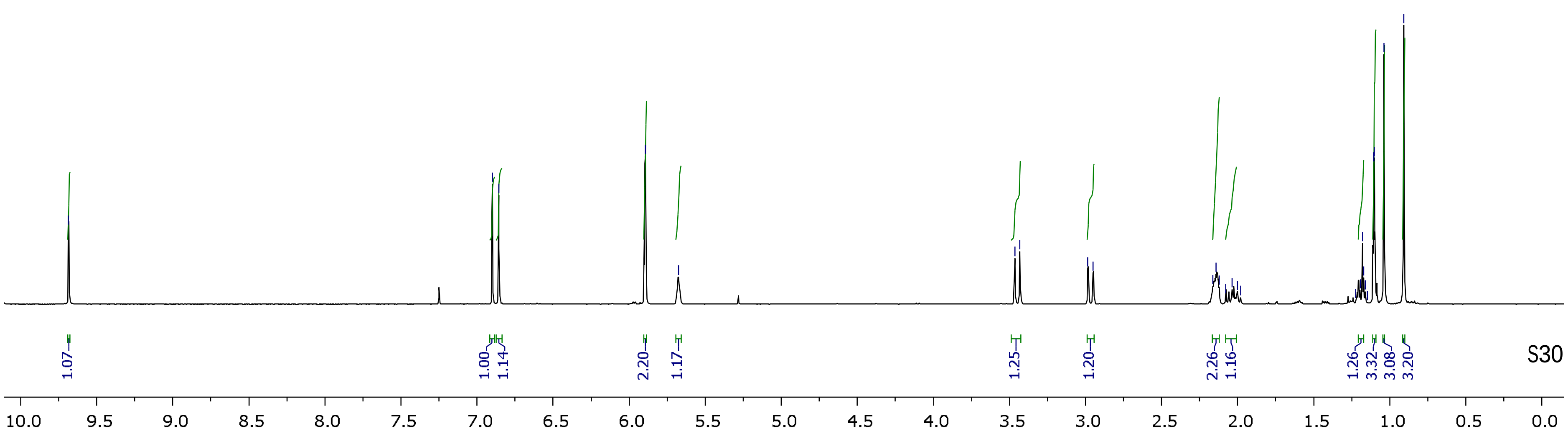

^{1}H NMR (400 MHz, $CDCl_3$) for (±)-**11n**

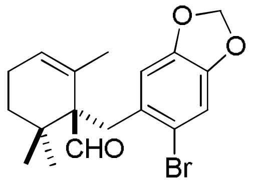

$^{13}C\{^1H\}$ NMR (100 MHz, $CDCl_3$) for (±)-**11n**

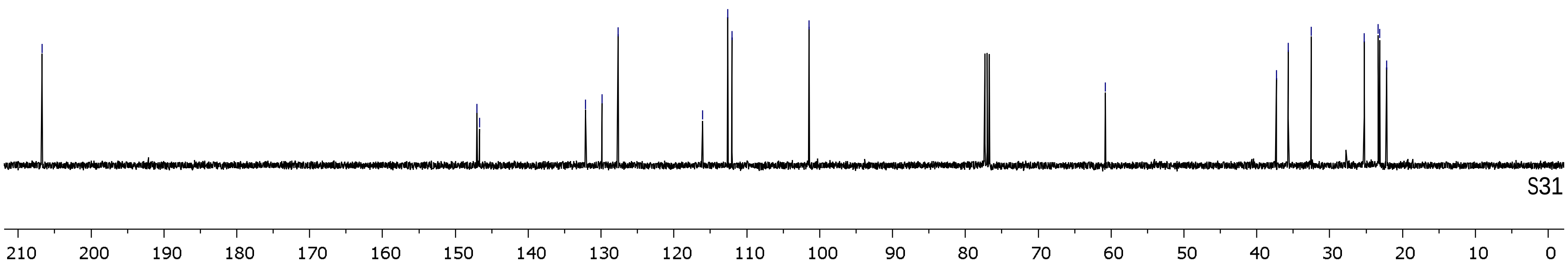

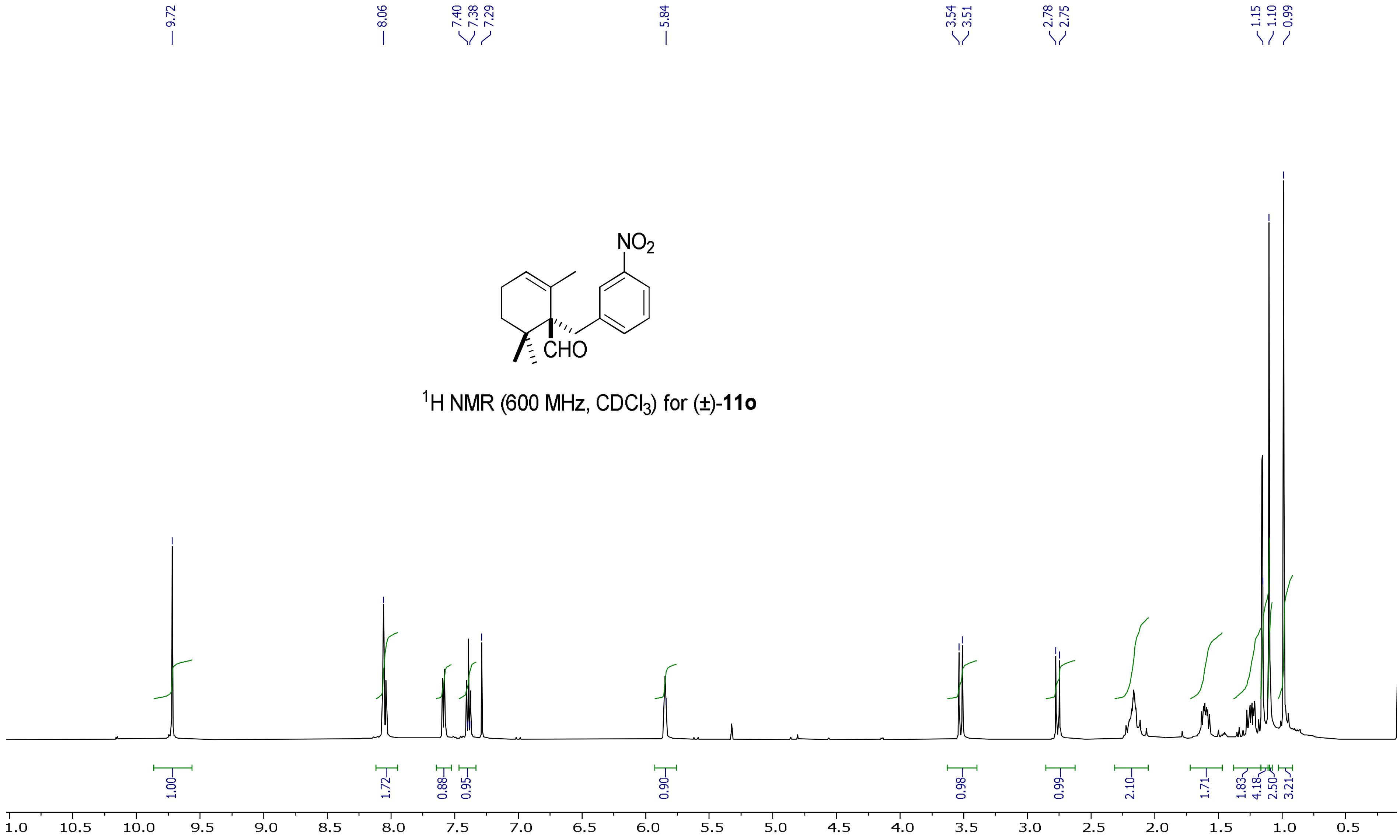

^{1}H NMR (600 MHz, $CDCl_3$) for (±)-**11o**

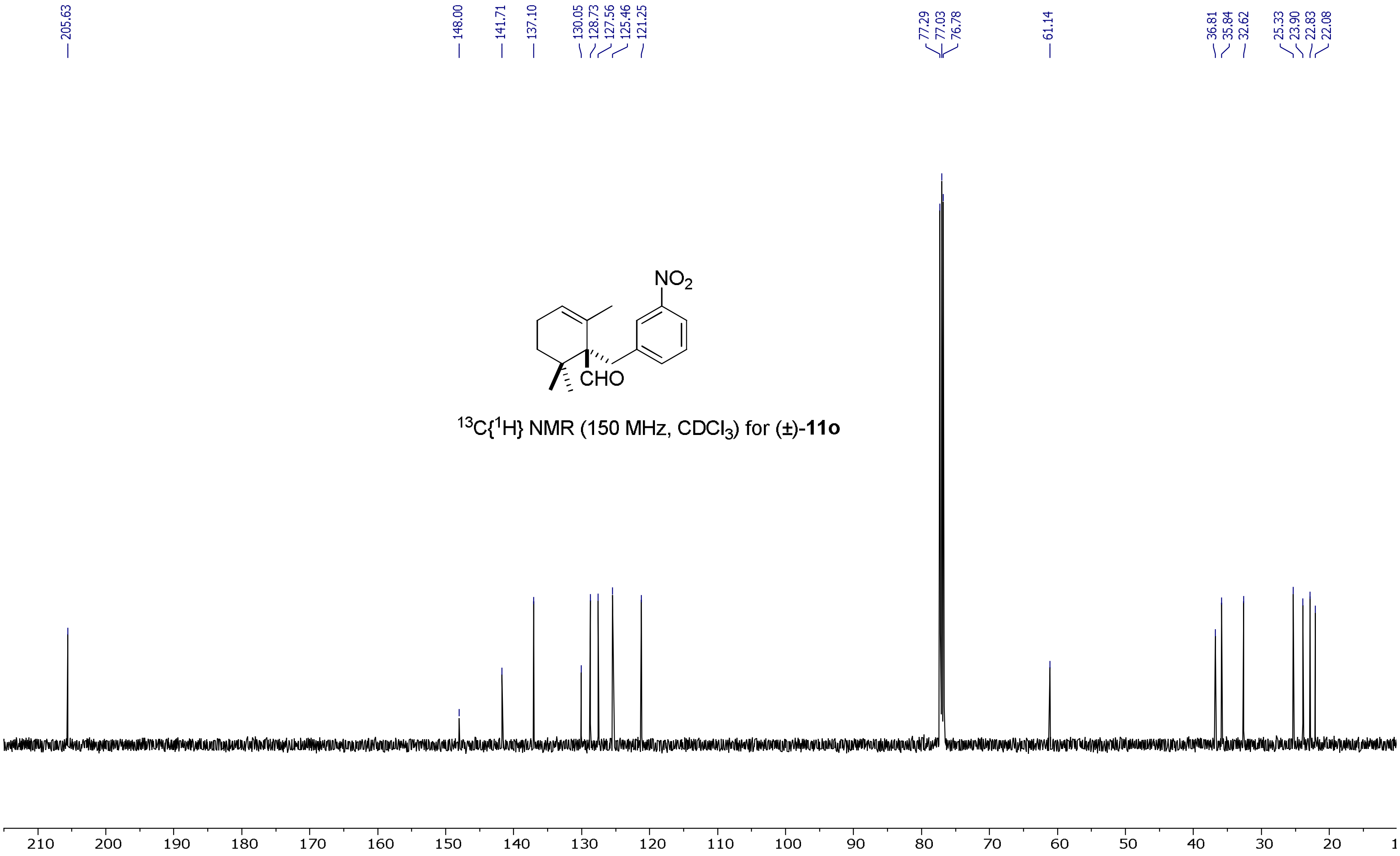

$^{13}C\{^{1}H\}$ NMR (150 MHz, $CDCl_3$) for (±)-**11o**

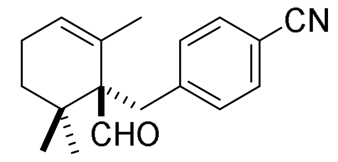

^{1}H NMR (500 MHz, $CDCl_3$) for (±)-**11p**

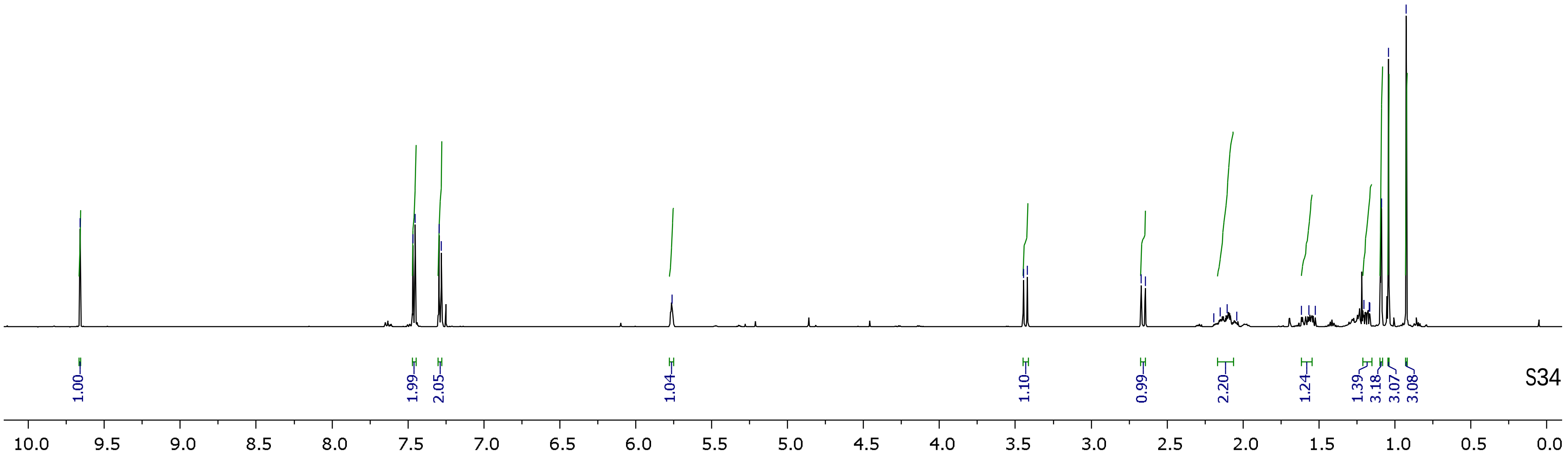

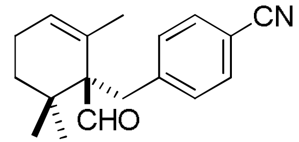

$^{13}C\{^1H\}$ NMR (125 MHz, $CDCl_3$) for (±)-**11p**

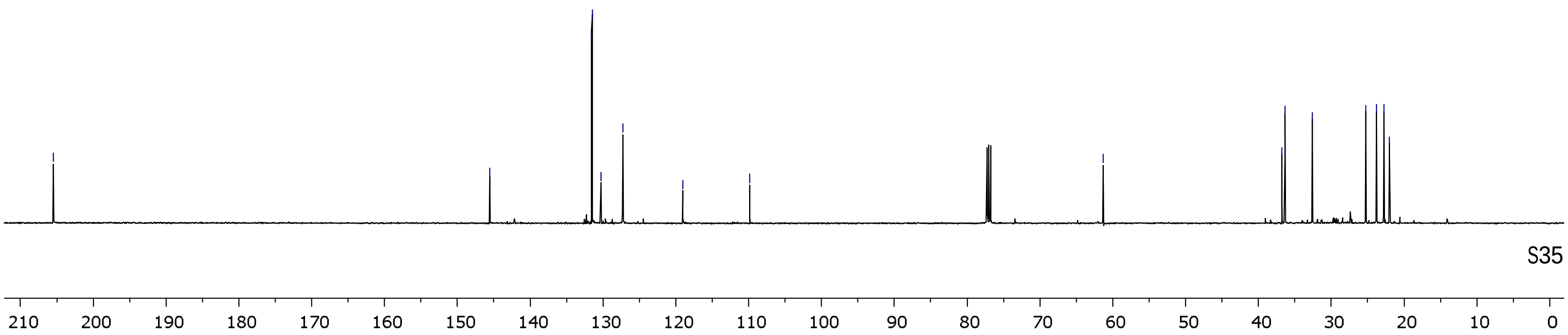

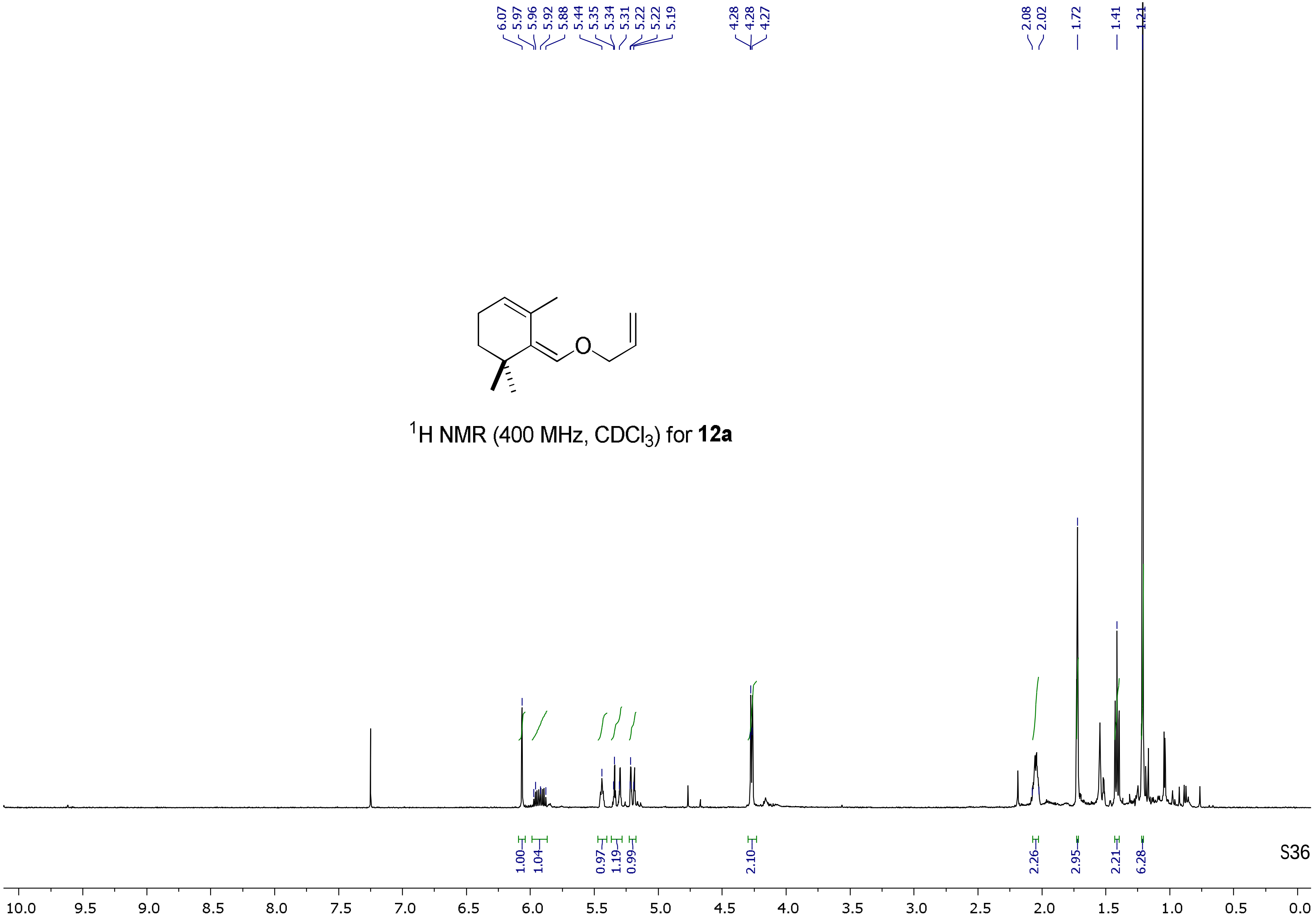

^{1}H NMR (400 MHz, $CDCl_3$) for **12a**

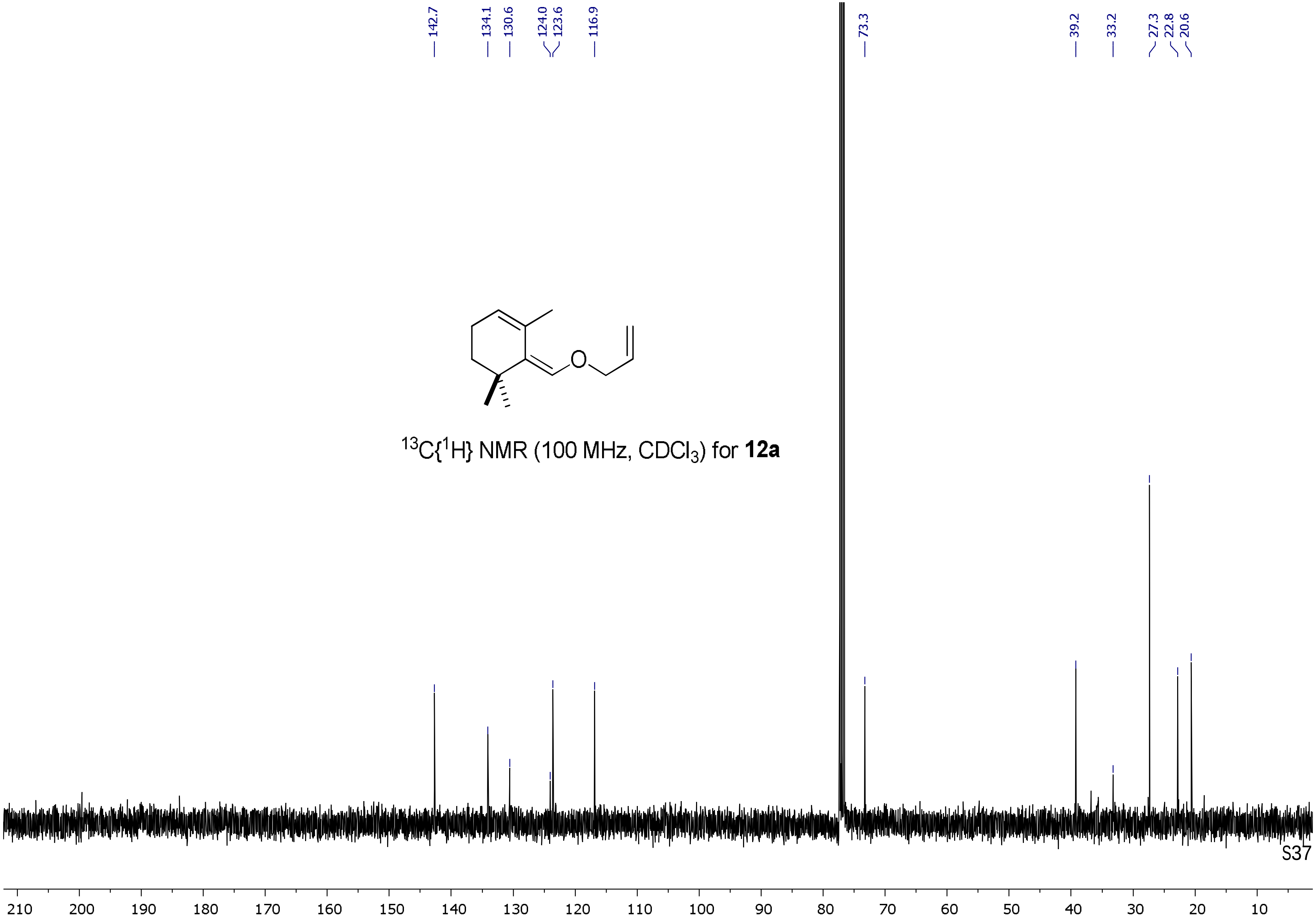
142.7
134.1
130.6
124.0
123.6
116.9
73.3
39.2
33.2
27.3
22.8
20.6
$^{13}C\{^{1}H\}$ NMR (100 MHz, $CDCl_3$) for **12a**
210 200 190 180 170 160 150 140 130 120 110 100 90 80 70 60 50 40 30 20 10

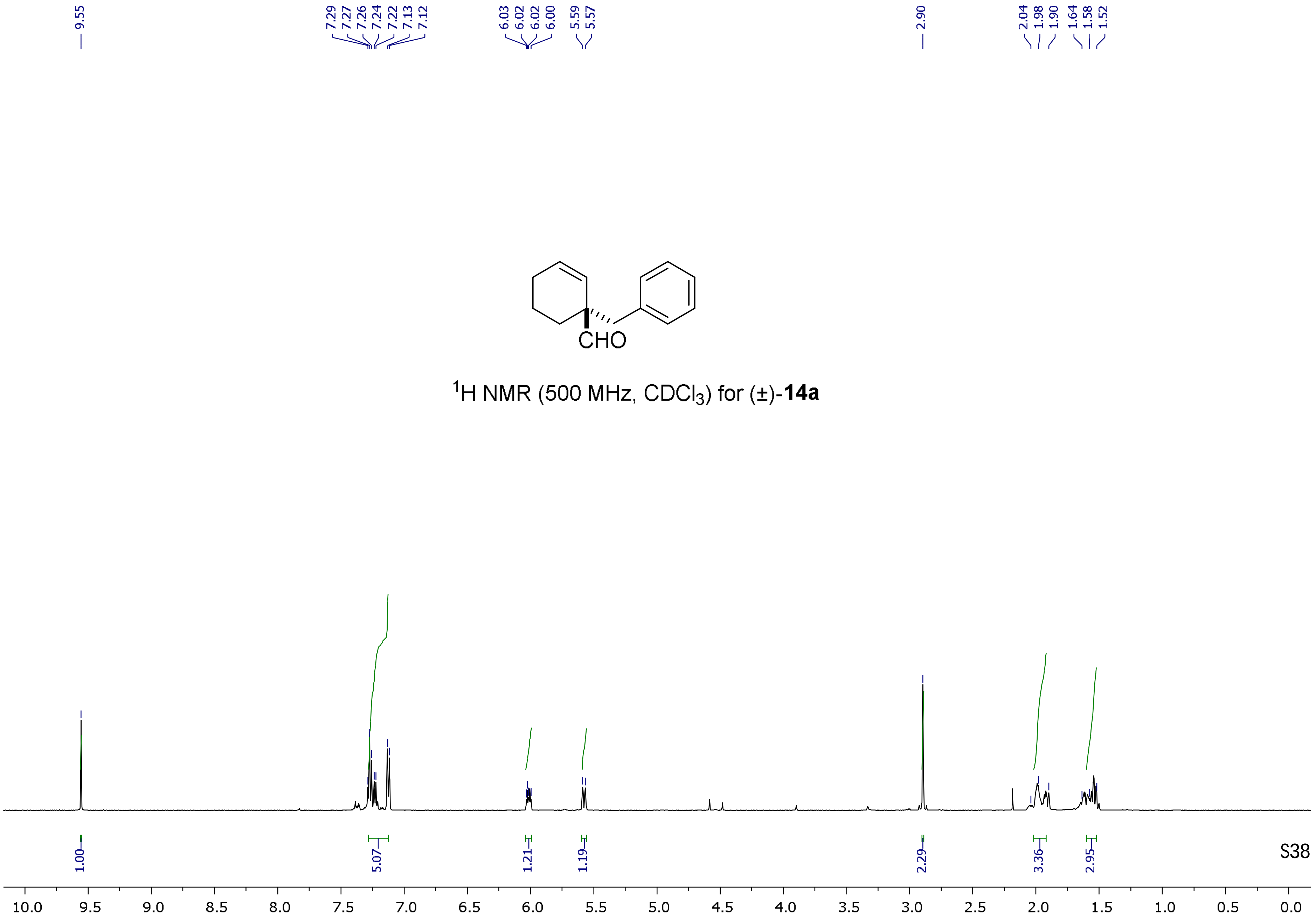

^1H NMR (500 MHz, $CDCl_3$) for (±)-**14a**

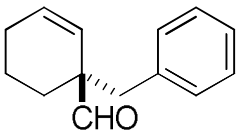

$^{13}C\{^{1}H\}$ NMR (125 MHz, $CDCl_3$) for (±)-**14a**

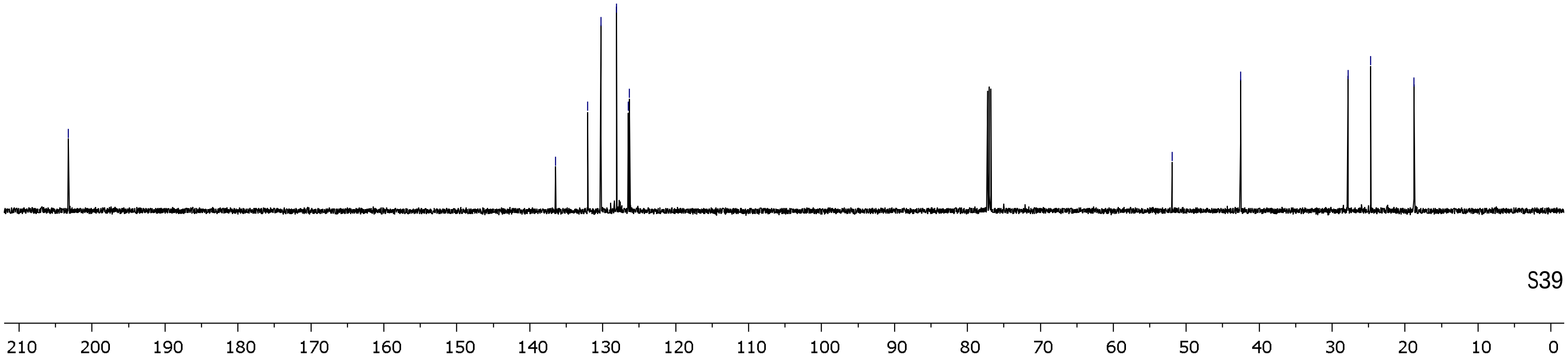

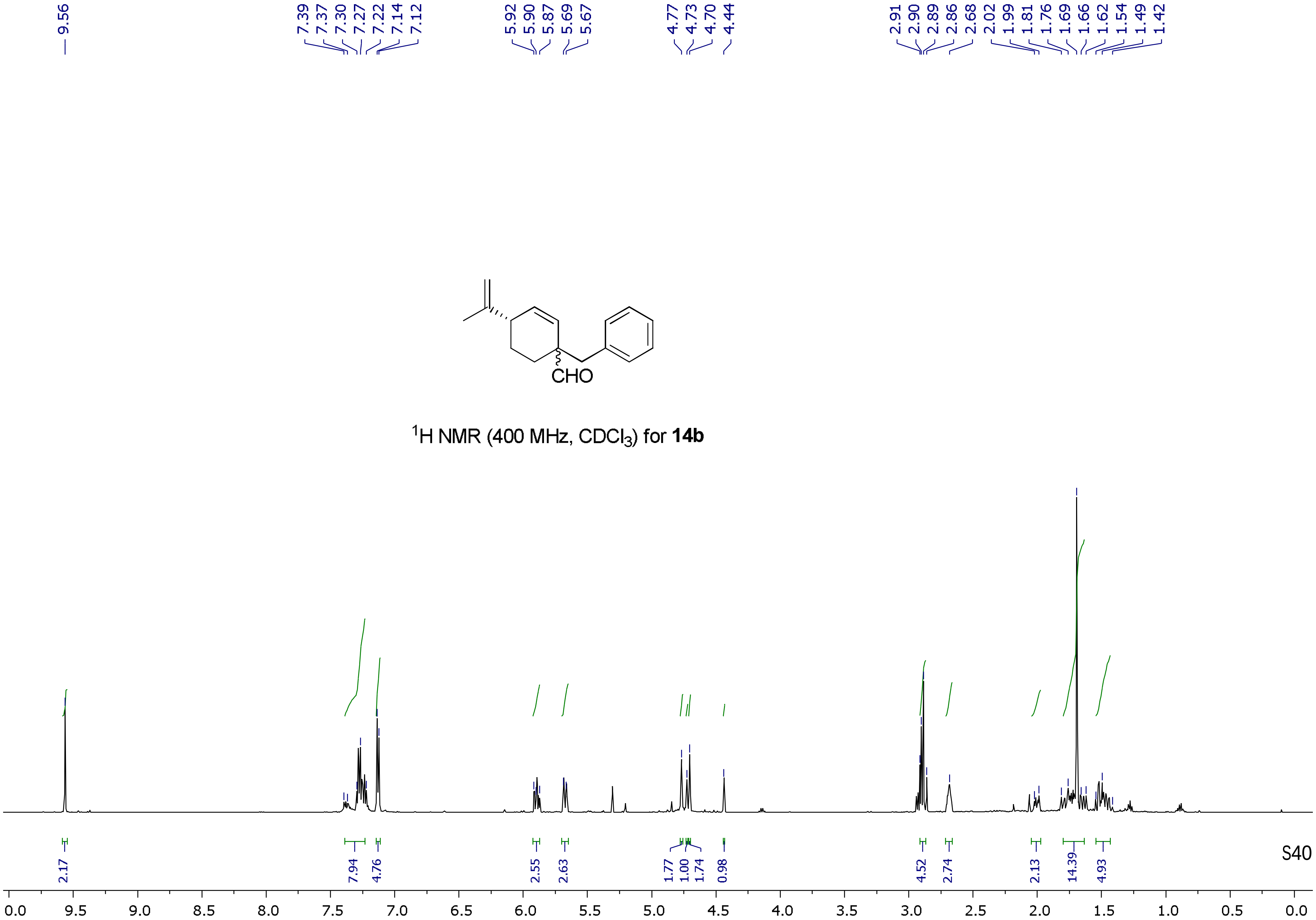

^{1}H NMR (400 MHz, $CDCl_3$) for **14b**

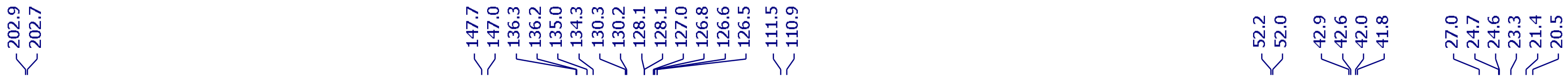

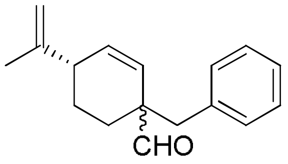

$^{13}C\{^{1}H\}$ NMR (100 MHz, $CDCl_3$) for **14b**

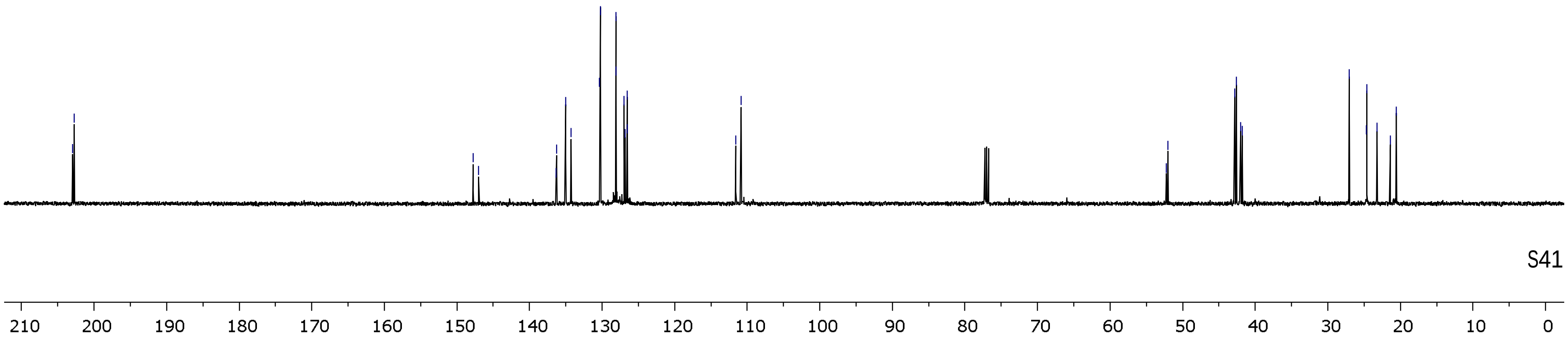

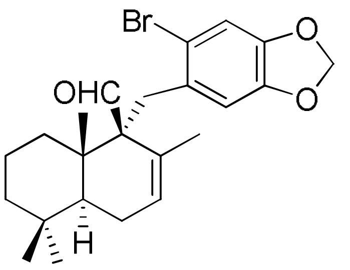

^{1}H NMR (400 MHz, $CDCl_3$) for **14c**

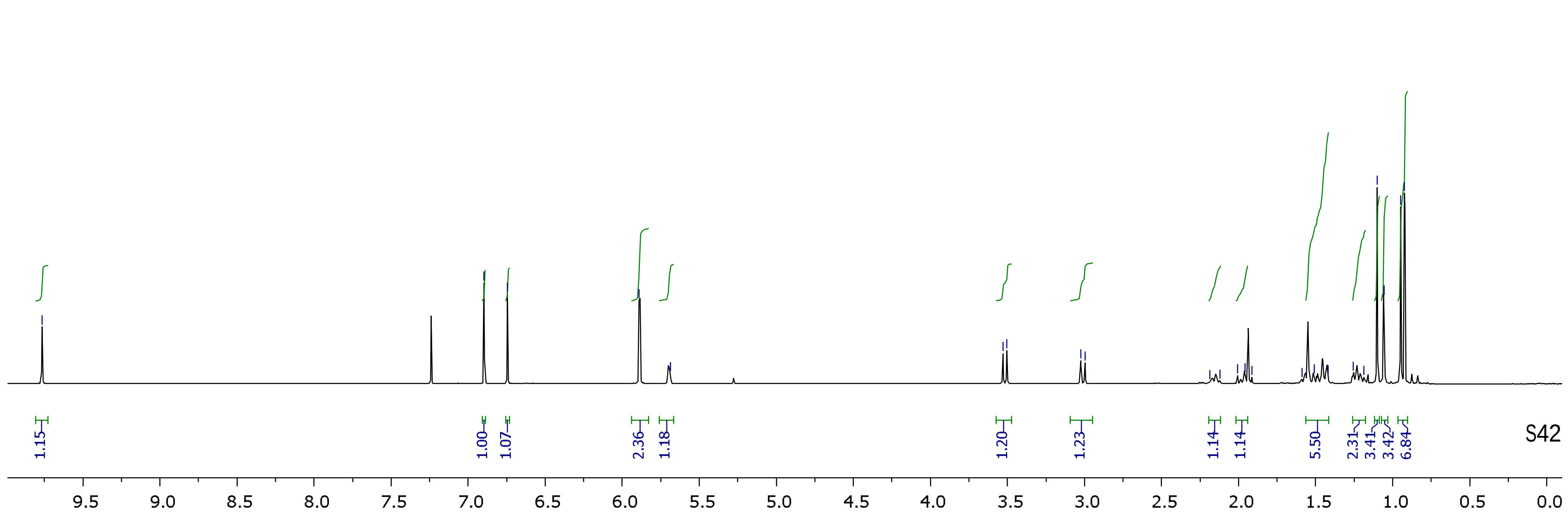

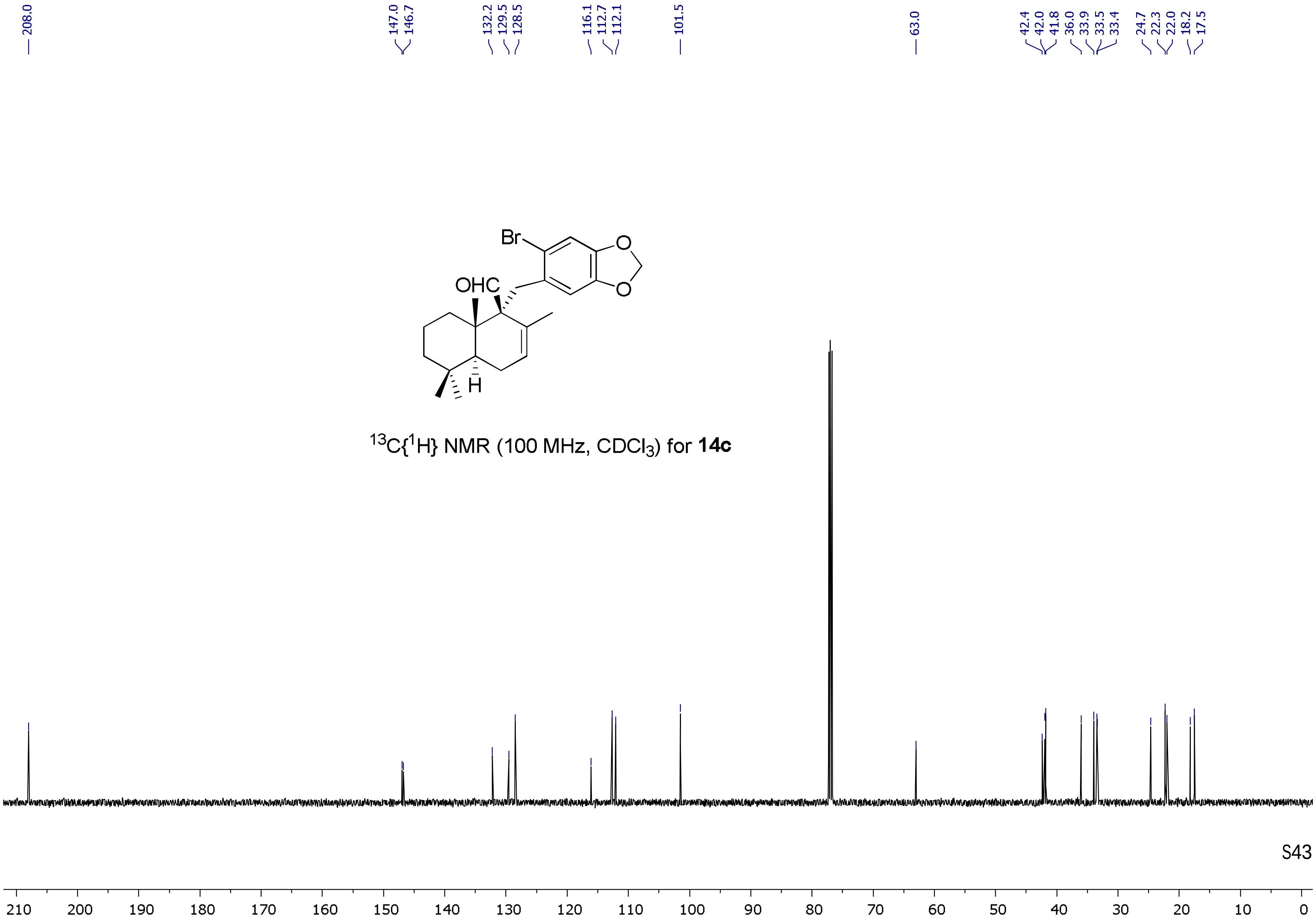

$^{13}C\{^1H\}$ NMR (100 MHz, $CDCl_3$) for **14c**

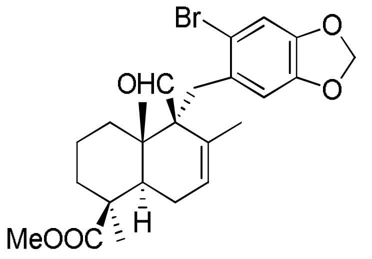

^{1}H NMR (400 MHz, CDCl$_3$) for **14d**

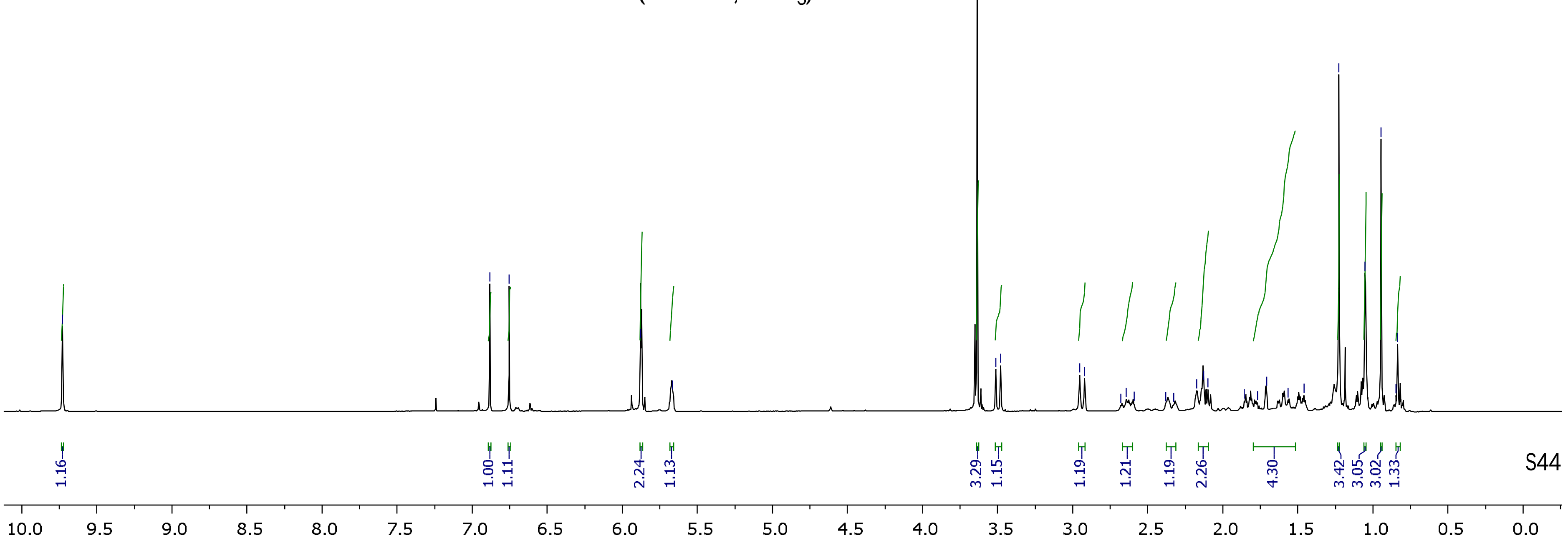

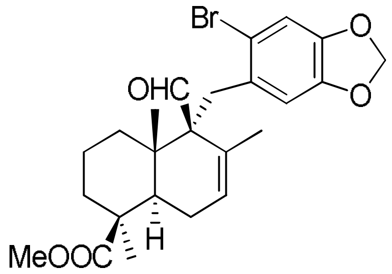

$^{13}C\{^{1}H\}$ NMR (100 MHz, $CDCl_3$) for **14d**

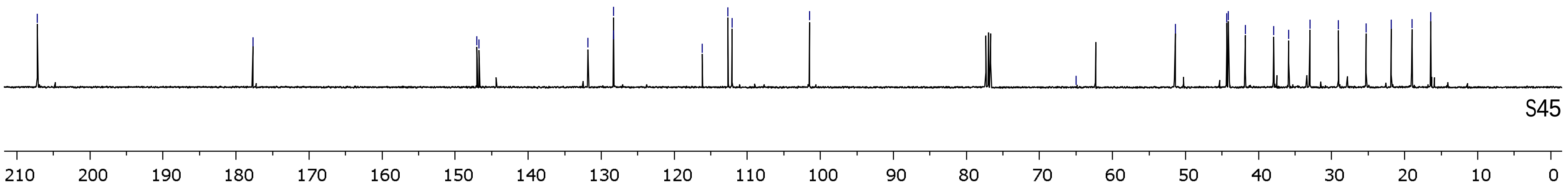

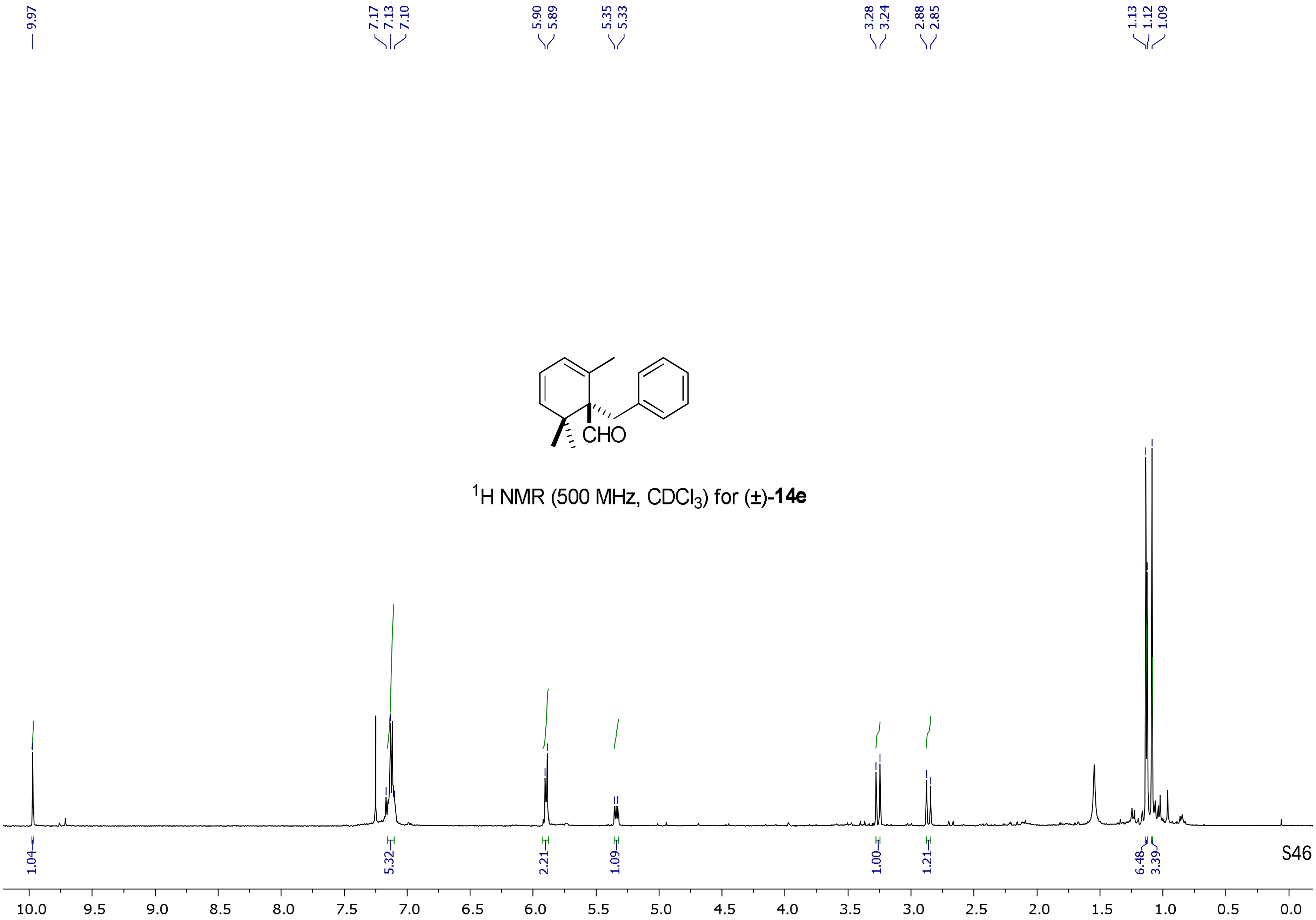

^{1}H NMR (500 MHz, $CDCl_3$) for (±)-**14e**

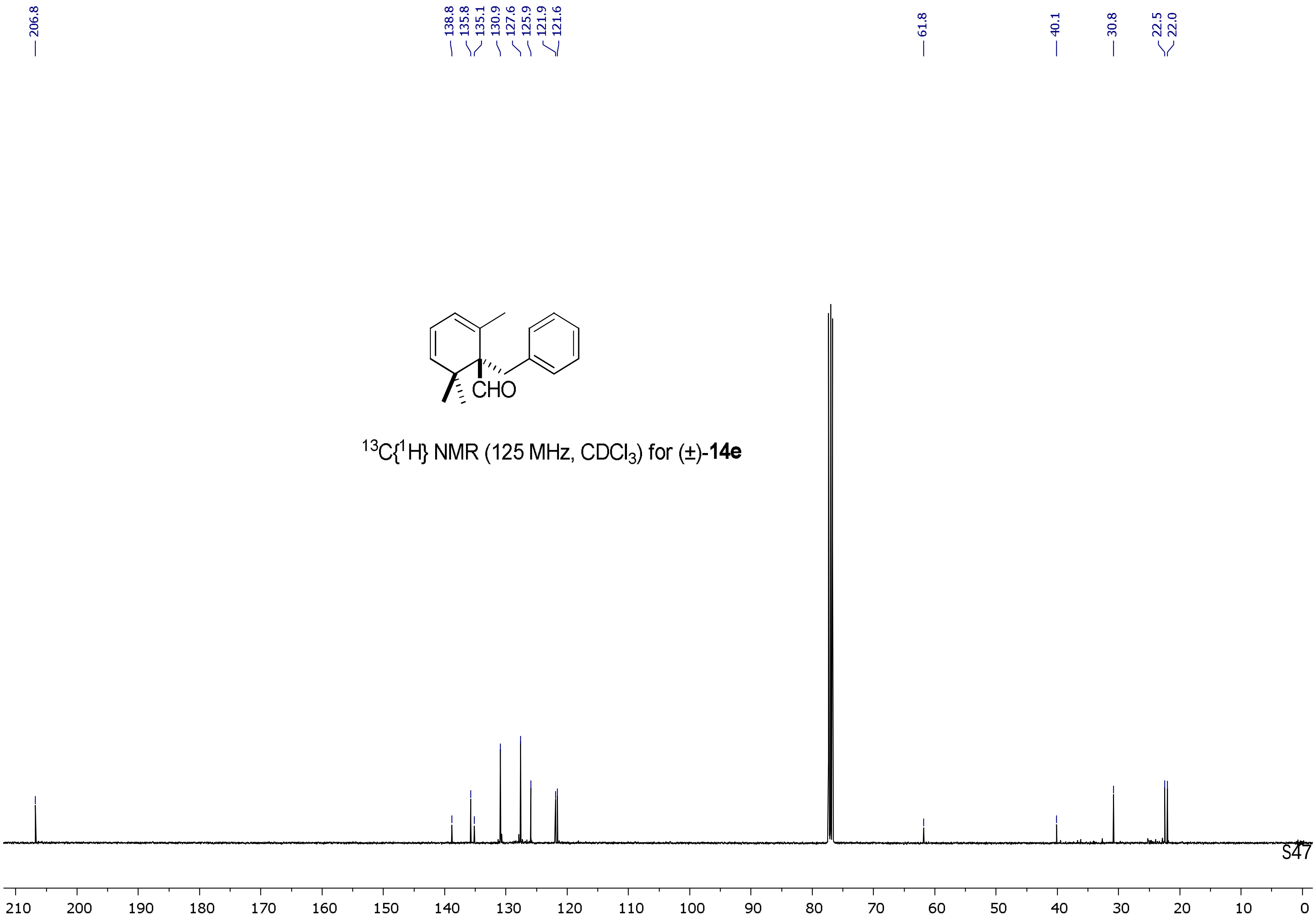

$^{13}C\{^{1}H\}$ NMR (125 MHz, $CDCl_3$) for (±)-**14e**

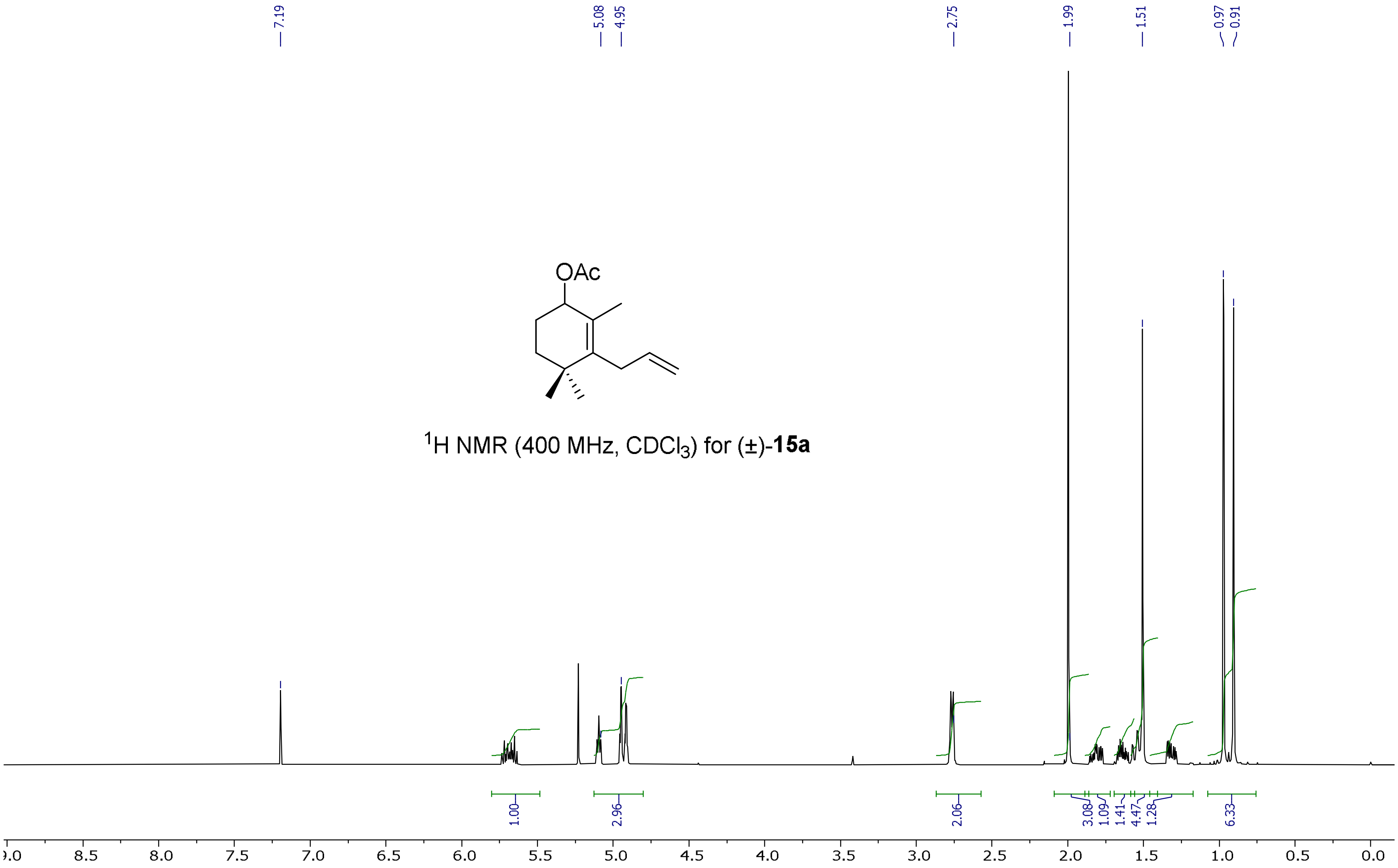
7.19
5.08
4.95
2.75
1.99
1.51
0.97
0.91
OAc
1H NMR (400 MHz, CDCl3) for (±)-15a
1.00
2.96
2.06
3.08
1.09
1.41
4.47
1.28
6.33
9.0 8.5 8.0 7.5 7.0 6.5 6.0 5.5 5.0 4.5 4.0 3.5 3.0 2.5 2.0 1.5 1.0 0.5 0.0

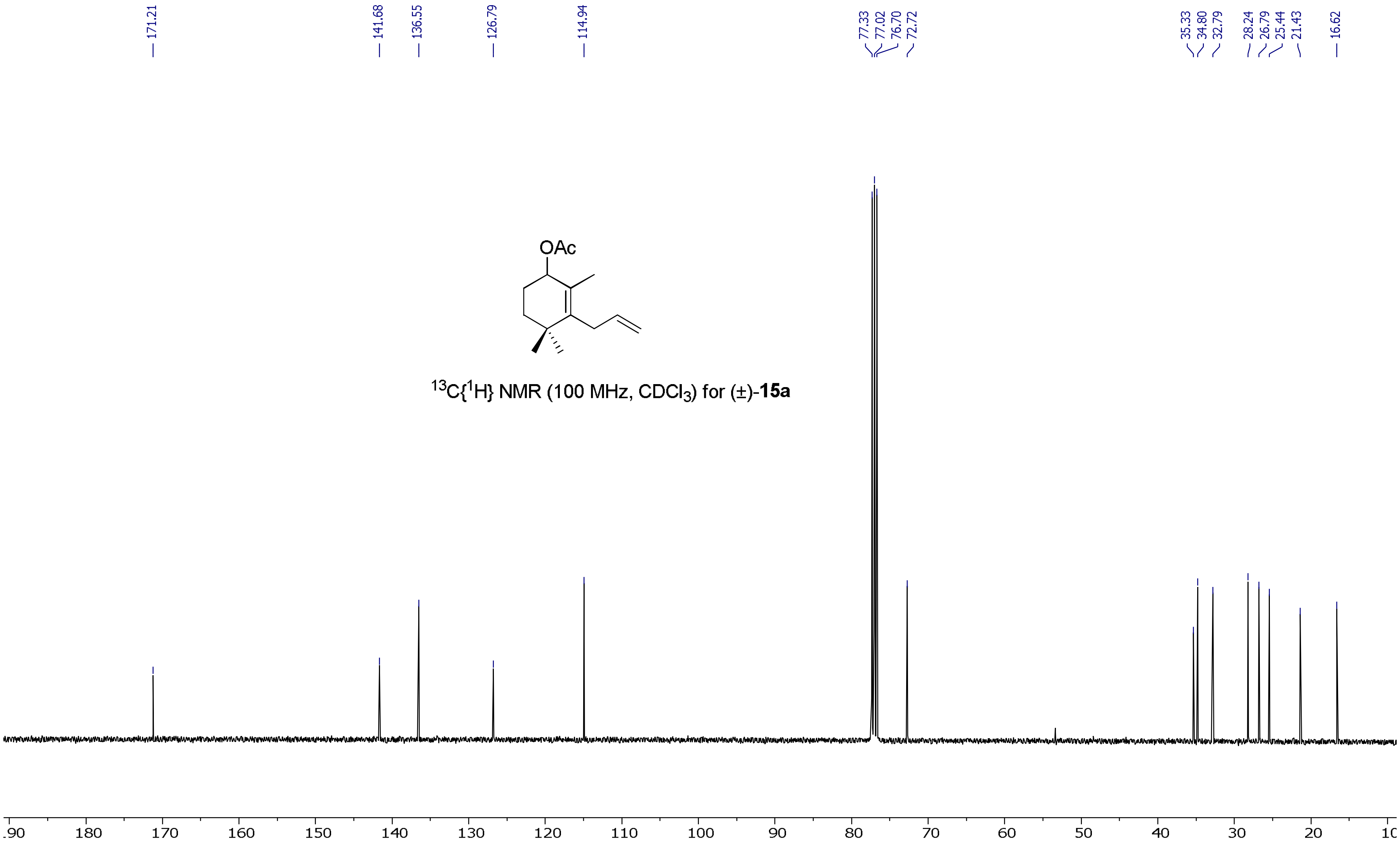

$^{13}C\{^1H\}$ NMR (100 MHz, $CDCl_3$) for (±)-**15a**

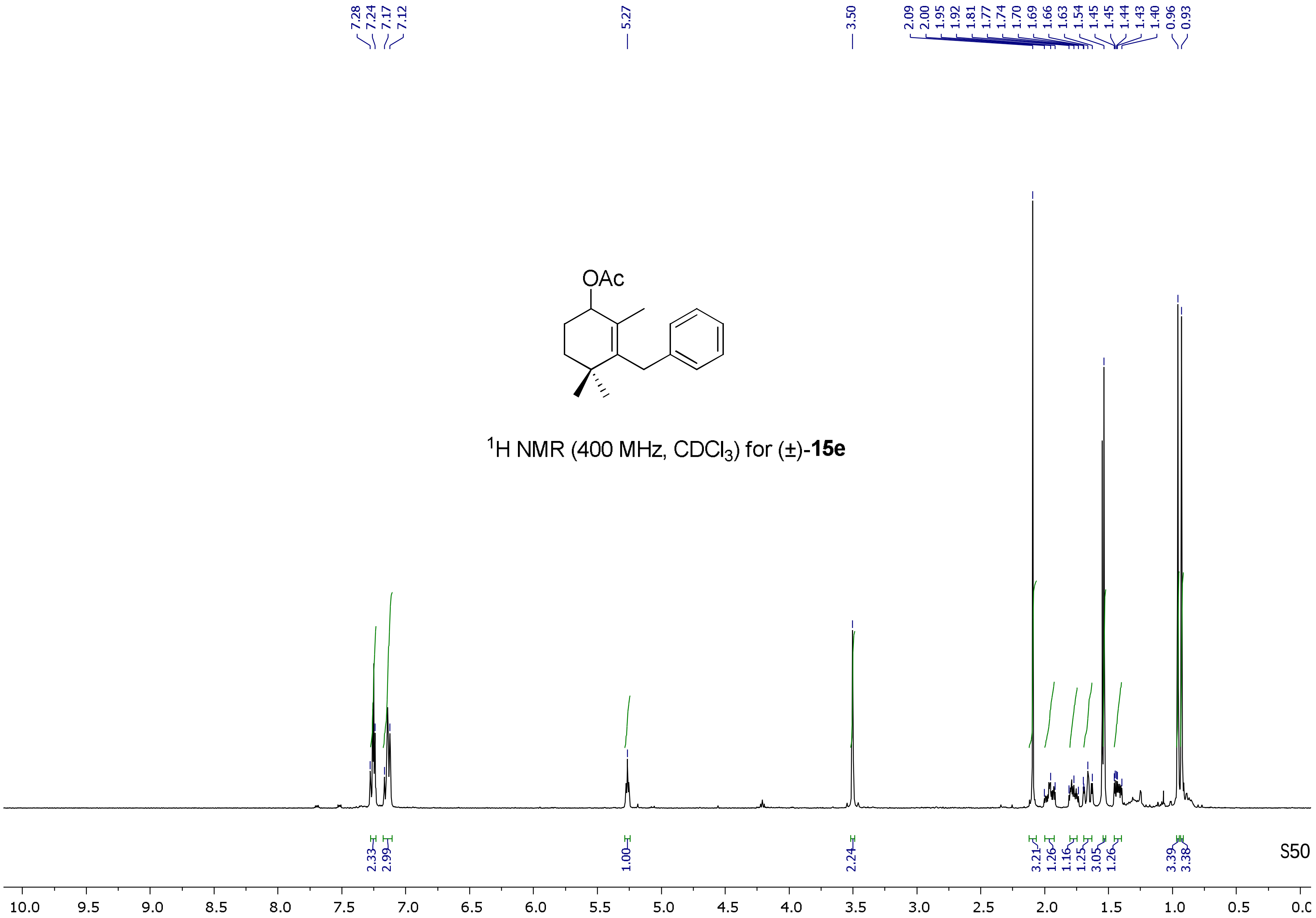

^{1}H NMR (400 MHz, $CDCl_3$) for (±)-**15e**

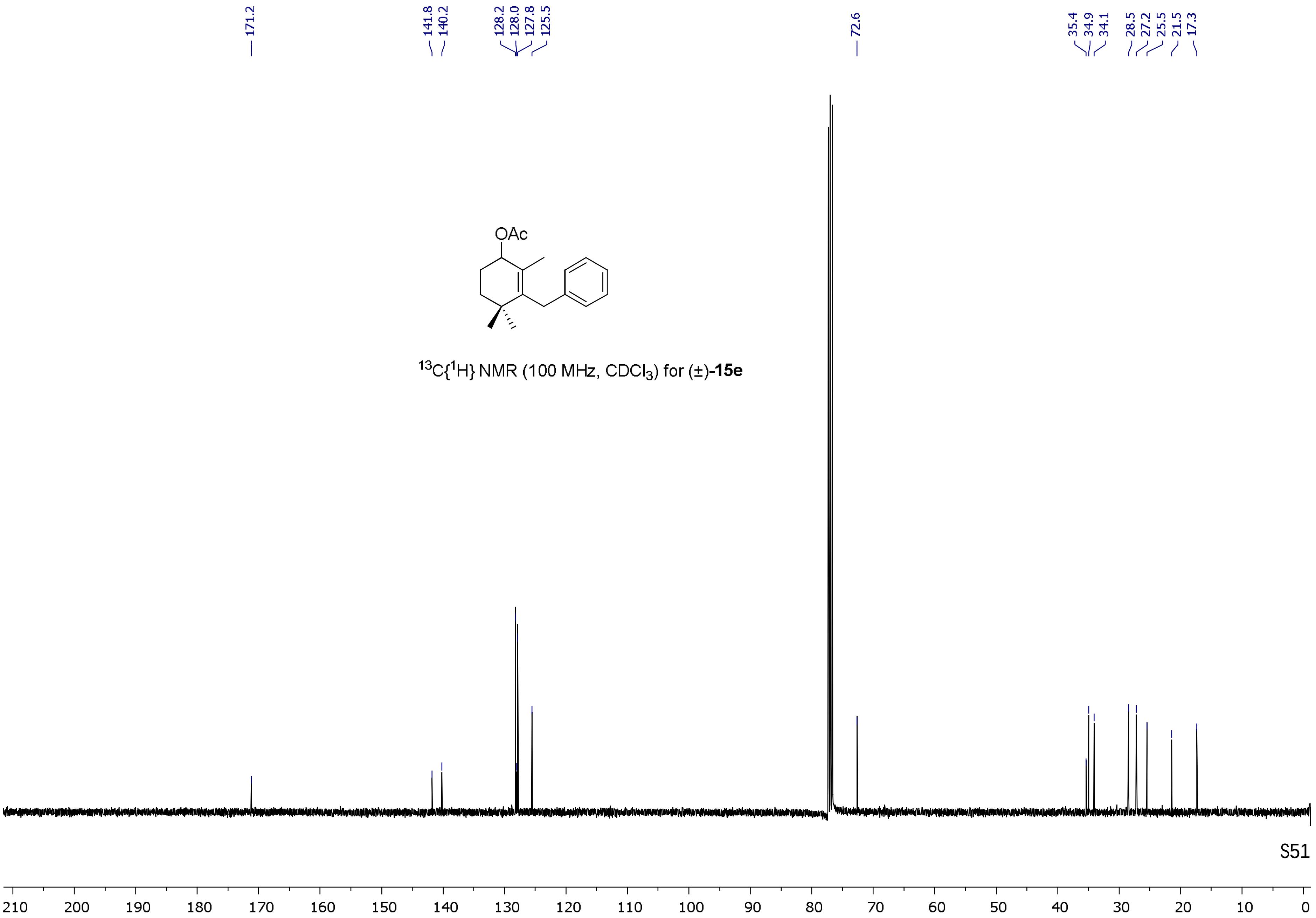

$^{13}C\{^{1}H\}$ NMR (100 MHz, $CDCl_3$) for (±)-**15e**

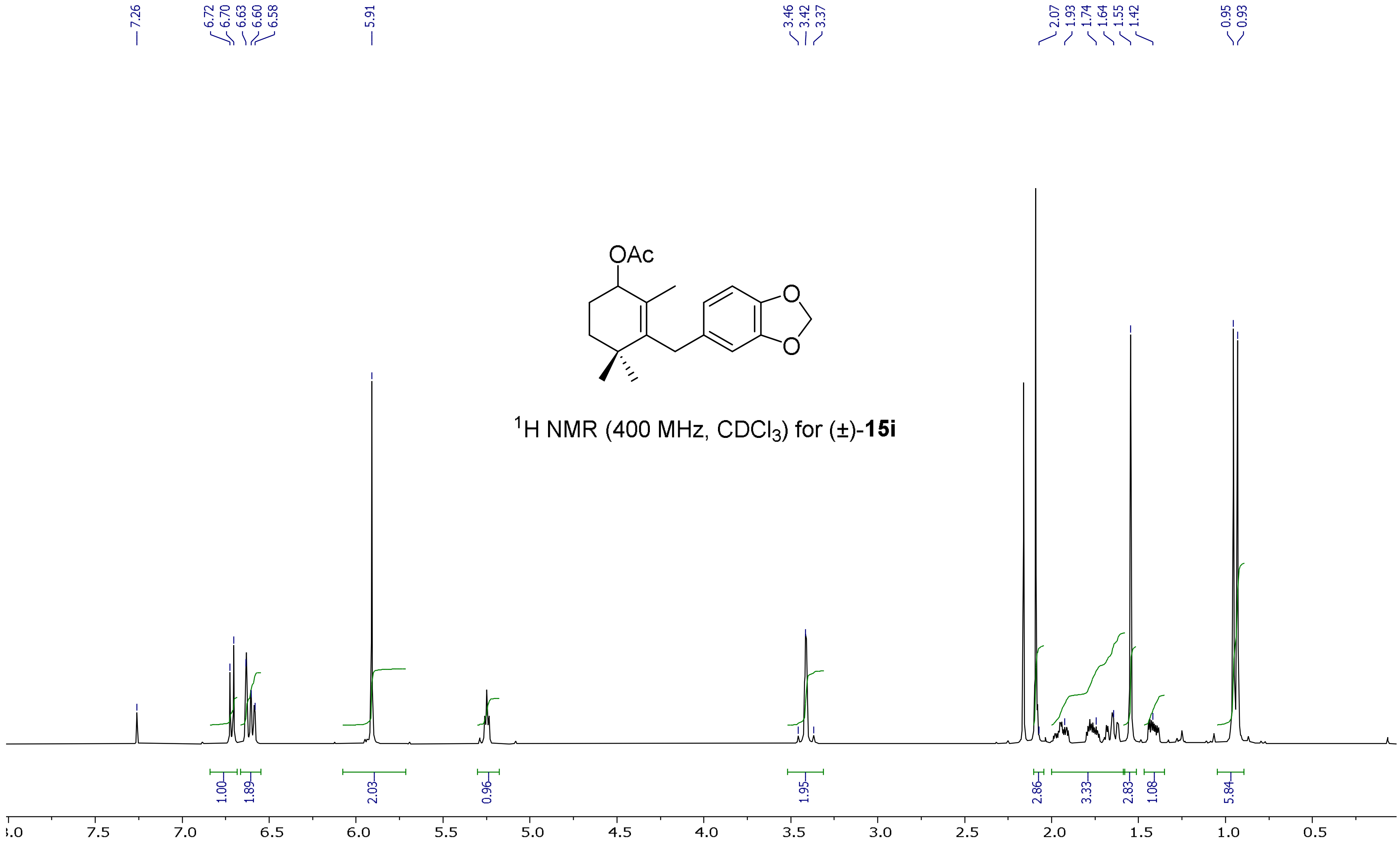

^{1}H NMR (400 MHz, $CDCl_3$) for (±)-**15i**

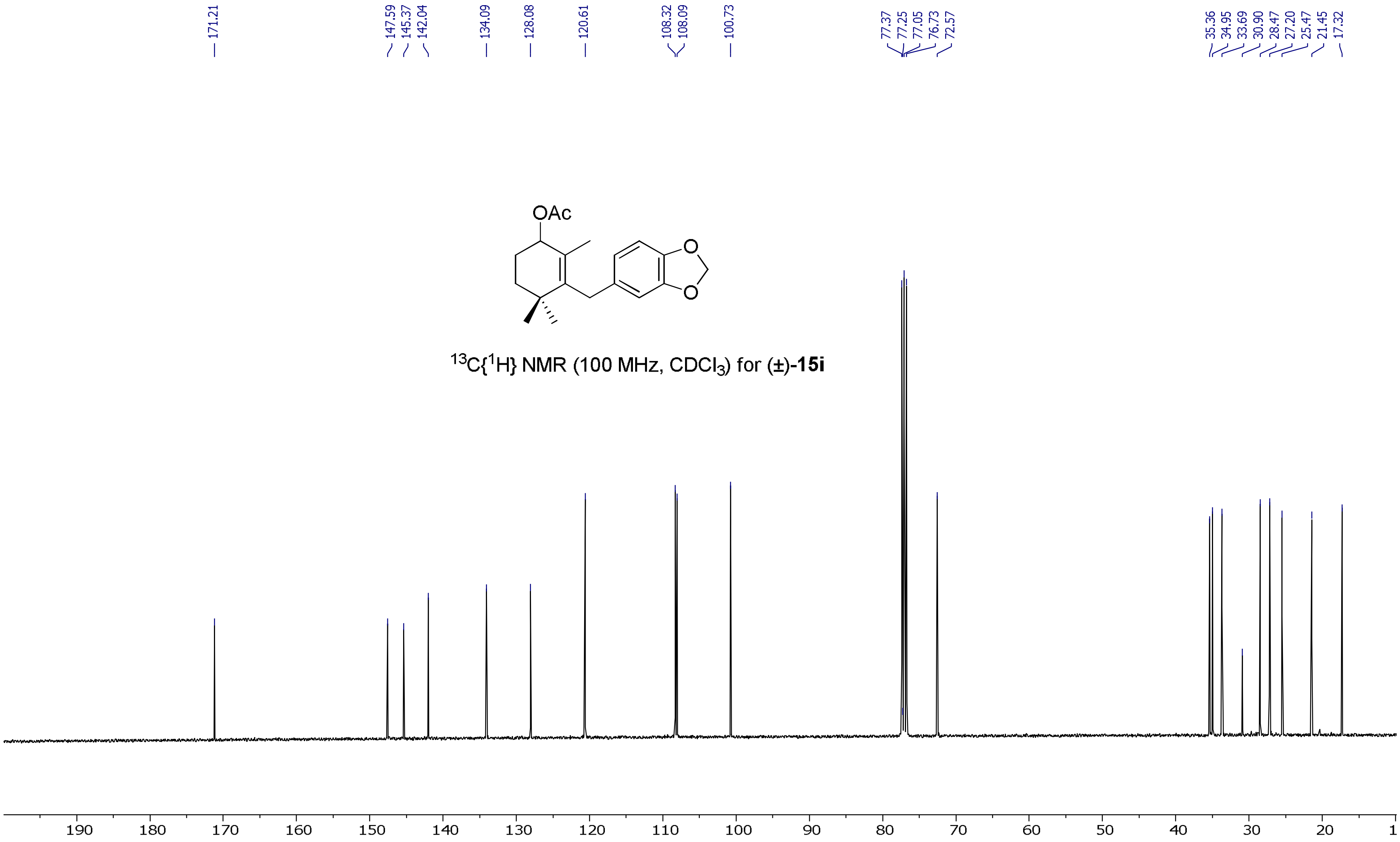

$^{13}C\{^1H\}$ NMR (100 MHz, $CDCl_3$) for (±)-**15i**

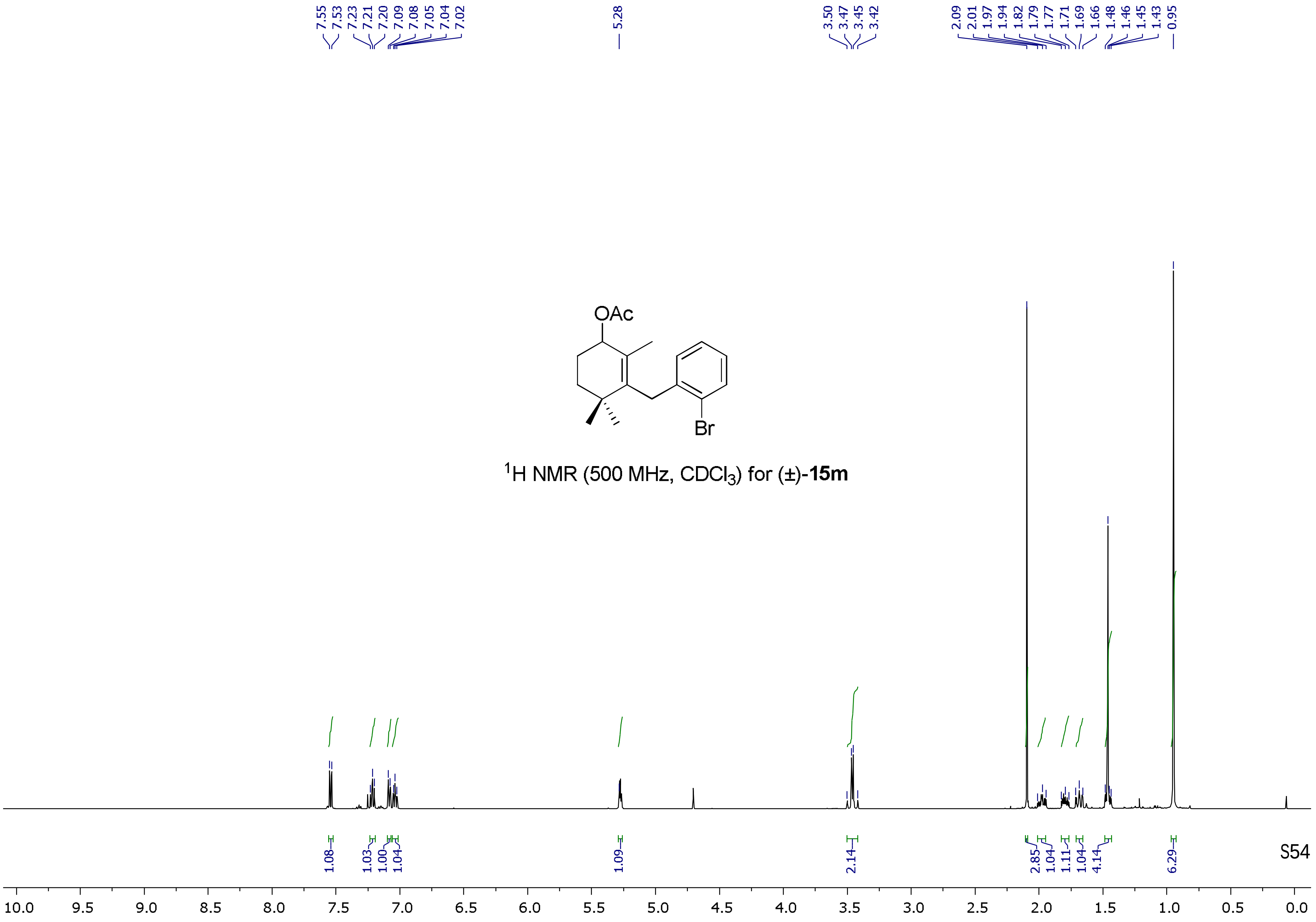
7.55
7.53
7.23
7.21
7.20
7.09
7.08
7.05
7.04
7.02
5.28
3.50
3.47
3.45
3.42
2.09
2.01
1.97
1.94
1.82
1.79
1.77
1.71
1.69
1.66
1.48
1.46
1.45
1.43
0.95
OAc
Br
1H NMR (500 MHz, CDCl3) for (±)-15m
1.08
1.03
1.00
1.04
1.09
2.14
2.85
1.04
1.11
1.04
4.14
6.29
10.0
9.5
9.0
8.5
8.0
7.5
7.0
6.5
6.0
5.5
5.0
4.5
4.0
3.5
3.0
2.5
2.0
1.5
1.0
0.5
0.0

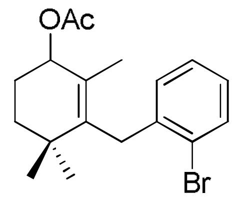

$^{13}C\{^1H\}$ NMR (125 MHz, $CDCl_3$) for (±)-**15m**

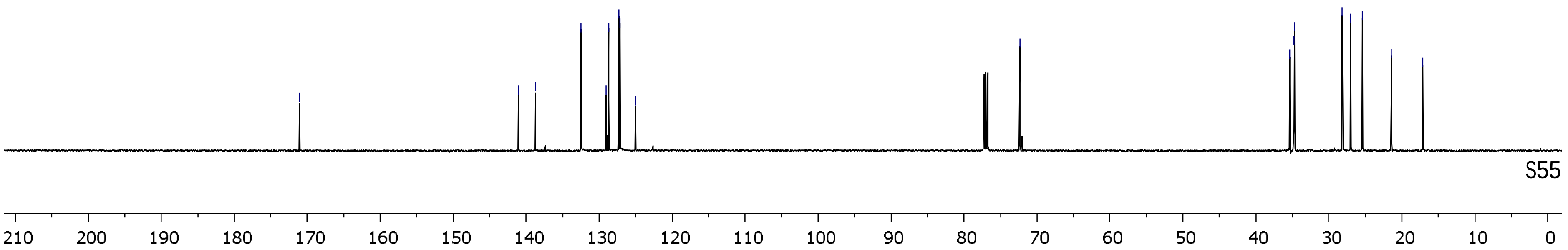

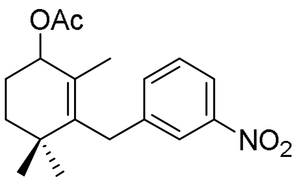

^{1}H NMR (400 MHz, $CDCl_3$) for (±)-**15o**

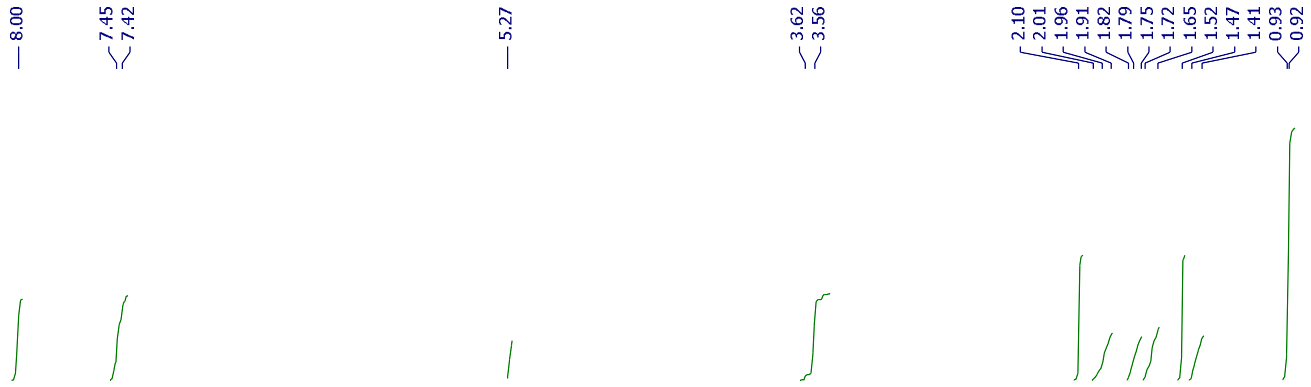

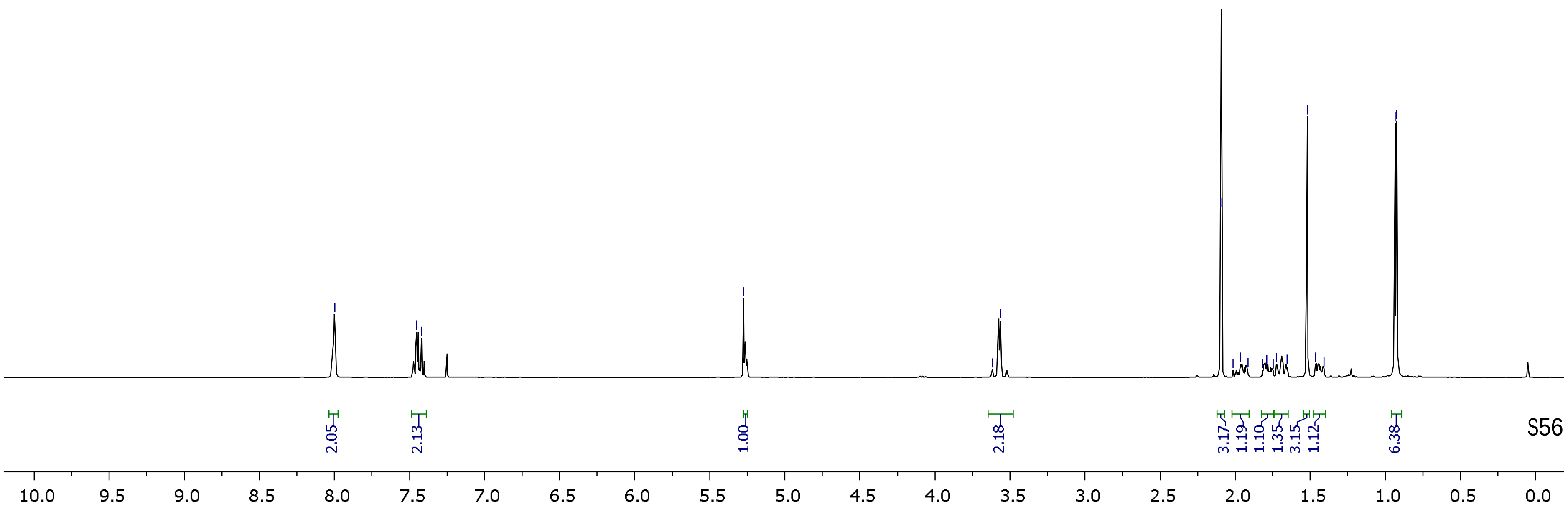

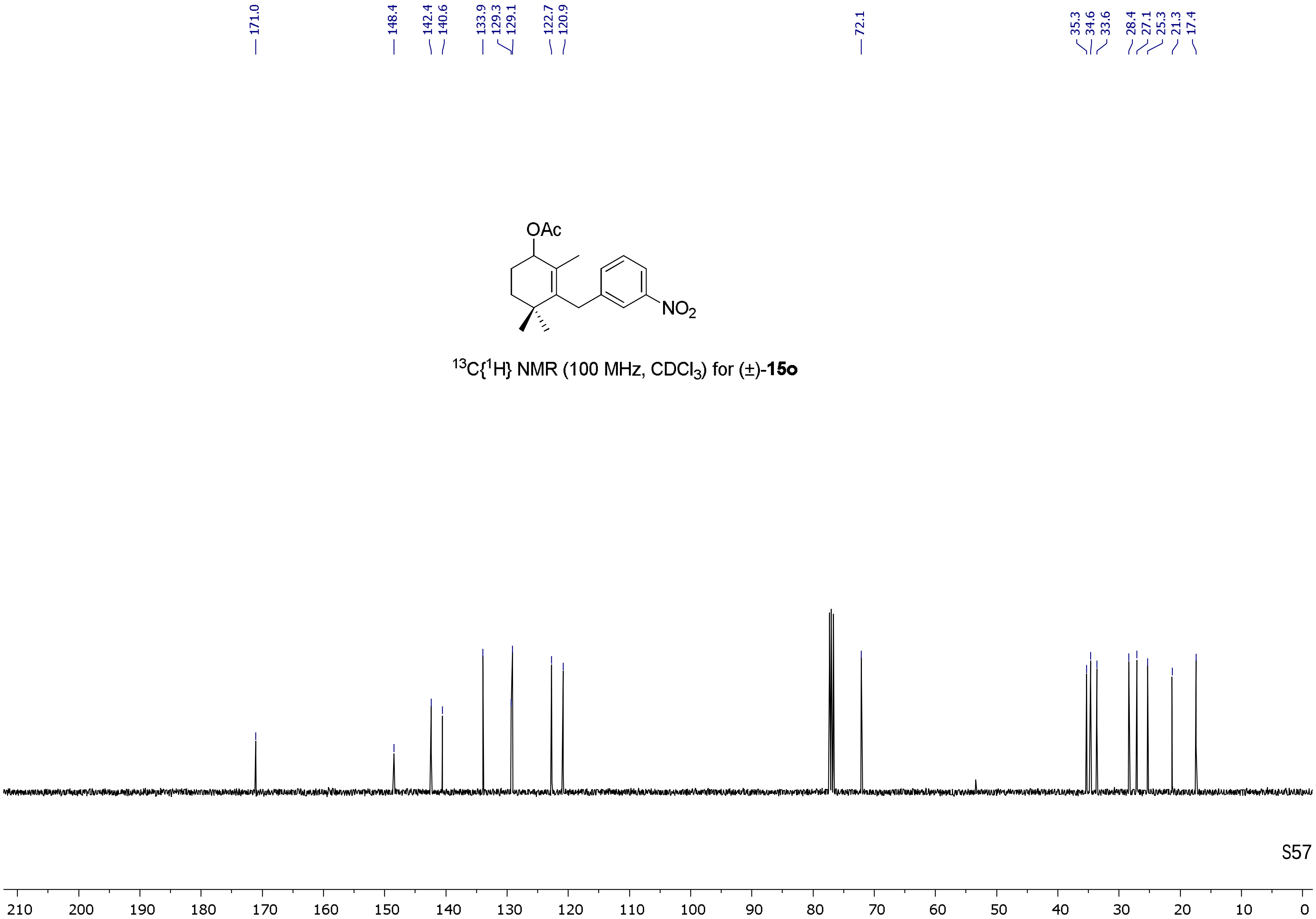

$^{13}C\{^{1}H\}$ NMR (100 MHz, $CDCl_3$) for (±)-**15o**
